# Identifying and ranking causal microbial biomarkers for colorectal cancer at different cancer subsites and stages: a Mendelian randomization study

**DOI:** 10.3389/fonc.2023.1224705

**Published:** 2023-07-19

**Authors:** Hongfeng Li, Dashuang Sheng, Chuandi Jin, Guoping Zhao, Lei Zhang

**Affiliations:** ^1^ Department of Biostatistics, School of Public Health, Cheeloo College of Medicine, Shandong University, Jinan, China; ^2^ Microbiome-X, National Institute of Health Data Science of China, Cheeloo College of Medicine, Shandong University, Jinan, China; ^3^ State Key Laboratory of Microbial Technology, Shandong University, Qingdao, China; ^4^ CAS Key Laboratory of Computational Biology, Bio-Med Big Data Center, Shanghai Institute of Nutrition and Health, University of Chinese Academy of Sciences, Chinese Academy of Sciences, Shanghai, China; ^5^ Shandong Children’s Microbiome Center, Children’s Hospital Affiliated to Shandong University, Jinan, China

**Keywords:** colorectal cancer, gut microbiota, Mendelian randomization analysis, dietary habit, causal microbial biomarkers

## Abstract

**Introduction:**

The gut microbiome is directly involved in colorectal carcinogenesis, but much of the epidemiological evidence for the effect of the gut microbiome on colorectal cancer (CRC) risk comes from observational studies, and it is unclear whether identified microbial alterations are the cause or consequence of CRC development.

**Methods:**

Univariate Mendelian randomization (MR) analysis and multivariate MR analysis based on Bayesian model averaging were performed to comprehensively explore the microbial risk factors associated with CRC. The Network Module Structure Shift method was used to identify microbial biomarkers associated with CRC. Mediation analysis was used to explore the dietary habits-microbiota-CRC pathway.

**Results:**

The results of the four methods showed that 9 bacteria had a robust causal relationship with the development of CRC. Among them, *Streptococcus thermophilus* reduced the risk of CRC; *Eubacterium ventriosum* and *Streptococcus* were beneficial bacteria of malignant tumors of colon (CC); Erysipelotrichaceae was a protective factor for malignant tumors of rectal (CR); *Bacteroides ovatus* was a risk factor for benign tumors. Finally, the mediation analysis revealed 10 pathways by which dietary regulation bacteria affected the risk of CRC, including alcohol consumption increased the risk of CC by reducing the abundance of *Eubacterium ventriosum* (mediated proportion: 43.044%), and the mediated proportion of other pathways was 7.026%-34.22%.

**Discussion:**

These findings will contribute to the understanding of the different carcinogenic mechanisms of intestinal flora in the colon and rectum and the risk of tumor transformation, thereby aiding CRC prevention, early screening, and the development of future strategies to reduce CRC risk.

## 1 Introduction

Colorectal cancer (CRC) is the third most common cancer(1), and it is not a single entity, colon and rectal cancers have their own characteristics in terms of genetics, anatomy, treatment methods, and metastasis patterns (2, 3). Most CRC tumors are thought to be caused by precancerous changes in the adenoma-cancer pathway (4). Screening and removal of colorectal adenomas in asymptomatic individuals can reduce CRC morbidity and mortality (5).

The relationship between intestinal microecology and the occurrence and development of CRC has attracted more and more attention. First, a large number of population studies have found significant differences in the gut microbiome between people with CRC and healthy people (6–8). Further cohort studies showed that gut microbiota composition is different at different stages of CRC and that the interaction between intestinal flora gradually complicates with the progression of the disease (6, 9, 10). These results suggest that changes in bacteria play a driving role in the initiation and progression of CRC. Experimental evidence also supports the role of bacteria in CRC (11–14). In addition, the gut microbiota can be rapidly altered by diet, and people who eat different diets have significantly different gut microbial compositions, which in turn are associated with different CRC risks. For example, fat consumption and red meat intake are related to the abundance of sulfide bacteria (15).

Most relevant studies were observational, and it was difficult to draw causal conclusions. While animal experiments can verify the specific mechanism by which a small number of bacteria respond to CRC, it is difficult to screen out bacteria with truly causal effects from tens of thousands of bacteria. Mendelian randomization (MR) can use single nucleotide polymorphisms (SNPs) as instrumental variables (IVs) to establish causal relationships between exposure and outcomes (16). The two-sample multivariate MR method based on Bayesian model averaging (MR-BMA) can detect true causal risk factors when candidate risk factors are highly correlated (17). Gut microbial traits are strongly correlated and high-throughput, so MR-BMA method is a suitable method to find microbial risk factors associated with disease. What’s more, a non-MR method, Network Module Structure Shift (NetMoss) (18), can identify microbial biomarkers associated with various diseases.

Currently, only a few bacteria have clearly demonstrated a causal relationship with CRC (11–14). Therefore, univariate MR, MR-BMA, and NetMoss methods were used to identify causal bacteria for different cancer subsites (colorectal, colon, and rectum) and stages (benign tumors and malignant tumors) of CRC. Given that gut microbiota can be rapidly altered by diet, we performed a two-step MR analysis to investigate the causal pathway from dietary habits to CRC by bacteria.

## 2 Materials and methods

### 2.1 Data sources

We collected GWAS statistics related to gut microbiome from the Netherlands, including 207 microbial taxa (5 phyla, 10 classes, 13 orders, 26 families, 48 genera, and 105 species) and 205 functional pathways (**Table 1**) (19). The GWAS summary statistics of CRC at different cancer subsites and stages (**Table 1**) were obtained from the FinnGen biobank (https://r4.finngen.fi/) (20).Colorectal cancer data include: colorectal cancer (CRC, *N* = 221814); malignant tumors of the colon (CC, *N* = 220595); malignant tumors of the rectal (CR, *N* = 219870); benign tumors of the colorectum (BCR, *N* = 228104); benign tumors of the colon (BC, *N* = 218792); benign tumors of the rectal (BR, *N* = 220900) (**Table 1**).The different phenotypes of colorectal cancer are defined according to International Classification of Diseases (ICD) codes retrieved from the Finnish National Registry. And GWAS summary statistics for 14 dietary habits and 6 non-CRC diseases were collected from the publicly available GWAS summary statistics database (https://gwas.mrcieu.ac.uk/) (**Table 1**), published by the Medical Research Council of the University of Bristol Medical Research Council’s Integrated Epidemiology Unit (MRC IEU) (21). Detailed quality control, filling, and GWAS details have been described elsewhere previously (19–21). The above data were mainly used for MR analysis. And we built a multi-population cohort (**Table S1**) for further non-MR validation analyses. Details on how the cohort was constructed are provided in the **Supplementary Material**.

**Table 1:** Details of GWAS data for analysis.

	Trait	Sample size	GWAS ID^1^	Consortium	Population
Malignant tumors	Colorectal cancer	221814	finn-b-C3_COLORECTAL	FinnGen	European
	Malignant tumors of colon	220595	finn-b-C3_COLON	FinnGen	European
	Malignant tumors of rectal	219870	finn-b-C3_RECTUM	FinnGen	European
Benign tumors	Benign tumors of colorectum	228104	finn-b-CD2_BENIGN_COLORECANI	FinnGen	European
	Benign tumors of colon	218792	finn-b-CD2_BENIGN_COLON	FinnGen	European
	Benign tumors of rectal	220900	finn-b-CD2_BENIGN_RECTUM	FinnGen	European
Gut microbiota	207 microbial tax	7738	NA^2^	NA	European
	205 microbial functional pathways	7738	NA	NA	European
Five non-colorectal cancer diseases	Crohn’s disease (Large bowel, Small bowel)	211107,211268	finn-b-CHRONLARGE, finn-b-CHRONSMALL	FinnGen	European
	Ulcerative colorectitis	214620	finn-b-K11_ULCER	FinnGen	European
	Irritable bowel syndrome	187028	finn-b-K11_IBS	FinnGen	European
	Non-alcoholic fatty liver disease	218792	finn-b-NAFLD	FinnGen	European
	Type 2 diabetes	215654	finn-b-E4_DM2	FinnGen	European
Dietary habits	Alcohol drinker status: Current	360726	ukb-d-20117_2	NA	European
	Bread intake	452236	ukb-b-11348	MRC-IEU	European
	Cereal type: Biscuit cereal (e.g. Weetabix)	299898	ukb-d-1468_2	NA	European
	Lamb/mutton intake	460006	ukb-b-14179	MRC-IEU	European
	Ferritin	23986	ieu-a-1050	GIS	European
	Liver intake	64944	ukb-b-6373	MRC-IEU	European
	Milk type used: Never/rarely have milk	360806	ukb-d-1418_6	NA	European
	Mineral and other dietary supplements: Calcium	336314	ukb-a-495	Neale Lab	European
	Mineral and other dietary supplements: Glucosamine	336314	ukb-a-494	Neale Lab	European
	Mineral and other dietary supplements: Zinc	461384	ukb-b-13891	MRC-IEU	European
	Single crust pastry intake	64949	ukb-b-2024	MRC-IEU	European
	Type of special diet followed: Gluten-free	64949	ukb-b-11189	MRC-IEU	European
	Vitamin and mineral supplements: Multivitamins +/- minerals	335591	ukb-a-464	Neale Lab	European
	Vitamin and mineral supplements: Vitamin C	460351	ukb-b-15175	MRC-IEU	European

^1^ ID in the MRC IEU OpenGWAS database. ^2^ NA, Not Applicable.

### 2.2 Instruments variable selection

According to the previous studies, SNPs with low significance thresholds have the largest explanatory variance for microbial traits(22, 23), so we set the thresholds (*P* < 1×10^-5^) to select the IVs. Palindromic SNPs with non-derived allele frequencies (minor allele frequency (MAF) > 0.3) were excluded. If they were correlated with *R* > 0.01 within a 10,000 kb window, they were considered SNPs in linkage disequilibrium (*LD*) and should be excluded. *F*-statistics were computed to quantify the strength of IVs. IVs with *F*-statistics < 10 were considered weak IVs and were excluded. What’s more, we searched each SNP in the PhenoScanner GWAS database to detect possible pleiotropy (24).

### 2.3 Univariable MR analysis

Univariate MR was used to assess the causal relationship between gut microbiota and CRC at different cancer subsites and stages and 6 non-CRC diseases. MR analyses were performed and reported in accordance with the STROBE-MR guidelines (25, 26). a list of the STROBE-MR guidelines can be found in **Supplementary File**. The inverse variance weighted (multiplicative random effects) [IVW(M)] method is mainly used (27). For exposures for which only 1 IV could be identified, the Wald ratio is used to estimate its causal effect (28). We used *P* < 0.05 as the potential significance threshold. We also derived false discovery rate (*FDR*)-corrected *P*-values with the Benjamini-Hochberg (*BH*) method and used *P_fdr_* < 0.2 as the *FDR*-corrected significance threshold. We used a threshold of *P* < 5×10^-8^ to select other traits-related IVs, and used the same method to explore the causal relationship between diet and other traits.

A series of additional analyses were conducted to assess the reliability of the results. The MR Steiger test was used to estimate the possible direction of causality between microbial traits and outcome. For microbial traits that have a potential causal effect on outcomes, we applied (“coloc”) to check whether the variation responsible for influencing these factors was the same variation that influences the outcome (29). If the threshold value of PP.H4 > 0.8, it is considered that MR hypothesis was violated. In addition, Cochran Q statistics were used to assess the global heterogeneity of the selected SNPS. MR-Egger regression was used to capture horizontal pleiotropy (30). Finally, we used MR-PRESSO to detect and correct potential outliers (31).

### 2.4 Potential biomarkers ranking

MR-BMA is a multivariate MR method that prefers causal risk factors from high-dimensional candidate risk factors in a Bayesian framework (17). Like conventional MVMR (32), multiple exposures using overlapping IVs allow adequate handling of “pleiotropism of measurements” (17).

We used MR-BMA to rank agnostic causal importance for several microbial markers that had a potential causal relationship with outcomes in univariate MR analysis (*P* < 0.05). All independent genetic variants strongly associated with any biomarker (*P* < 1×10^-5^) were included in the analysis (CRC: 87; CC: 135; CR: 125; BCR: 103; BC: 101; BR: 158). Genetic associations between biomarkers were then examined, and biomarkers with genetic associations greater than 0.985 were deleted. The marginal inclusion probability (MIP) (i.e., the sum of the posterior probabilities of the model in which the risk factor exists) and the model average causal effect (MACE) (representing a conservative estimate of the direct causal effect of the risk factor on the average outcome of these models) of each risk factor are calculated in the model. For all BMA analyses, we set z to 10,000, prior probability to 0.1, and prior variance (σ) to 0.5. A sensitivity analysis of this part is provided in the **Supplementary Material**. Full details of the MR-BMA method can be found elsewhere (17).

### 2.5 Microbial risk factors identification

The gut microbiota showed significant correlations, both phenotypically and genetically (**Figure S1**). MR–BMA provides a method that allows multiple microbial traits to be modeled together. This approach allows related microbial traits to be disentangled to identify which may be driving the “true” causal signal over others. MR-BMA can adequately handle the “measured pleiotropy” as well as traditional multivariate MR (17), but compared with traditional MVMR (32), MR-BMA is especially suitable for high-throughput and highly correlated data. We used MR-BMA to identify exposures that were truly causally associated with outcomes from a high dimensional set of related candidate risk factors, 207 the gut microbiota. The analysis method was largely consistent with the potential biomarkers ranking analysis, as detailed in the **Supplementary Materials**. The top ten bacteria with MIP were interpreted as the strongest “true” causal candidates of all the bacteria provided in the model. In the sensitivity analysis, the *pp* threshold for *Cd* to identify strong influence points is shown in **Table S2**.

### 2.6 NetMoss analysis

The ASV data processing method for multi-population cohorts is described in the **Supplementary Material**. Subsequently, Sparcc was used to analyze the correlation of ASV-level bacteria to obtain correlation coefficient tables for different phenotypes (healthy, adenoma, and CRC) in each cohort. Using the relative abundance tables and correlation coefficient tables of different phenotypes in different cohorts, we further utilize the Sparcc (33) network module-based NetMoss2 (18) method to effectively reduce the batch effect while assessing the importance of bacteria between colorectal adenomas and healthy people or CRC and healthy people.

### 2.7 Mediation analysist

**Figure 1:**
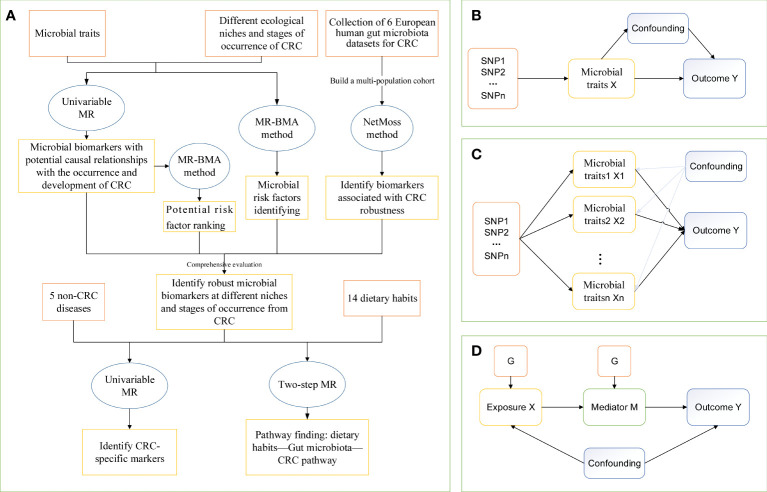
The study design of MR analysis. **(A)** The whole workflow of MR analysis. **(B)** Directed acyclic graph of instrumental variable assumptions made in univariable Mendelian randomization. **(C)** Directed acyclic graph of instrumental variable assumptions made in multivariable Mendelian randomization. **(D)** Directed acyclic graph of instrumental variable assumptions made in Two-step Mendelian randomization. CRC, colorectal cancer; MR, Mendelian randomization; MR-BMA, two-sample multivariate MR method based on Bayesian model averaging; Netmoss, Network module structure shift; SNP, single nucleotide polymorphism.

Our study used a two-step MR method to assess the mediating role of gut microbial traits in dietary habits affecting CRC at different cancer subsites and stages using data from 14 dietary habits associated with colorectal cancer and 9 microbial markers that were considered to have robust causal relationship with outcome. First, a two-sample MR analysis was used to assess the total effect of dietary habits on the six disease phenotypes. IVs for dietary habits are subsequently used to estimate the causal effect of exposure on potential mediators. If there is evidence that dietary habits affect the intestinal microbiota and that this dietary habit has an impact on the risk of CRC development, we used the “coefficient product” method to estimate the indirect effect of dietary habits on outcomes through gut microbial traits (34), that is, the causal effect value of dietary habits on each microbial trait is multiplied by the causal effect value of each microbial trait on the outcome, so as to obtain the mediating effect of each gut microbial trait. In addition, the indirect effect was divided by the total effect of dietary habits on the outcome to obtain the proportion mediated by each indirect factor. **Figure 1D** outlines this approach. Finally, the delta method is used to obtain the standard error of indirect effects (35). The study design flow is shown in **Figure 1**.

This study used TwoSampleMR, MendelianRandomization, MRPRESSO, ieugwasr, and NetMoss R packages, as well as the GitHub repository for MR-BMA https://github.com/verena-zuber/, and in R (version 4.0.5) for analysis, followed by retrieval of secondary trait associations using Phenoscanner.

## 3 Results

Comprehensively evaluating univariate MR, potential biomarkers ranking analysis, and microbial risk factors identification analysis based on the MR-BMA and the NetMoss method, we found 9 causal microbial markers for CRC at different cancer subsites and stages (**Table 2**). In addition, mediation analysis identified 10 pathways by which diet regulates gut bacteria to influence disease risk (**Table 3**).

**Table 2:** Summary of comprehensive analysis results.

Exposure	Outcome	Univariable MR^7^	Potential risk factors ranking (MIP^8^ > 0.1)	Microbial risk factors identifying (Top 10)	NetMoss analysis	Specificity^9^ (Y/N)
*Streptococcus thermophilus*	CRC^1^	0.80 (0.69,0.94), *P* = 0.005	MIP = 0.56	MIP = 0.360	Score < 1	Y
Erysipelotrichaceae	CRC	0.84 (0.73,0.97), *P* = 0.015	MIP < 0.1	MIP = 0.138	Score = 1	N
*Eubacterium ventriosum*	CC^2^	0.78 (0.68,0.89), *P* = 0.0003	MIP < 0.17	MIP = 0.219	Score = 1	Y
*Streptococcus*	CC	0.84 (0.77,0.93), *P* = 0.0003	MIP < 0.1	MIP = 0.118	Score = 1	N
Erysipelotrichaceae	CR^3^	0.80 (0.69,0.94), *P* = 0.005	MIP = 0.17	MIP = 0.284	Score = 1	Y
*Coprococcus sp_ART55_1*	CR	0.75 (0.59,0.96), *P* = 0.021	MIP = 0.45	MIP = 0.103	Score < 1	Y
*Eubacterium siraeum*	CR	0.72 (0.53,0.97), *P* = 0.033	MIP > 0.1	MIP = 0.105	Score = 1	Y
*Bacteroides ovatus*	BCR^4^	1.12 (1.01,1.23), *P* = 0.03	MIP = 0.23	MIP = 0.178	Score = 1	Y
*Bifidobacterium adolescentis*	BCR	1.67 (1.09,2.31), *P* = 0.04	MIP = 0.34	MIP = 0.043	Score < 1	Y
*Bacteroides ovatus*	BC^5^	1.12 (1.01,1.25), *P* = 0.04	MIP = 0.81	MIP = 0.032	Score = 1	Y
*Bacteroides ovatus*	BR^6^	1.17 (1.02,1.35), *P* = 0.024	MIP < 0.1	MIP = 0.136	Score = 1	Y
*Sutterellaceae unclassified*	BR	0.79 (0.64,0.97), *P* = 0.024	MIP = 0.15		Score = 1	N

^1^ colorectal cancer; ^2^ malignant tumors of colon; ^3^ malignant tumors of rectum; ^4^ benign tumors of colorectum; ^5^ benign tumors of colon; ^6^ benign tumors of rectum; ^7^ Mendelian randomization; ^8^ marginal inclusion probability; ^9^ there is no significant causal relationship between bacteria and six other non-colorectal cancer diseases.

**Table 3:** The results of mediation analysis.

Pathway	Exposure-Outcome	Exposure-Mediator	Mediator-Outcome		Two-step MR	
	b1^7^	b2^8^	b3^9^	b^10^	p^11^	Mediated Proportion (%)
Alcohol drinker status: Current-*Eubacterium ventriosum*-CC^2^	11.03	-19.24	-0.25	4.75	0.00	43.04
Bread intake-*Bacteroides ovatus*-BCR^4^	0.54	0.63	0.11	0.07	0.04	12.64
Cereal type: Biscuit cereal (e.g. Weetabix)-Erysipelotrichaceae-CRC^1^	-2.99	2.44	-0.17	-0.42	0.94	13.97
Ferritin-*Streptococcus thermophilus*-CRC	0.27	-0.42	-0.22	0.09	0.02	34.22
Ferritin-*Sutterellaceae unclassified*-BR^6^	0.35	0.72	-0.24	-0.18	0.97	-49.67
Lamb/mutton intake-*Eubacterium siraeum*-CR^3^	-2.19	-0.62	-0.33	0.21	0.07	-9.51
Liver intake-*Eubacterium ventriosum*-CC	0.84	8.90	-0.25	-2.20	0.97	-261.5
Milk type used: Never/rarely have milk-*Bifidobacterium adolescentis*-BCR	5.39	51.02	0.46	23.45	0.01	435.4
Milk type used: Never/rarely have milk-*Streptococcus*-CC	9.33	26.29	-0.17	-4.46	0.99	-47.79
Mineral and other dietary supplements: Calcium-*Bacteroides ovatus*-BR	4.05	2.99	0.16	0.48	0.05	11.85
Mineral and other dietary supplements: Calcium-*Bacteroides ovatus*-BCR	2.86	2.99	0.11	0.33	0.05	11.40
Mineral and other dietary supplements: Calcium-*Bacteroides ovatus*-BC^5^	4.69	2.99	0.11	0.34	0.06	7.23
Mineral and other dietary supplements: Glucosamine-*Sutterellaceae unclassified*-BR	2.66	3.42	-0.24	-0.83	0.99	-31.11
Mineral and other dietary supplements: Zinc-*Bifidobacterium adolescentis*-BCR	3.86	-8.69	0.46	-3.99	0.99	-103.5
Single crust pastry intake-*Coprococcus sp_ART55_1*-CR	5.57	6.66	-0.29	-1.93	0.94	-34.57
Type of special diet followed: Gluten-free-Erysipelotrichaceae-CR	-6.55	-4.71	-0.22	1.05	0.05	-16.02
Type of special diet followed: Gluten-free-*Streptococcus*-CC	5.39	6.27	-0.17	-1.06	0.96	-19.74
Vitamin and mineral supplements: Multivitamins +/- minerals-*Bacteroides ovatus*-BC	2.62	2.03	0.11	0.23	0.04	8.82
Vitamin and mineral supplements: Multivitamins +/- minerals-*Bacteroides ovatus*-BCR	3.16	2.03	0.11	0.22	0.03	7.03
Vitamin and mineral supplements: Vitamin C–*Coprococcus sp_ART55_1*-CR	31.68	32.35	-0.29	-9.36	0.95	-29.54

^1^ colorectal cancer; ^2^ malignant tumors of colon; ^3^ malignant tumors of rectum; ^4^ benign tumors of colorectum; ^5^ benign tumors of colon; ^6^ benign tumors of rectum; ^7^ total effect of exposure on outcomes; ^8^ effect of exposure on mediator; ^9^ effect of mediator on outcomes; ^10^ the mediator effect of exposure regulatory mediations to affect outcomes, ^10^ = b2*b3; ^11^ = b/b1 the probability of the mediation effect.

### 3.1 Univariable MR

After excluding SNPs that did not meet the criteria for IVs, 3310 SNPs were strongly associated with 412 microbial traits (**Table S3**). To assess the strength of the IV, the *F* value of each SNP is calculated, and the *F* statistic of the IV is between 408 and 797, both greater than 10, indicating that there is no weak tool variable bias (**Table S4**). Using Phenoscanner query, rs2450114 was found to be closely related to BCR and BR (*P* < 1×10^-5^), so the SNP was deleted and the MR analysis continued.

**Figure 2:**
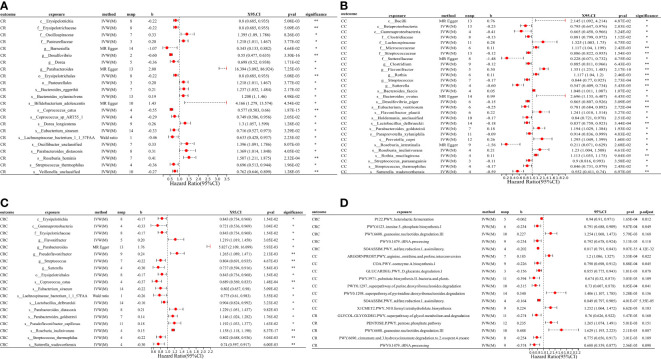
MR forest plots of the causal effects of microbial traits on malignant tumors. **(A)** MR forest plot of the causal effects of microbial traits on malignant tumors of rectal. **(B)** MR forest plot of the causal effects of microbial traits on malignant tumors of colon. **(C)** MR forest plot of the causal effects of microbial traits on colorectal cancer. **(D)** MR forest plot of the causal effects of microbial functional pathways on malignant tumors. CRC, colorectal cancer; CC, malignant tumors of colon; CR, malignant tumors of rectal; IVW(M), Inverse variance weighted (multiplicative random effects); nsnp, the number of IVs; 95%CI or X95.CI, OR (95% confidence interval); significance, Whether the P-value of the false discovery rate (*FDR*) correction is less than 0.2 (< 0.2**, > 0.2*); p.adjust, the *P*-value corrected for the false discovery rate (*FDR*) was derived using the Benjamini-Hochberg (*BH*) method.

**Figure 3:**
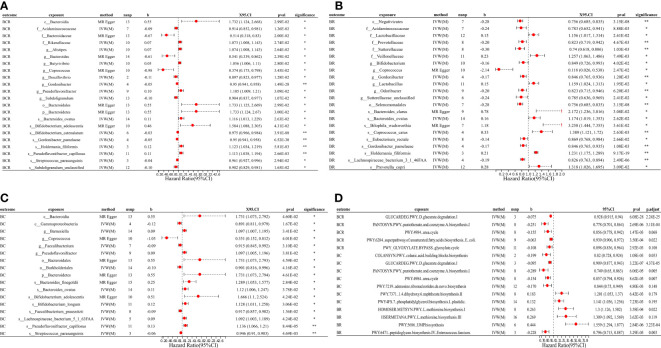
MR forest plots of the causal effects of microbial traits on benign tumors. **(A)** MR forest plot of the causal effects of microbial traits on benign tumors of colorectum. **(B)** MR forest plot of the causal effects of microbial traits on benign tumors of rectal. **(C)** MR forest plot of the causal effects of microbial traits on benign tumors of colon. **(D)** MR forest plot of the causal effects of microbial functional pathways on benign tumors. BCR, benign tumors of colorectum; BC, benign tumors of colon; BR, benign tumors of rectal; IVW(M), Inverse variance weighted (multiplicative random effects); nsnp, the number of IVs; 95%CI or X95.CI, OR (95% confidence interval); significance, Whether the P-value of the false discovery rate (FDR) correction is less than 0.2 (< 0.2**, > 0.2*); p.adjust, the *P*-value corrected for the false discovery rate (*FDR*) was derived using the Benjamini-Hochberg (*BH*) method.

Using the IVW(M), Wald ratio, and MR Egger methods, we found a potential causal relationship between 40 microbial traits and CRC, 56 microbial traits and CC, 43 microbial traits and CR, 45 microbial traits and BCR, 40 microbial traits and BC, and 43 microbial traits and BR (*P* < 0.05), of which a significant causal relationship between 12 microbial traits and CRC, 20 microbial traits and CC, 12 microbial traits and CR, 10 microbial traits and BCR, 9 microbial traits and BC, and 14 microbial traits and BR were found (IVW(M) MR, *P_fdr_* < 0.2). And there are differences in gut microbial biomarkers in the colon and rectum. Details can be found in **Figures 2**, **3**.

For causal relationships between microbial traits and malignant tumors, IVVW (M) analysis showed that *Streptococcus* was a significant protective factor for CRC (OR = 0.804, 95%CI = 0.691 ~ 0.935, *P* = 0.005) and CC (OR = 0.844, 95%CI = 0.770 ~ 0.925, *P* = 0.0003). In addition, *Streptococcus thermophilus* (OR = 0.802, 95%CI = 0.688 ~ 0.936, *P* = 0.005) and Erysipelotrichaceae (OR = 0.843, 95%CI = 0.734 ~ 0.968, *P* = 0.015) were found to have a negative causal relationship with CRC after *FDR* correction. *Eubacterium ventriosum* (OR = 0.781, 95%CI = 0.684 ~ 0.892, *P* = 0.0003) was a protective factor of CC; Erysipelotrichaceae (OR = 0.800, 95%CI = 0.685 ~ 0.935, *P* = 0.005), *Coprococcus sp_ART55_1* (OR = 0.749, 95%CI = 0.586 ~ 0.956, *P* = 0.021) and *Eubacterium siraeum* (OR = 0.716, 95%CI = 0.527 ~ 0.973, *P* = 0.033) had a potential protective effect on CR.

Results suggested a causal relationship between gut microbial traits and benign tumors. *Bacteroides ovatus* showed a suggestive causal association with BCR (OR = 1.12, 95%CI = 1.01 ~ 1.23, *P* = 0.03), BC (OR = 1.12, 95%CI = 1.01 ~ 1.25, *P* = 0.04), and BR (OR = 1.17, 95%CI = 1.02 ~ 1.35, *P* = 0.03). *Sutterellaceae unclassified* (OR = 0.785, 95%CI = 0.636 ~ 0.969, *P* = 0.024) was causally associated with BR. MR Egger analysis suggested that *Bifidobacterium adolescentis* was a potentially dangerous microorganism for BCR (OR = 1.666, 95%CI: 1.100 ~ 2.524, *P* = 0.043) and BC (OR = 1.584, 95% CI = 1.088 ~ 2.305, *P* = 0.042).

Cochran’s Q statistic showed that only GLYCOCAT PWY: glycogen degradation I: bacterial. was heterogeneity for CC (Q = 23.412, Q_pval = 0.005). After removing SNPs showing horizontal pleiotropy in MR-PROSSO analysis, there was no heterogeneity in the IVs (Q_pval > 0.05) (**Table S5**). Although there are potential biomarkers (e.g., *Bifidobacterium adolescentis* and Sutterellaceae, etc.) that show no correlation in the results of IVW(M), MR-Egger analysis showed a level of pleiotropism between genetic variants of these biomarkers, and the results of MR-Egger and MR-PROSSO showed a potential causal relationship with the outcome. In addition, MR-Egger analysis showed that there was no horizontal pleiotropy (*P* > 0.05) for other microbial traits (**Table S5**). MR-PRESSO results showed significant levels of pleiotropy between Sutterellaceae and CRC (*P* = 0.006, pleiotropic SNPs: rs2004833, rs28517505, and rs59033852), GLYCOCAT PWY: glycogen degradation I: bacterial. and CC (*P* = 0.009, multiplex SNPs: rs115001375 and rs59657730), *Bilophila wadsworthia* and BCR (*P* = 0.035, pleiotropic SNP: rs10276776), *Bilophila wadsworthia* and BR (*P* = 0.007, pleiotropic SNP: rs10276776), *Eubacterium hallii* and BR (*P* = 0.017, pleiotropic SNP: rs1330325). After removing the outliers, the results changed greatly, there was no causal effect between Sutterellaceae and CRC (OR = 0.85, 95% CI = 0.66 ~ 1.10, *P* = 0.23); *Bilophila wadsworthia* and CRC (OR = 0.91, 95% CI = 0.79 ~ 1.06, *P* = 0.243), and *Eubacterium hallii* and BR (OR = 1.056, 95% CI = 0.854 ~ 1.306, *P* = 0.616). GLYCOCAT PWY: glycogen degradation I: bacterial. and *Bilophila wadsworthia* exhibit potential causal relationships with CC (OR = 0.929, 95% CI = 0.551 ~ 0.966, *P* = 0.028, Q_pval = 0.229) and BR (OR = 0.712, 95% CI = 0.515 ~ 0.984, *P* = 0.040, Q_pval = 0.324), respectively. MR-PROSSO results for other microbial traits showed no horizontal pleiotropy (*P* > 0.05) or MR-PROSSO could not identify pleiotropic SNPs. Details are provided in **Table S5**. What’s more, MR Steiger analysis showed a forward causal direction from exposure to outcome (all *P* < 7×10^-5^, **Table S6**), and colocation analysis found that the variation in exposure and outcome was not attributable to the same underlying genetic variation (based on PP.H4.abf < 0.8, **Table S7**), suggesting that the causal regression returns unbiased estimates for the causal effect.

We used a two-sample univariate MR of these microbial traits with six non-CRC diseases to explore whether these bacteria are specific biomarkers for colorectal cancer. The results found that *Sutterellaceae unclassified* was also a significant protective microorganism for non-alcoholic fatty liver disease (OR = 0.811, 95% CI = 0.705 ~ 0.934, *P* = 0.004), and Erysipelotrichaceae significantly reduced the risk of irritable bowel syndrome (OR = 0.897, 95% CI = 0.844 ~ 0.954, *P* = 0.0006).

### 3.2 Potential biomarkers ranking

We used MR-BMA to rank the microbial biomarkers that were nominally significantly associated with outcome in the MR according to their MIP > 0.1. For malignant tumors: the first three biomarkers of CRC were *Pseudoflavonifractor* (MIP = 0.91, MACE = 0.26), *Streptococcus thermophilus* (MIP = 0.56, MACE = -0.11), and *Coprococcus catus* (MIP = 0.43, MACE = -0.11) (**Table S8**). The biomarkers associated with CC were Gammaproteobacteria (MIP = 0.20, MACE = -0.03), *Eubacterium ventriosum* (MIP = 0.17, MACE = -0.03), *Holdemania unclassified* (MIP = 0.15, MACE = -0.02), and *Sutterella wadsworthensis* (MIP = 0.14, MACE = -0.02) (**Table S8**). The biomarkers associated with CR were *Coprococcus sp_ART55_1* (MIP = 0.45, MACE = -0.09), *Desulfovibrio* (MIP = 0.36, MACE = -0.11), and Erysipelotrichaceae (MIP = 0.17, MACE = -0.04) (**Table S8**).

For benign tumors, risk factors for BCR were Bacteroidales (MIP = 0.43, MACE = 0.063), *Bifidobacterium adolescentis* (MIP = 0.34, MACE = -0.05), *Bacteroides ovatus* (MIP = 0.23, MACE = 0.03), and *Subdoligranulum unclassified* (MIP = 0.14, MACE = -0.02) (**Table S9**). Risk factors for BC were only *Bacteroides ovatus* (MIP = 0.81, MACE = 0.12) (**Table S9**). There were three risk factors for BR: Acidaminococcaceae (MIP = 0.82, MACE = -0.17), *Eubacterium rectale* (MIP = 0.35, MACE = -0.09) and *Sutterellaceae unclassified* (MIP = 0.15, MACE = -0.02) (**Table S9**). MIP < 0.1 for all other risk factors (**Tables S8**, **S9**). The MACE directions for these biomarkers also exhibited consistency with our MR results. In the preliminary analysis of CC and CR, the detection of the influence point highlighted rs12736307 and rs76321722, respectively, which had a greater impact on the analysis (**Figure S2**). The genetic variation in the remaining results was not consistent with the large *q*-statistic or Cook distance (**Figure S3**). Additional details can be found in the **Supplementary Material**.

### 3.3 Microbial risk factors identification

In this section, we selected the top 10 microorganisms in terms of MIP as the “true” causal risk factors for the outcome. Among them, the results of MIP greater than 0.2 and cross-validation with other analysis results are as follows: For malignancy (**Table S10**), *Streptococcus thermophilus* (MIP = 0.36, MACE = -0.037) and Erysipelotrichaceae (MIP = 0.138, MACE = -0.018) were risk factors for CRC. The top risk microbial traits in CC were: *Eubacterium ventriosum* (MIP = 0.219, MACE = -0.029) and *Streptococcus* (MIP = 0.118, MACE = -0.017). Risk factors for CR included Erysipelotrichaceae (MIP = 0.284, MACE = -0.064), *Eubacterium siraeum* (MIP = 0.106, MACE = -0.021), and *Coprococcus sp_ART55_1* (MIP = 0.103, MACE = -0.011). For benign tumors (**Table S11**), risk factors for BCR included *Bacteroides ovatus* (MIP = 0.178, MACE = -0.017) and *Bifidobacterium adolescentis* (MIP = 0.043, MACE = -0.003). And the risk factors associated with BC (MIP = 0.032, MACE = 0.002) and BR (MIP = 0.136, MACE = 0.022) had *Bacteroides ovatus*. The results of the microbial risk factors identification, the detection of the impact point of BR highlights rs9884588, which had a greater impact on the analysis (**Figure S4**). None of the genetic variants in the remaining results had a large *q*-statistic or consistent *Cd* (**Figures S4**, **S5**), and there were no outliers or influential points that needed to be removed. Other outcomes are presented in the **Supplementary Material**.

### 3.4 NetMoss analysis

**Figure 4:**
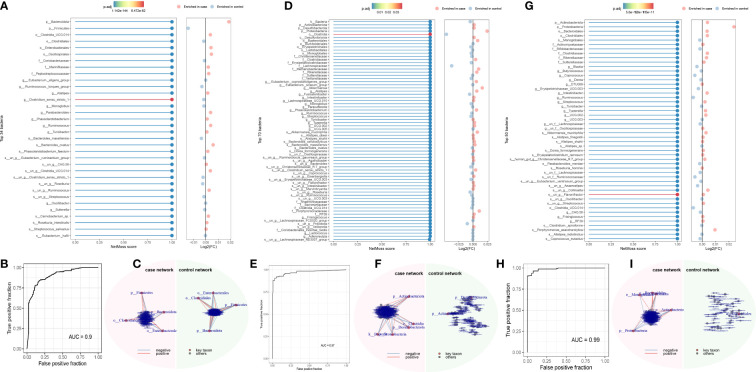
NetMoss results plot. **(A)** NetMoss2 identifies specific bacterial taxa in microbial Sparcc networks between adenomas and healthy people; **(B)** NetMoss2-constructed ROC plots of adenomas and healthy people; **(C)** Sparcc network diagram between adenomas and healthy people constructed by NetMoss2; **(D)** NetMoss2 identifies specific bacterial taxa in microbial Sparcc networks between colorectal cancer and healthy people; **(E)** NetMoss2-constructed ROC curves for colorectal cancer and healthy people; **(F)** Sparcc network diagram between colorectal cancer and healthy people constructed by NetMoss2; **(G)** NetMoss2 identifies specific bacterial taxa in the microbial Sparcc network between colorectal cancer and adenoma populations; **(H)** NetMoss2-constructed ROC curves of colorectal cancer and adenoma populations; **(I)** Sparcc network diagram between colorectal cancer and adenoma populations constructed by NetMoss2. .

Using the NetMoss method, we found that *Bacteroides ovatus* and *Sutterellaceae unclassified* were biomarkers that distinguish adenomas from healthy people (**Figure 4A**). Erysipelotrichaceae, Sutterellaceae, *Streptococcus*, *Bacteroides ovatus*, and *Eubacterium siraeum* were biomarkers that distinguish CRC from healthy people (**Figure 4D**). And these biomarkers in our above method suggest a causal relationship with the development of CRC. Meanwile, we found some microorganisms that did not duplicate the results of MR (**Figures 4A–I**). Detailed results can be found in the **Supplementary Material**.

### 3.5 Mediation analysis

**Figure 5:**
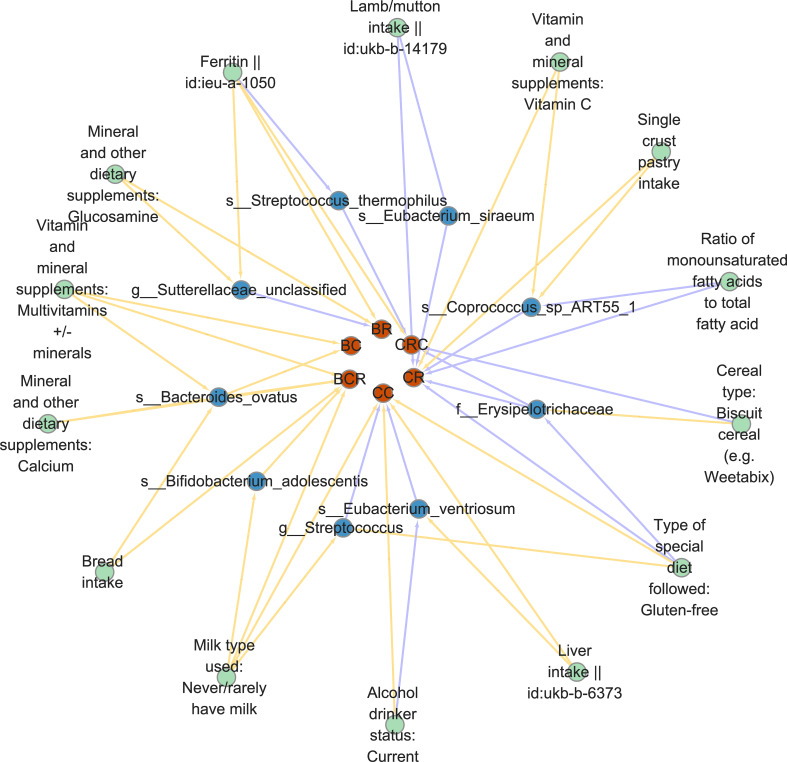
Diet-gut bacteria-colorectal cancer pathway diagram in mediated analysis.

Since dietary habits are essential for the prevention and management of CRC, and the intestinal microbiota may be a mediator of the influence of dietary habits on CRC at different cancer subsites and stages. Through the “coefficient product” method, we identified a total of 20 causal pathways in which dietary habits regulate gut bacteria and thus affect the occurrence and development of CRC (**Figure 5** and **Table 3**), of which the mediating direction of 10 causal pathways is consistent with the direction of dietary habit-outcome. Including: The effect of current drinking status on CC was partially mediated by *Eubacterium ventriosum* (indirect effects (β) = 4.748, *P* = 0.002, mediated proportion: 43.044%); Cereal type: biscuit cereals (e.g. Vita) might reduce the risk of CRC by reducing Erysipelotrichaceae abundance (β = -0.417, *P* = 0.941, mediated proportion: 13.973%); Excessive ferritin intake led to a decrease in the abundance of *Streptococcus thermophilus*, which in turn resulted in an increased risk of CRC (β = 0.09, *P* = 0.02, mediated proportion: 34.22%); *Bacteroides ovatus* mediated the effects of minerals and other dietary supplements: calcium (β = 0.327, *P* = 0.053, mediated proportion: 11.854%) and bread intake (β = 0.068, *P* = 0.041, mediated proportion: 12.639%). Finally, the indirect effect of never/rarely drinking milk on BCR was estimated by *Bifidobacterium adolescentis*, and it was found that the mediating effect of *Bifidobacterium adolescentis* was 23.453, and the mediated proportion was 435.399%.

## 4 Discussion

CRC has a high mortality rate when detected at an advanced stage, so understanding the causes of CRC at different cancer subsites and stages and identifying its risk factors are important for early screening and prevention of CRC. In this study, a variety of methods were used to systematically investigate microbial biomarkers of CRC at different cancer subsites and stages in European populations. The results showed that in the MR-based univariate, potential biomarkers ranking, microbial risk factors identification, and NetMoss method, 3 or 4 methods consistently found that 9 bacteria were closely related to the development of CRC. In the mediation analysis of diet-gut microbiota-disease, 10 diet-gut bacteria-CRC causal pathways were found.

Although two MR studies have examined the causal relationship between gut flora and CRC (36, 37), compared to these two MR analyses, the present study more comprehensively examined the causal relationship between gut microbes and CRC, and multiple validation analyses and extended analyses were performed, including: 1) Instead of simply exploring the causal relationship between gut flora and CRC, this study also examined the relationship between gut flora and CRC at different cancer subsites and stages; 2) the use of univariate MR and MR-BMA analyses to identify and rank causal microbial markers of CRC at different cancer subsites and stages, and the use of multi-population cohort data for validation on the other hand; 3) after finding robust causal microbes, this study investigates dietary habits that influence these microbes, providing theoretical support for the dietary habits-gut flora-colorectal cancer pathway; 4) this study conducts corresponding analyses on smaller bacterial taxa species.

A large number of studies have reported the correlation between gut microbiology and CRC, and intestinal microbiota is believed to be directly involved in CRC (6, 9, 10, 38, 39). A multi-cohort study found that *Streptococcus thermophilus* was dramatically reduced in stool samples from patients with CRC (10). *Streptococcus thermophilu* and a commercial probiotic, *Lactobacillus rhamnosus* GG, have similar *in vitro* probiotic properties as well as anticancer activity and folate production (40). Intriguingly, a recent experiment combining cells with mice further found that *Streptococcus thermophilus* was a novel preventive measure for CRC prevention in mice (39). *Streptococcus thermophilus* could secrete β-galactosidase to inhibit cell proliferation, reduce colony formation, induce cell cycle arrest, promote apoptosis of cultured CRC cells, and delay the growth of CRC xenografts. And *Streptococcus thermophilus* can increase the gut abundance of known probiotics, including Bifidobacterium and Lactobacillus, through β-galactosidase (39). These conclusions are consistent with our findings that *Streptococcus thermophilus* is a potential probiotic for CRC. This could further demonstrate the robustness of our results.

This study also found that *Eubacterium ventriosum* and *Streptococcus* are CC protecting microorganisms, and Erysipelotrichaceae, *Coprococcus sp_ART55_1* and *Eubacterium siraeum* are protective factors for CR. Previous studies have found that the abundance of *Streptococcus* and Erysipelotrichaceae is significantly higher in adjacent tissues than in tumors (38, 41), and that abundance in the oral cavity is associated with a reduced risk of CRC (42). But there are also studies showing that *Streptococcus* and Erysipelotrichaceae are more common in patients with advanced colorectal adenomas or CRC (43, 44). Erysipelotrichaceae was significantly higher in the tumor group of 1,2-dimethylhydrazine-induced colon cancer animal models (45). Butyrate inhibits the development of CRC, and a significant decrease in butyrate-producing bacteria in the intestine, including *Eubacterium* (*Eubacterium siraeum* and *Eubacterium ventriosum*), is generally observed in CRC patients (46). The abundance of *Eubacterium* spp. was lower in the advanced colorectal adenoma group than in the healthy control group (44). At present, there are relatively few studies on these bacteria and colorectal cancer proposed in this study, and more studies and experiments are needed to verify these results and explore the specific mechanisms.

Understanding the biology of colorectal adenomas can lead to new strategies to screen for and reduce or slow the progression of these CRC precursors (4). Previous studies have found that *Bacteroides* in familial adenoma polyp mice are enriched compared to wild-type mice (47). *Bacteroides* are enriched in the intestinal type of patients with adenomas, and *Bacteroides ovatus* increases significantly when progressing from advanced adenomas to cancer (6). The accumulation of *Bacteroides ovatus* in peripheral blood drives the proliferation of CD27-MAIT cells that produce IL-17, a pro-inflammatory factor (48). But some studies have also shown that *Bacteroides ovatus* is a probiotic. *Bacteroides ovatus* promotes IL-22 production and reduces trinitrobenzenesulfonic acid-driven colonic inflammation (49). Since *Bacteroides ovatus* has the capacity to reestablish the equilibrium between lymphocytes and macrophages, its absence could throw the body’s natural immune system off balance, cause inflammation, and lead to the death of intestinal epithelial cells (50). The results of our study suggest that *Bacteroides ovatus* is a new risk biomarker for early CRC, and since this relationship has not been fully established in prior studies, more research is required to validate and advance early CRC prevention and treatment.

Diet is one of the most important and modifiable variables influencing the gut microbiome. Our findings and those of earlier research indicate that regular alcohol use alters the gut microbiota, which raises the chance of colon cancer. First, research on both humans and animals have demonstrated that long-term ethanol consumption causes dysbiosis, which lowers the abundance of Firmicutes and Bacteroidetes while increasing the abundance of butyrate-producing taxa in Clostridiales (51). In chronic alcoholism, the number of anaerobic bacteria decreases and the number of Streptococcus increases (52, 53). Intriguingly, our research revealed that alcohol consumption decreases the amount of the anaerobic bacterium *Eubacterium ventriosum*, which raises the risk of colon cancer, and that *Coprococcus* and *Eubacterium ventriosum* can work together to create more butyric acid. This is in line with the findings of earlier studies, which suggest that alcohol consumption regulates a causal pathway in which *Eubacterium ventriosum* increases the risk of CRC and that further mechanistic studies are required to confirm this causal pathway.

In addition, there is strong evidence that consuming more dairy and milk reduces the risk of CRC (51). Other ingredients in dairy products also have antitumor activity, including conjugated linoleic acid, lactose, butyrate, and lactic acid-producing bacteria, and a recent intervention study in patients with irritable bowel syndrome, a pathology associated with inflammation and CRC, showed that consumption of fermented dairy products containing dairy starter cultures and *Bifidobacterium animalis* enhanced SCFA production and reduced the abundance of *Bilophila wadsworthia*, The influence of microorganisms and other compounds in dairy products on the composition and function of gut microbes has been shown (54). Our study came to a similar conclusion: never drinking milk increases the abundance of *Bifidobacterium adolescentis*, which leads to an increased probability of BCR.

*Bacteroides* is an obligate or strictly Gram-negative anaerobic bacteria, and its composition and metabolic activity are largely regulated by diet. *Bacteroides* are associated with high fat and protein intake. Xylan-regulated human keratinocyte growth factor-2 is delivered to the inflammatory colon *via Bacteroides ovatus* (55). Our results also found three dietary factors: Vitamin and mineral supplements: multivitamin +/- minerals, minerals and other dietary supplements: Calcium and bread intake can increase the risk of BCR by regulating the abundance of *Bacteroides ovatus.*

This study has several advantages. 1) A comprehensive assessment of the causal relationship between gut microbial traits and CRC at different cancer subsites and stages, and the results of multiple methods consistently demonstrate the robustness of our findings. Most of our results support the findings of previous studies that served as positive controls for our approach. Our study allowed to generate hypotheses proposing some biomarkers for which there was little previous evidence of causal association, such as *Eubacterium ventriosum* and *Bacteroides ovatus*, among others. 2) To further elucidate how some dietary factors may influence CRC risk in different cancer subsites and stages by modulating the gut microbiota. 3) Because microbial traits show strong correlations both phenotypically and genetically, MVMR analysis may be more appropriate to assess the causal relationship between microbes and phenotype. However, because microbial trait data are high-throughput, and traditional MVMR methods are designed for a small number of risk factors, they cannot be extended to high-throughput dimensions. Therefore, this study is the first of its kind to use the MR-BMA method to go for causal ranking of microbial markers to select the truly likely risk factors from a large number of candidate risk factors, which in turn enhances the robustness of our findings.MR-BMA (a method capable of explaining the multi-effectiveness of measurements) largely confirms univariate findings. Among other things, the MR-BMA approach proposes multivariate models of combinations of microorganisms that can be used to evaluate the role of microbial combinations for disease and applied to the early screening of benign tumors.

The present study also has some limitations. 1) GWAS of intestinal flora is still in its infancy in terms of sample size, the population samples of intestinal flora we use are not large enough and the loci identified so far are still very limited. 2) The threshold for our screening gut microbial instrumental variables was set at *P* < 1 × 10^-5^, and although steps have been taken to ensure by calculating the *F* statistic for each instrument validity of the SNP, we cannot exclude the possibility of false negative errors due to insufficient statistical efficacy. The efficacy of IVs is also a significant drawback in MR-BMA analysis. 3) Our understanding of the microbiomes of different cancer subsites (e.g. colon, rectum or colorectum) is still limited, and easily accessible fecal material may reflect a suitable substitute for colorectal microbiota, however, there may be errors in going from fecal microbes to causal inquiry, but this is a limitation of current data, and hopefully more studies will be available in the future to fill this gap.

In conclusion, this study conducted a comprehensive exploratory MR study that identified 6 protective bacteria for malignancies, 2 risk bacteria and a protective bacterium for benign tumors. The findings support the hypothesis that the gut microbiota is the etiology of CRC and that the effects on CRC are different for different cancer subsites and stages, suggesting that microorganisms are specific for the prevention, treatment, and improvement of CRC. Among them, the protective effect of *Streptococcus thermophilus* on CRC has been verified by cellular and animal experiments. At the same time, the results of the mediation analysis provide both theoretical support and empirical evidence for modifying dietary habits to regulate gut bacteria and thus influence CRC at different cancer subsites and stages, suggesting that controlling gut flora may be a promising strategy for colorectal cancer prevention in specific dietary populations. In addition, these findings can provide ideas and directions for further mechanistic studies such as animal models or biomarker-based human trials to help guide the development and clinical translation of potential microbiota-based cancer prevention strategies.

## Data availability statement

The original contributions presented in the study are included in the article/**Supplementary Material**. Further inquiries can be directed to the corresponding authors.

## Author contributions

LZ and GZ designed the research. HL and DS collected the data and analyzed it. HL and CJ performed the literature search and drafted the article. LZ and GZ supervised the study. All authors contributed to the article and approved the submitted version.
