# Supplementary material for: Identifying and ranking causal microbial biomarkers for colorectal cancer at different cancer subsites and stages: a Mendelian randomization study
Source: Front Oncol. 2023 Jul 19;13:1224705. doi: 10.3389/fonc.2023.1224705 (PMC10395834; doi:10.3389/fonc.2023.1224705)
Supplement: Supplementary file 1 [file DataSheet_1.docx]

Supplementary Material

Identifying and ranking causal microbial biomarkers for colorectal cancer at different cancer subsites and stages: a Mendelian randomization study

Hongfeng Li ^1, 2†^, Dashuang Sheng ^1, 2†^, Chuandi Jin ^1, 2^, Guoping Zhao ^1, 2, 4, 5*^, Lei Zhang ^1, 2, 3, 4*^

^1^Department of Biostatistics, School of Public Health, Cheeloo College of Medicine, Shandong University, Jinan, China

^2^Microbiome-X, National Institute of Health Data Science of China, Cheeloo College of Medicine, Shandong University, Jinan, China

^3^Shandong Children’s Microbiome Center, Children’s Hospital affiliated to Shandong University, Jinan, China

^4^State Key Laboratory of Microbial Technology, Shandong University, Qingdao, China

^5^CAS Key Laboratory of Computational Biology, Bio-Med Big Data Center, Shanghai Institute of Nutrition and Health, University of Chinese Academy of Sciences, Chinese Academy of Sciences, Shanghai, China

**† These authors contributed equally to this work**

*** Correspondence:**

Lei Zhang, PhD,

Professor & PI,

Email: [zhanglei7@sdu.edu.cn](mailto:zhanglei7@sdu.edu.cn)

Guoping Zhao, PhD,

Professor & PI,

Email: [guopingzhao@yeah.net](mailto:guopingzhao@yeah.net)

**Materials**

1. **Additional description**

**1.1 Materials and Methods**

**1.2 Results**

**1.3 References**

1. **Supplementary Figures and Tables**

**2.1 Supplementary Figures**

**Figure S1.** Genetic correlations between gut bacteria.

**Figure S2.** Diagnostic plot of outliers and influential genetic variants in the underlying biomarker ranking method.

**Figure S3.** Diagnostic plot of outliers and influential genetic variants in the underlying biomarker ranking method.

**Figure S4.** Diagnostic plot of outliers and influential genetic variants in the microbial risk factor determination method.

**Figure S5.** Diagnostic plot of outliers and influential genetic variants in the underlying biomarker ranking method.

**Figure S6.** Genetic correlation heat map between malignancy-associated gut bacteria in potential biomarker sequencing methods.

**Figure S7.** Genetic correlation heat map between benign tumor-associated gut bacteria in potential biomarker sequencing methods.

**2.2 Supplementary Tables**

**Table S1.** Cohort data details for analysis

**Table S2.** Prior probability threshold for Q-statistics and Cd identification of anomalies and strong influence points in sensitivity analysis.

**Table S3.** Instrumental variables for gut microbial traits.

**Table S4.** *F* statistic of IVs in Mendelian randomization analysis.

**Table S5.** Results of sensitivity analysis in Mendelian randomization analysis.

**Table S6.** Steiger test results from exposure to outcome.

**Table S7.** Colocalization analysis where loci provide evidence of a shared causal variant.

**Table S8.** MR-BMA ranking of microbial biomarkers for malignant tumors based on MIP and MACE in potential biomarkers ranking analysis.

**Table S9.** MR-BMA ranking of microbial biomarkers for benign tumors based on MIP and MACE in potential biomarkers ranking analysis.

**Table S10.** MR-BMA ranking of microbial biomarkers for malignant tumors based on MIP and MACE in microbial risk factors identifying analysis.

**Table S11.** MR-BMA ranking of microbial biomarkers for benign tumors based on MIP and MACE in microbial risk factors identifying analysis.

**Table S12.** A detailed list of 194 microorganisms with strongly correlated independent genetic variation in microbial risk factors identifying analysis.

**Table S13.** In the analysis of potential biomarkers ranking, after excluding outliers and influential variants, the combined model (risk factor set) of colorectal cancer and BCR is ranked according to its posterior probability (pp).

**Table S14.** In the analysis of potential biomarkers ranking, after excluding outliers and influential variants, the combined model (risk factor set) of CC and BC is ranked according to its posterior probability (pp).

**Table S15.** In the analysis of potential biomarkers ranking, after excluding outliers and influential variants, the combined model (risk factor set) of CR and BR is ranked according to its posterior probability (pp).

**Table S16.** In the analysis of microbial risk factors identifying, after excluding outliers and influential variants, the combined model (risk factor set) of CRC and BCR is ranked according to its posterior probability (pp).

**Table S17.** In the analysis of microbial risk factors identifying, after excluding outliers and influential variants, the combined model (risk factor set) of CC and BC is ranked according to its posterior probability (pp).

**Table S18.** In the analysis of microbial risk factors identifying, after excluding outliers and influential variants, the combined model (risk factor set) of CR and BR is ranked according to its posterior probability (pp).

**Table S19.** Results of Mendelian randomization analysis of dietary habits with different niches and different periods of colorectal cancer.

**Table S20.** Results of a Mendelian randomization analysis of dietary habits and 8 gut bacteria.

# Additional description

**1.1 Materials and Methods**

**Data sources**

In this study, a multi-population cohort was constructed by collecting the human gut microbiota 16S rRNA dataset from the European colorectal cancer cohort from the National Center for Biotechnology Information (NCBI). Keywords we searched for in PubMed included "gut microbiota," "colorectal cancer," "16S," "human," "stool," and "microbiome." Only adult stool samples are retained for downstream analysis. In total, our study collected 16S data from 6 studies from European populations,(Zeller *et al.*, 2014; Baxter, Koumpouras, *et al.*, 2016; Baxter, Ruffin, *et al.*, 2016; Sze *et al.*, 2017; Hannigan *et al.*, 2018; Dadkhah *et al.*, 2019) including 318 colorectal cancer patient samples, 576 adenoma patient samples, and 469 healthy control samples. Details can be found in Table S1.

**Potential biomarkers ranking**

MR-BMA is a two-sample multivariate MR method that prioritizes and selects "true" causal risk factors from high-dimensional candidate risk factors in a Bayesian framework,(Zuber *et al.*, 2020) In multivariate MR, genetic variants that meet the following criteria are considered effective instrumental variables: 1) Correlation: The variant is strongly associated with at least one risk factor; 2) Commutability: The variation is independent of all risk factors - all confounding factors associated with the outcome; 3) Exclusion Limitation: This variant is independent of outcomes determined by risk factors and confounding factors. This means that it is not necessary that every genetic variant is strongly associated with all risk factors.(Zuber *et al.*, 2020)

After the initial analysis, *q*-statistics and Cook distance (*Cd*) were used to examine potential instrumental variable (IV) outliers in the analysis and strong influence points in the model (*pp* > 0.1). Any SNPs with *q*-statistic > 10 or *Cd* > the median of a central *F*-distribution with d and n−d degrees of freedom were flagged, and MR–BMA was repeated after these SNP(s) were removed. Exposure-Outcome associations remaining after the removal of potential outliers were considered to be more reliably associated with outcome. The robustness of the results was tested using the prior variances (σ = 0.3 and σ = 0.7) for the specified causal parameters. Full details of the MR-BMA method can be found elsewhere.(Zuber *et al.*, 2020)

**Microbial risk factors identifying**

The univariate MR method was used to detect causality between exposure and outcome, which assumed that the exposures were independent and therefore might 1) ignore situations where there might be a “population” relationship, and 2) disallow the elimination of the effects of associated exposures by removing non-independent signals. MR-BMA provides an alternative way to model multiple microbial traits together. In this way, subgroups of microorganisms that may co-act on the pathogenic pathways of colorectal cancer development can be identified, and the independent microorganisms can be appropriately sequenced according to their independent causal signals. Thus, this approach can unravel relevant microbial signatures to determine which microbes may be the "true" driving causal signals. MR-BMA can adequately handle "measurement pleiotropy" as well as traditional MVMR.(Zuber *et al.*, 2020) Exposures that meet two of the following criteria can be included in the analysis of candidate risk factors: 1) correlation: risk factors need to be strongly instrumentalized by at least one genetic variant as an instrumental variable; 2) No multicollinearity: The genetic association of any of the included risk factors cannot be explained linearly by the genetic association of any other risk factor or by a combination of genetic associations of multiple other exposures included in the analysis.

Therefore, in this study, MR-BMA was used to identify risk factors with a "true" causal relationship with the outcome from 207 gut microbial risk factor candidates. A risk factor may be the cause of the effect, but if its causal effect is mediated by another risk factor included in the analysis, it will not be selected in the MVMR approach. Select effective risk factors and instrumental variables for microbial risk factor determination analysis by following these steps: 1) select *P* < 1×10^-5^ and SNPs after eliminating linkage imbalance in the whole genome; 2) match these SNPs to a complete GWAS aggregated dataset of all risk factors and outcomes by chromosomes and locations; 3) screening of included risk factors and SNPs based on criteria that SNPs are associated with at least one risk factor and that risk factors require strong instrumentalization by at least one genetic variant as an instrumental variable; 4) The β coefficients of genetic variation and available risk factors and the standard error (se) of genetic association were extracted for subsequent analysis; 5) calculate the genetic correlation structure between microbial taxa and randomly exclude one of each pair of microorganisms with a correlation higher than 0.985; 6) screen according to the criteria of risk factors and effective instrumental variables to obtain a final dataset that can be used for MR-BMA analysis for MR-BMA analysis. This study prioritizes and ranks risk factors using the MIP of MR-BMA, where σ^2^ = 0.25 as the prior variance of the analysis.

We interpret the top 10 bacteria in MIP as the strongest "true" causal candidate of all the bacteria offered in the model. All independent genetic variants strongly associated with 192 microbial biomarkers (15 microorganisms did not meet the criteria or were not strongly associated with SNPs, 192 microbial details in Table S12) were included in the analysis (*N* = 432). Furthermore, each microbial biomarker combination (all single biomarkers, all biomarker pairs, all three biomarkers, etc.) is considered as a candidate model in MVMR analysis using weighted regression. Each candidate model is assigned a posterior probability (*pp*) that uses the goodness-of-fit measure of regression to represent the likelihood that the candidate model contains a true set of causal biomarkers. Other analysis processes are largely consistent with the methodology for ranking potential risk factors.

**NetMoss analysis**

The abundance of a particular bacterium cannot describe the full picture of an ecosystem, let alone the transition from health to disease. Network analysis has been widely used in a variety of biological systems, however, it is difficult to directly compare networks from different studies because the structure of the network is often associated with various factors, such as the size of the dataset, the choice of critical values, and the method of construction. The NetMoss method focuses on the shift of network modules as a way to assess the importance of bacteria between different states.(Xiao, Zhang and Zhao, 2022) It can effectively reduce batch effects and identify more powerful biomarkers that traditional abundance methods have overlooked. This study identifies more robust biomarkers by comparing the results of this section with those of the previous three methods.

To determine microbial biomarkers associated with disease states. We used the Quantitative Insights Into Microbial Ecology 2 (QIIME2) platform to perform sequencing quality screening and analysis of 16S rRNA data from the collected multi-population cohort datasets (v2022.11).(Bolyen *et al.*, 2019) The dataset for each cohort is processed independently to obtain a count table with characteristics ranging from phylum to genus. Abundance tables for taxonomic levels were combined and converted into relative abundance tables. Low-abundance ASV, whose relative abundance did not reach 0.1% in at least 10% of the sample, was excluded. Used the vegdist function to calculate the Bray-Curtis distance between samples and visualize it using principal coordinate analysis (PcoA).

**1.2 Results**

**Potential biomarkers ranking**

In univariate MR analysis, 19, 29, 23, 22, 17, and 22 gut microbial taxa were potentially causally related to CRC, CC, CR, BCR, BC, and BR. These genetically predicted gut microbial biomarkers, nominally associated with different outcomes, were ranked using MR-BMA-based potential biomarkers ranking with a prior probability of 0.1. Since this is a Bayesian method, the *P* value of the frequency is not available. After ensuring the presence of strongly correlated SNPs for each microbial trait and trimming microbial traits with genetic correlations greater than 0.985, the final dataset for MR-BMA analysis consisted of 14 (CRC), 23 (CC), 19 (CR), 15 (BCR), 13 (BC), and 19 (BR) microbial taxa with 87, 122, 124, 102, 100, and 157 genetic variants as potential biomarkers measured as instrumental variables, respectively. Microbial genetic correlations for all outcomes were shown in Figure S8 and Figure S9.

When evaluating the entire model with changes in the combination of gut bacteria, the 4 causal models with a posterior probability (*pp*) greater than 0.1 for CRC were *Pseudoflavonifractor* and *Streptococcus thermophilus* (*pp* = 0.223), *Pseudoflavonifractor* and *Coprococcus catus* (*pp* = 0.137), *Pseudoflavonifractor* (*pp* = 0.121), *Pseudoflavonifractor*, *Coprococcus catus*, and *Streptococcus thermophilus* (*pp* = 0.114). Causal models in CC where pp greater than 0.1 did not exist. In CR, there were *Coprococcus sp_ART55_1* (*pp* = 0.179) and Erysipelotrichacea (*pp* = 0.138) for causal models with *pp* greater than 0.1. There were 2 models with *pp* values greater than 0.1 in BCR: Bacteroidales (*pp* = 0.296) and *Bifidobacterium adolescentis* (*pp* = 0.183). BC existed only *Bacteroides ovatus* (*pp* = 0.623), and this model has a *pp* value greater than 0.1. Acidaminococcaceae (*pp* = 0.289), Acidaminococcaceae and *Eubacterium rectale* (*pp* = 0.132) were 2 models with *pp* greater than 0.1 in BR. Full analysis of the best models for the 6 outcomes is provided in Table S13-Table S15. The results using a prior variance of 0.3 and 0.7 are consistent with the results of a prior variance of 0.5, which tests the robustness of the results**.**

**Microbial risk factors identifying**

After initial analysis, 432 SNPs were associated with 194 potential risk factors for all outcomes. After pruning bacteria with genetic correlation greater than 0.985 (including removing bacteria without strongly correlated SNPs), 427 SNPs of 142 bacteria were used to determine microbial risk factors for all 6 outcomes (CRC, CC, CR, BCR, BC and BR), and the top 10 microbota with MIP were selected as "true" causal risk factors for the outcome.

In addition, the best model showed that there are 3 causal models with *pp* greater than 0.01 in CRC: *Streptococcus thermophilus* (*pp* = 0.017), *Pseudoflavonifractor capillosus* (*pp* = 0.014), and *Paraprevotella xylaniphila* (*pp* = 0.01). CC had 3 causal models with *pp* greater than 0.005: *Streptococcus thermophilus* and *Eubacterium ramulus* (*pp* = 0.01), *Streptococcus thermophilus*, *Pseudoflavonifractor capillosus* and *Eubacterium ramulus* (*pp* = 0.007), *Streptococcus thermophilus* (*pp* = 0.05). *Ruminococcus bromii* (*pp* = 0.007) and *Lachnospiraceae bacterium_8_1_57FAA* (*pp* = 0.006) were causal models in CR where their *pp* greater than 0.005. BCR had 3 causal models with *pp* greater than 0.02: *Holdemania unclassified* (*pp* = 0.101), *Ruminococcus bromii* and *Holdemania unclassified* (*pp* = 0.033), *Bacteroides ovatus*, and *Holdemania unclassified* (*pp* = 0.028). BC and BR had 5 (*Ruminococcus bromii* (*pp* = 0.057), *Holdemania unclassified* (*pp* = 0.02), *Ruminococcus bromii* and *Holdemania unclassified* (*pp* = 0.011), *Escherichia unclassified* (*pp* = 0.01), *Ruminococcus* (*pp* = 0.01)) and 1 (*Parabacteroides goldsteinii* (*pp* = 0.026)) causal model where *pp* is greater than 0.01. The complete results of all combinatorial models were shown in Table S16-Table S18.

**Netmoss analysis**

Diversity analysis and PCoA analysis at the genera and species levels of the six cohorts revealed that there were batch effects between cohorts and significant differences in Bray-cutis distances in healthy, adenomas, and colorectal cancer populations (Figure S2).

**Mediation analysis**

This study performed a two-step MR analysis to investigate whether gut microbial traits are mediators of dietary habits on colorectal cancer at different niches and at different times. Genetic IVs for 14 dietary habits were first used to estimate the causal effect of exposure on 6 outcomes. We identified 22 causal relationships between these 14 exposures and 6 outcomes, including finding that current drinking status was associated with an increased risk of CC (β = 11.029, se = 5.439, *P* = 0.23), cereal type: biscuit cereals (e.g. Vitamar) migth reduce the risk of CRC (OR = 0.050, 95% CI = 0.044 ~ 0.0583, *P* < 0.0001), ferritin was a risk factor for CRC (OR = 1.314, 95% CI = 1.289 ~ 1.340, *P* = 2.30×10^-168^) and BR (OR = 1.423, 95% CI = 1.262 ~ 1.604, *P* = 8.42×10^-9^); Vitamin and mineral supplements: Multivitamin +/- minerals could increase the risk of developing BC (OR = 13.718, 95% CI = 3.706 ~ 50.779, *P* = 8.79×10^-5^), BCR (OR = 23.652, 95% CI = 2.270 ~ 246.487, *P* = 0.008) and CR (β = 31.676, se = 13.642, *P* = 0.020), minerals and other dietary supplements: calcium (OR = 17.540,95% CI = 12.255 ~ 25.103, *P* = 2.83×10^-55^), bread intake (OR = 1.719, 95% CI = 1.085 ~ 2.724, *P* = 0.021) and milk type: never/rarely drink milk (OR = 218.448, 95% CI = 6.249 ~ 7636.549, *P* = 0.003) would increase the risk of BCR. See Table S19 for details. In addition, using IVs of dietary habits, we estimated the causal effect of exposure on potential mediators. In this section, we identified 38 causal relationships for 14 dietary habits and 9 bacteria (Table S20).

**1.3 References**

Baxter, N.T., Koumpouras, C.C., *et al.* (2016) ‘DNA from fecal immunochemical test can replace stool for detection of colonic lesions using a microbiota-based model’, *Microbiome*, 4(1), p. 59. Available at: https://doi.org/10.1186/s40168-016-0205-y.

Baxter, N.T., Ruffin, M.T., *et al.* (2016) ‘Microbiota-based model improves the sensitivity of fecal immunochemical test for detecting colonic lesions’, *Genome Medicine*, 8(1), p. 37. Available at: https://doi.org/10.1186/s13073-016-0290-3.

Bolyen, E. *et al.* (2019) ‘Reproducible, interactive, scalable and extensible microbiome data science using QIIME 2’, *Nature Biotechnology*, 37(8), pp. 852–857. Available at: https://doi.org/10.1038/s41587-019-0209-9.

Dadkhah, E. *et al.* (2019) ‘Gut microbiome identifies risk for colorectal polyps’, *BMJ open gastroenterology*, 6(1), p. e000297. Available at: https://doi.org/10.1136/bmjgast-2019-000297.

Hannigan, G.D. *et al.* (2018) ‘Diagnostic potential and interactive dynamics of the colorectal cancer virome’, *mBio*, 9(6), pp. e02248-18. Available at: https://doi.org/10.1128/mBio.02248-18.

Sze, M.A. *et al.* (2017) ‘Normalization of the microbiota in patients after treatment for colonic lesions’, *Microbiome*, 5(1), p. 150. Available at: https://doi.org/10.1186/s40168-017-0366-3.

Xiao, L., Zhang, F. and Zhao, F. (2022) ‘Large-scale microbiome data integration enables robust biomarker identification’, *Nature Computational Science*, 2(5), pp. 307–316. Available at: https://doi.org/10.1038/s43588-022-00247-8.

Zeller, G. *et al.* (2014) ‘Potential of fecal microbiota for early-stage detection of colorectal cancer’, *Molecular Systems Biology*, 10(11), p. 766. Available at: https://doi.org/10.15252/msb.20145645.

Zuber, V. *et al.* (2020) ‘Selecting likely causal risk factors from high-throughput experiments using multivariable Mendelian randomization’, *Nature Communications*, 11, p. 29. Available at: https://doi.org/10.1038/s41467-019-13870-3.

# Supplementary Figures and Tables

## Supplementary Figures


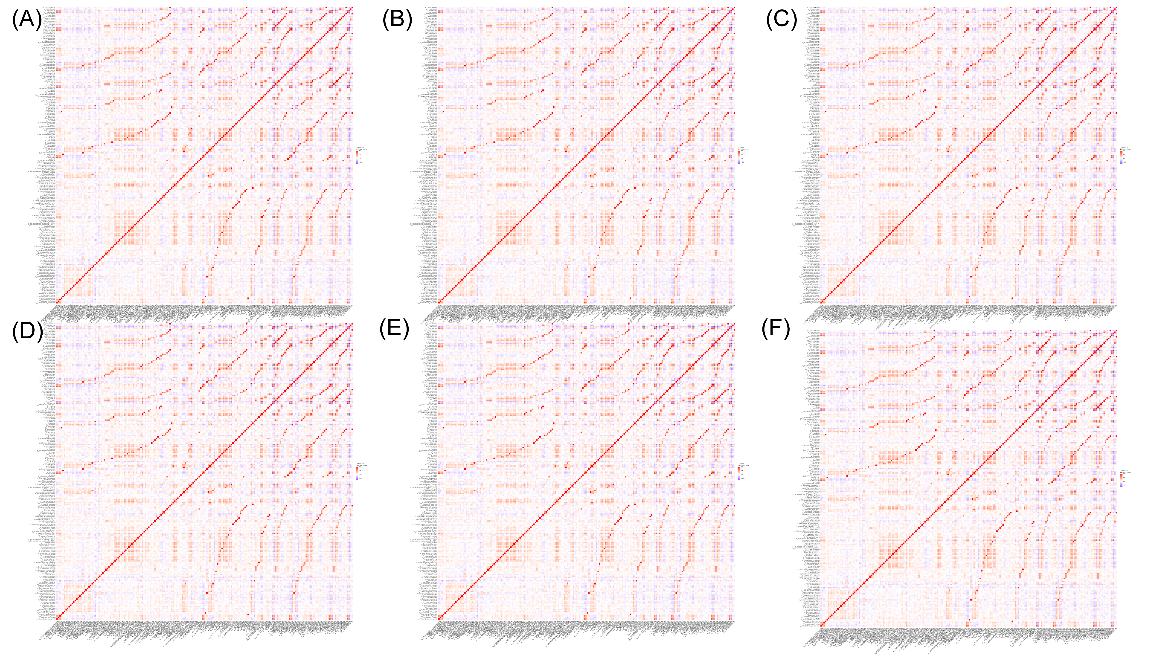


**Figure S1.** Genetic correlations between gut bacteria. (A) Genetic correlations betw een 194 gut bacterial measurements based on CRC n = 432 genetic variants used as instrumental variables; (B) Genetic correlation between 194 gut bacterial measurements based on CC n = 432 genetic variants used as instrumental variables; (C) Genetic correlation between 194 gut bacterial measurements based on CR n = 432 genetic variants used as instrumental variables; (D) Genetic correlation between 194 gut bacterial measurements based on BCR n = 432 genetic variants used as instrumental variables; (E) Genetic correlation between 194 gut bacterial measurements based on BC n = 432 genetic variants used as instrumental variables; (F) Genetic correlation between 194 gut bacterial measurements based on BR n = 432 genetic variants used as instrumental variables

**
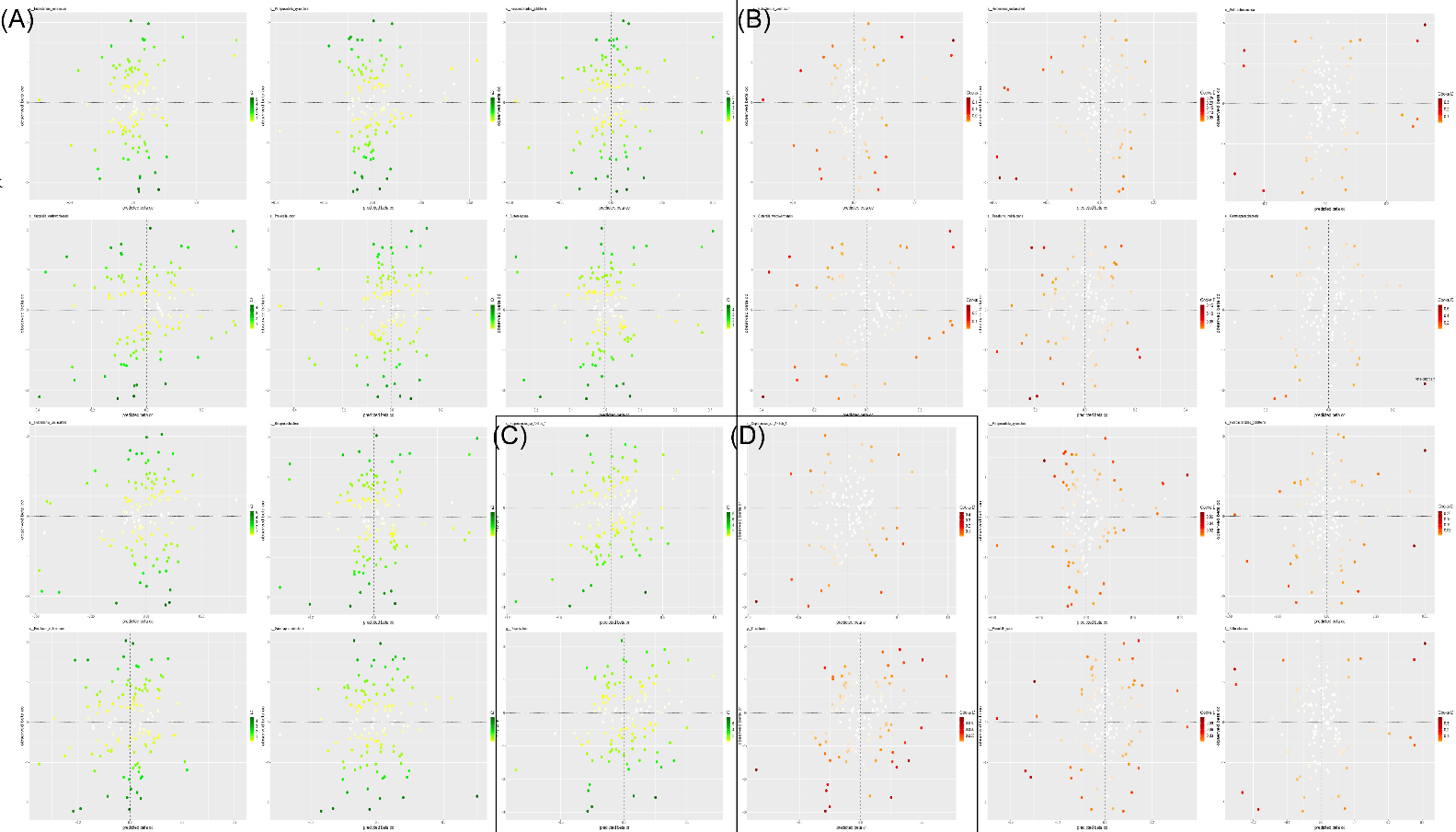
**

**Figure S2.** Diagnostic plot of outliers and influential genetic variants in the underlying biomarker rankmethod Based. on the model, the predicted association with the outcome (x-axis) and the association with the outcome showing all genetic variants observed (y-axis) are plotted. This is the highest-ranked model when it comes to maintaining outlier and influential genetic variants in the analysis. The color code shows that the (A)(CC), (C)(CR) q statistic is an outlier, and (B)(CC), (D)(CR) Cook distance is the influence point. Any genetic variation with a q value greater than 10 or a cook distance greater than the median of the associated F distribution is marked by a marker.

**
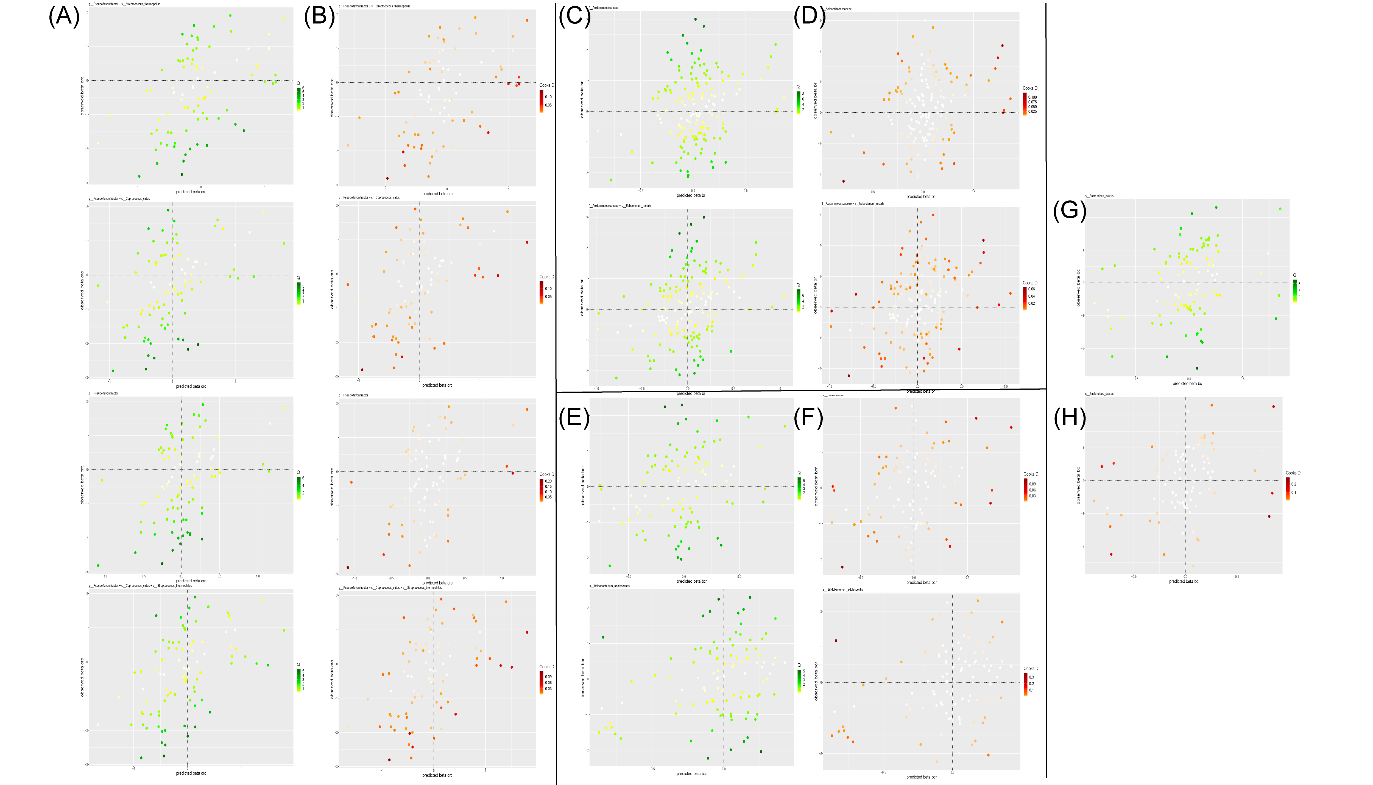
**

**Figure S3.** Diagnostic plot of outliers and influential genetic variants in the underlying biomarker ranking method. Based on the model, the predicted association with the outcome (x-axis) and the observed association with the outcome showing all genetic variants (y-axis) are plotted. This is the highest-ranked model when it comes to maintaining outlier and influential genetic variants in the analysis. The color codes show that the (A)(CRC), (C)(BR), (E)(CR), (G)(BC)q statistic are outliers, and (B)(CRC), (D)(BR), (F)(CR), (H)(BC) Cook distance is the impact point. Any genetic variation with a q value greater than 10 or a cook distance greater than the median of the associated F distribution is marked by a marker.

**
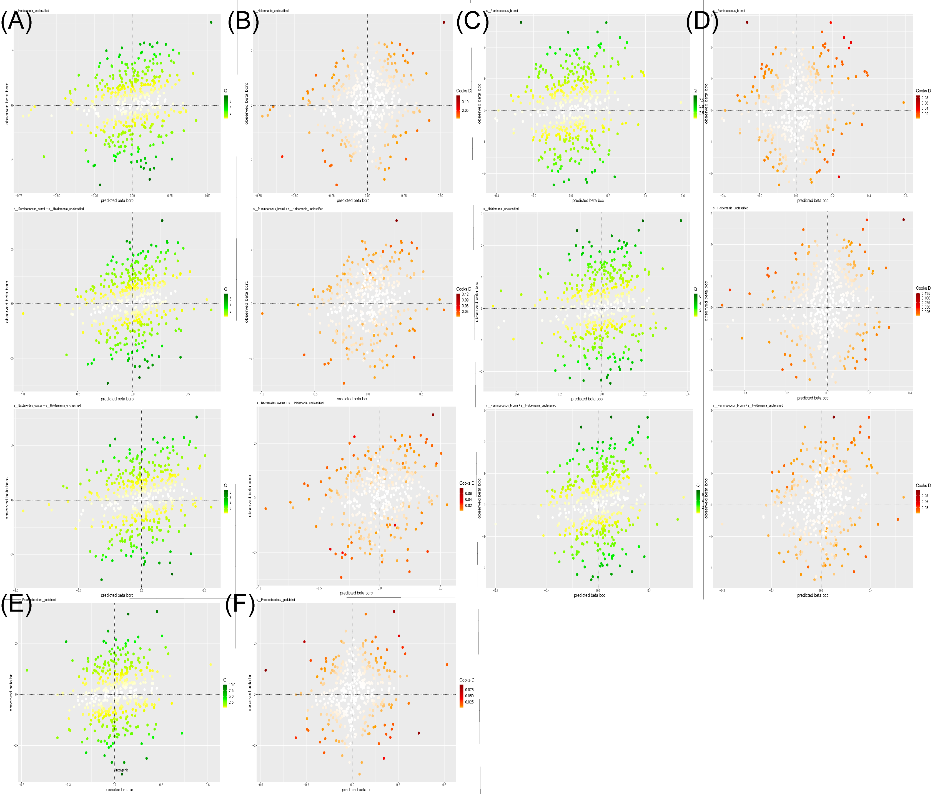
**

**Figure S4.** Diagnostic plot of outliers and influential genetic variants in the microbial risk factor determination method. Based on the model, the predicted association with the outcome (x-axis) and the association with the outcome showing all genetic variants observed (y-axis) are plotted. This is the highest-ranked model when it comes to maintaining outlier and influential genetic variants in the analysis. The color codes show that the (A)(BCR), (C)(BC), (E)(BR)q statistic are outliers, and (B)(BCR), (D)(BC), (F)(BR) Cook distance is the influence point. Any genetic variation with a q value greater than 10 or a cook distance greater than the median of the associated F distribution is marked by a marker.


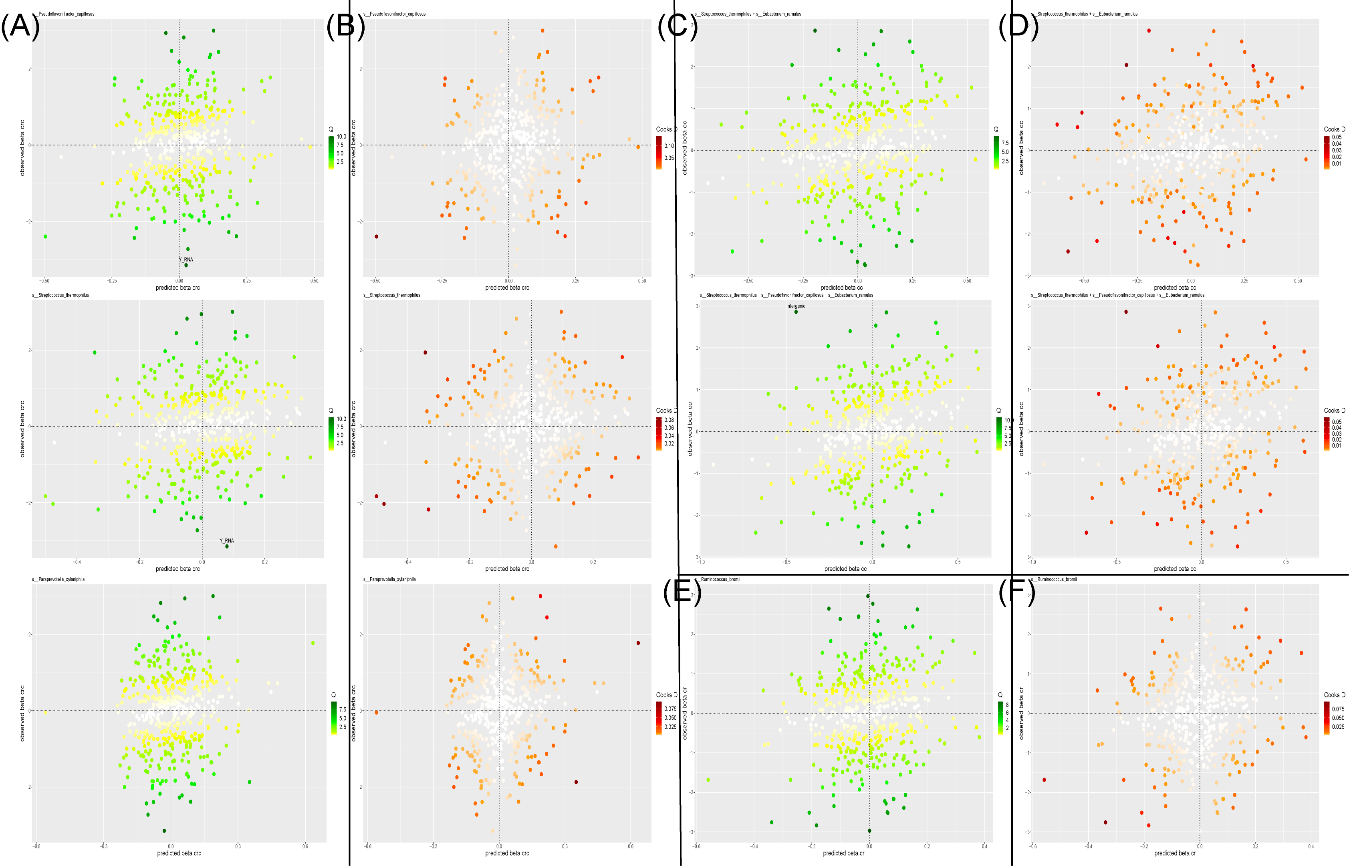


**Figure S5.** Diagnostic plot of outliers and influential genetic variants in the underlying biomarker sequencing method. redictive association with outcome (x-axis) based on the model and association with outcome showing observed for all genetic variants (y-axis). This is the highest-ranked model when it comes to maintaining outlier and influential genetic variants in the analysis. The color code shows that the (A)(CRC), (C)(CC), (E)(CR)q statistic is an outlier, and (B)(CRC), (D)(CC), (F)(CR) Cook distance is the influence point. Any genetic variation with a q value greater than 10 or a cook distance greater than the median of the associated F distribution is marked by a marker.


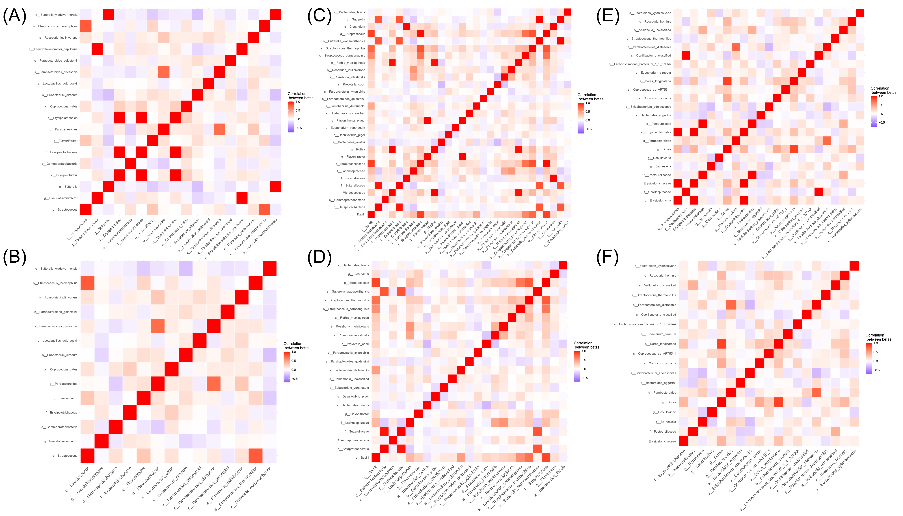


**Figure S6.** Genetic correlation heat map between malignancy-associated gut bacteria in potential biomarker sequencing methods. (A) Genetic correlation between 18 measures of gut bacteria associated with CRC based on n = 87 genetic variants used as instrumental variables; (B) Genetic correlation between 14 measures of gut bacteria associated with CRC based on n = 87 genetic variants used as instrumental variables (after trimming microorganisms with genetic correlation greater than 0.985) ;(C) genetic correlation between 29 measures of gut bacteria associated with CC based on n = 135 genetic variants used as instrumental variables; (D) Genetic correlation between 23 measures of gut bacteria associated with CC based on n = 123 genetic variants used as instrumental variables (after trimming microorganisms with genetic correlation greater than 0.985);(E) genetic correlation between 23 measures of gut bacteria associated with CR based on n = 125 genetic variants used as instrumental variables; (F) Genetic correlation between 19 measures of gut bacteria associated with CR based on n = 125 genetic variants used as instrumental variables (after pruning microorganisms with genetic correlation greater than 0.985)


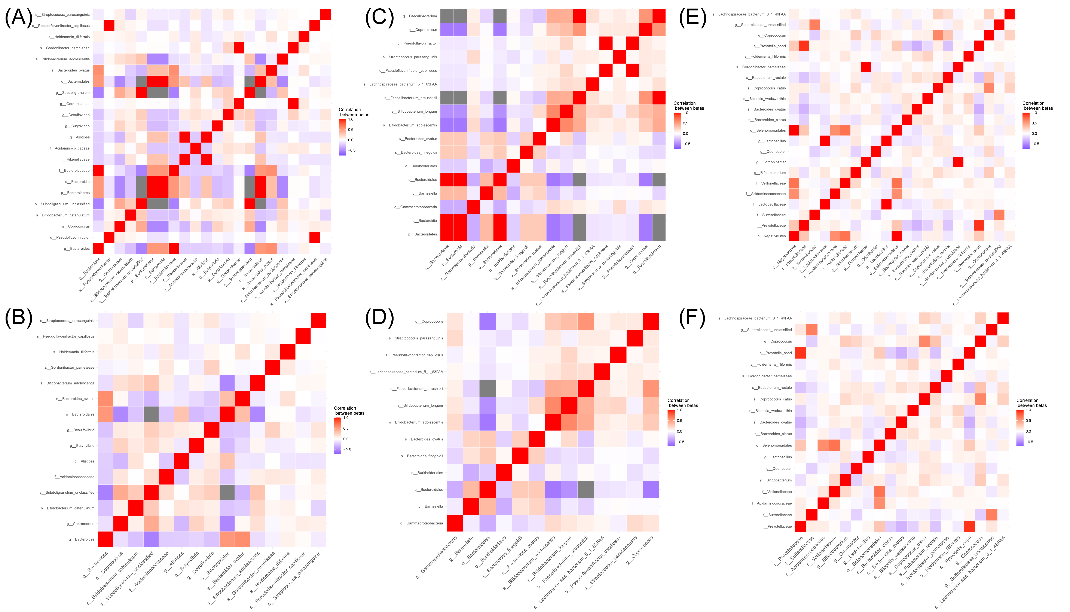


**Figure S7.** Genetic correlation heat map between benign tumor-associated gut bacteria in potential biomarker sequencing methods. (A) Genetic correlation between 22 BCR-related measures based on n = 103 genetic variants used as instrumental variables; (B) Genetic correlation between 15 BCR-associated measures based on n = 102 genetic variants used as instrumental variables (after trimming microorganisms with genetic correlations greater than 0.985) ;(C) genetic correlations between 17 BC-associated gut bacterial measurements based on n = 101 genetic variants used as instrumental variables; (D) Genetic correlation between 13 BC-associated gut bacterial measurements based on n = 100 genetic variants used as instrumental variables (after trimming microorganisms with genetic correlation greater than 0.985) ;(E) genetic correlation between 22 gut bacterial measurements associated with BR based on n = 158 genetic variants used as instrumental variables; (F) Genetic correlation between 19 measures of gut bacteria associated with BR based on n = 157 genetic variants used as instrumental variables (after trimming microorganisms with genetic correlation greater than 0.985)

## Supplementary Tables

**Table S1. Cohort data details for analysis.**

| **Cohort** | **Bioproject** | **Sample size** | | | | **Population** |
| --- | --- | --- | --- | --- | --- | --- |
|  |  | **Healthy** | **Adenoma** | **Cancer** | **Total samplesize** |  |
| CRC1 | PRJNA389927 | 30 | 30 | 30 | 90 | European |
| CRC2 | PRJEB6070 | 50 | 38 | 41 | 129 | European |
| CRC3 | PRJNA362366 | 0 | 41 | 26 | 67 | European |
| CRC4 | PRJNA290926 | 172 | 198 | 120 | 490 | European |
| CRC5 | PRJNA534511 | 76 | 107 | 0 | 183 | European |
| CRC6 | PRJNA318004 | 141 | 162 | 101 | 404 | European |
| Total samplesize |  | 469 | 576 | 318 | 1363 |  |

*Note:* CRC: colorectal cancer.

**Table S2. Prior probability threshold for *Q*-statistics and *Cd* identification of anomalies and strong influence points in sensitivity analysis.**

| **Disease** | **CRC** | **CC** | **CR** | **BCR** | **BC** | **BR** |
| --- | --- | --- | --- | --- | --- | --- |
| **Prior probability threshold** | 0.01 | 0.005 | 0.005 | 0.02 | 0.01 | 0.01 |

*Note:* CRC: colorectal cancer.

**Table S3. Instrumental variables for gut microbial traits.**

| **Exposure** | **SNP** |
| --- | --- |
| c__Actinobacteria | rs182549, rs74452406, rs795981, rs62347983, rs318801, rs35344390, rs2197344, rs61939486, rs926010, rs12955365, rs6044857, |
| c__Bacteroidia | rs2807619, rs10158939, rs1023692, rs9843097, rs2603095, rs73098732, rs13283363, rs74949229, rs10836532, rs28538857, rs11657406, rs783253, rs2698177, |
| c__Bacilli | rs12064418, rs75005855, rs11694441, rs76752365, rs11744900, rs1206113, rs74520302, rs10761245, rs1249137, rs77655905, rs4903903, rs1400577, rs10404584, rs3892326, |
| c__Clostridia | rs1023692, rs7794348, rs674292, rs12675379, rs74949229, rs34566098, rs10766557, |
| c__Erysipelotrichia | rs78685125, rs4975752, rs733330, rs75298326, rs11652559, rs62131327, rs73037929, rs73114746, |
| c__Negativicutes | rs12472147, rs176564, rs10736970, rs78791321, rs77764206, rs2282037, rs12979658, |
| c__Betaproteobacteria | rs12734494, rs9428844, rs13071362, rs62351959, rs12646537, rs59033852, rs11626416, rs78026425, rs9673456, rs79598028, rs74034215, rs2834333, rs713716, |
| c__Deltaproteobacteria | rs2990682, rs73966738, rs10087056, rs11219805, rs237461, |
| c__Gammaproteobacteria | rs12726307, rs292226, rs80113686, rs117076107, |
| c__Verrucomicrobiae | rs12710731, rs4670221, rs6823767, rs68058313, rs1298928, rs9368149, rs36119306, rs7912369, rs4075877, rs166570, rs55926131, rs35895642, rs1532932, rs79041579, rs2523176, |
| g__Gordonibacter | rs12637754, rs2966752, rs62481637, rs7553861, |
| g__Butyrivibrio | rs111544141, rs13055049, rs1389373, rs1652670, rs17692191, rs17709673, rs3807927, rs4904659, rs4974008, rs9459627, |
| g__Lactobacillus | rs112241999, rs1149732, rs12204031, rs17457331, rs2332915, rs2598336, rs278502, rs3135176, rs34185363, rs6684536, rs76832256, rs9559303, |
| g__Rothia | rs10513070, rs17049177, rs616801, rs709159, rs769155, rs80010435, rs888195, rs9268614, |
| g__Phascolarctobacterium | rs116643293, rs12592302, rs1355585, rs173961, rs2395296, rs3798491, rs67541505, rs74338790, rs79825773, rs9389726, |
| g__Haemophilus | rs56162052, rs7132825, rs76236729, rs7651754, rs983309, |
| g__Veillonella | rs12978412, rs2535959, rs3000817, rs4462945, rs7159466, rs72743290, rs9847942, |
| g__Flavonifractor | rs13061928, rs1872436, rs5768347, rs77544959, rs9848037, |
| g__Oxalobacter | rs10865362, rs11068499, rs12725104, rs17562516, rs1861231, rs28740947, rs35921003, rs3745098, rs4889373, rs73918563, |
| g__Desulfovibrio | rs12466754, rs2477580, |
| g__Burkholderiales_noname | rs10507465, rs10895784, rs11144350, rs12941005, rs13257050, rs1533824, rs16923154, rs1792325, rs1860691, rs4717625, rs6664382, rs73653217, rs9872584, |
| g__Dialister | rs2499076, rs2967172, rs35335925, rs4832418, rs73519430, rs7761067, |
| g__Paraprevotella | rs10760885, rs115309509, rs12895134, rs246783, rs2882286, rs4767918, rs61407789, rs6465823, rs704272, rs72810711, rs891501, |
| g__Akkermansia | rs12710731, rs1298928, rs1532932, rs166570, rs2523176, rs35895642, rs36119306, rs4075877, rs4670221, rs55926131, rs68058313, rs6823767, rs79041579, rs7912369, rs9368149, |
| g__Blautia | rs2120865, rs28762974, rs4445887, rs62475831, rs62551178, rs6783841, rs6809294, rs6990479, rs74083978, rs7591885, rs77623116, rs9459138, |
| g__Ruminococcus | rs10924209, rs13420244, rs3013111, rs6039702, rs62048493, rs6426007, rs6750178, rs74683729, rs76382119, |
| g__Odoribacter | rs1035637, rs17507951, rs2605503, rs28376149, rs3103001, rs58045871, rs6461301, rs71492034, rs7298214, rs935417, |
| g__Barnesiella | rs10026787, rs1078988, rs115004061, rs118183316, rs11851037, rs34437635, rs62135856, rs67396441, rs6920861, rs700801, rs74448867, rs78335793, rs8029854, rs9884588, |
| g__Bilophila | rs12913320, rs13009600, rs1780628, rs2579005, rs2990682, rs6958516, rs77848425, |
| g__Dorea | rs10927176, rs12541342, rs404634, rs56398621, rs74448283, rs7479452, rs9560166, |
| g__Bacteroidales_noname | rs12449080, rs12602625, rs2008778, rs2465095, rs2700958, rs3106091, rs35445518, rs4294998, rs55712042, |
| g__Collinsella | rs10070311, rs11782083, rs4075691, rs4933278, rs550057, rs6099022, rs7147611, rs7831606, |
| g__Bifidobacterium | rs10455532, rs10873640, rs11732081, rs12657170, rs182549, rs4472164, rs4859454, rs61939486, rs72993945, rs74452406, |
| g__Parabacteroides | rs10854303, rs11083824, rs113638576, rs11603389, rs16848174, rs17639446, rs17706792, rs315588, rs541877, rs56238206, rs6910508, rs9409512, rs9663125, |
| g__Lachnospiraceae_noname | rs11208867, rs1814009, rs2119879, rs2341789, rs61571947, rs6575490, rs77194050, |
| g__Eubacterium | rs17218256, rs1942648, rs2252668, rs3898456, rs4113953, rs6021384, rs9600403, |
| g__Roseburia | rs11748902, rs11789010, rs1258842, rs12653554, rs13019272, rs2210174, rs57070158, rs61217796, rs66973779, rs72963797, rs73106151, rs7404803, rs7819696, rs7985034, |
| g__Oscillibacter | rs10769221, rs11087270, rs12446334, rs13119423, rs34865636, rs55682375, rs56001939, rs6821986, rs7255582, |
| g__Alistipes | rs11211406, rs2465506, rs4949447, rs6131687, rs61881300, rs73344869, rs74459573, rs77318370, rs9511112, rs952822, |
| g__Subdoligranulum | rs10836532, rs11578868, rs11726764, rs123043, rs12622085, rs1850270, rs2105601, rs225280, rs2317916, rs4721723, rs4816989, rs6830068, rs7000832, rs7104786, rs73044678, |
| g__Escherichia | rs112572980, rs115534005, rs11676886, rs11731238, rs1246347, rs13138313, rs17058001, rs1983438, rs2362966, rs2791437, rs62242204, |
| g__Adlercreutzia | rs7532302, rs148883344, rs72682613, rs9347737, rs62529678, rs609909, rs73546364, |
| g__Eggerthella | rs141306664, rs231723, rs62358729, rs35501864, rs111842857, rs77214081, |
| g__Bacteroides | rs2774282, rs742682, rs28414316, rs62365577, rs73046099, rs56178001, rs80306758, rs4278144, rs680138, rs2571, rs505128, rs71446723, rs61510729, rs9564193, rs13045121, |
| g__Coprobacter | rs75238635, rs112428203, rs2697923, rs1896610, rs79594952, rs12232484, rs12463384, |
| g__Prevotella | rs6745806, rs79216963, rs4503137, rs139901450, rs73654027, rs9517773, rs2330438, rs6005414, |
| g__Streptococcus | rs9828991, rs9398452, rs528874, rs2882716, rs73447940, rs41294029, rs56025506, rs1901115, rs34829339, rs3892326, |
| g__Clostridium | rs1572504, rs28577018, rs11969660, rs10106275, rs55834012, rs111604729, rs12913822, rs12932210, rs7214893, |
| g__Pseudoflavonifractor | rs6778836, rs9290954, rs75202637, rs1182140, rs7790846, rs10236678, rs4424244, rs7990301, rs11074766, |
| g__Coprococcus | rs17639938, rs10460443, rs6772892, rs4861985, rs386190, rs10234541, rs12577679, rs1844921, rs7964677, rs17276797, rs117817822, |
| g__Anaerotruncus | rs61824334, rs6440148, rs1044858, rs4747349, rs62075885, rs6020721, |
| g__Faecalibacterium | rs840787, rs61798216, rs12197028, rs74949229, rs12415307, rs2107539, rs3785822, rs36720, |
| g__Ruminococcaceae_noname | rs114446285, rs12621499, rs570295, rs3916427, rs9414689, rs9508731, rs11626630, rs4416113, rs4805798, rs4239490, |
| g__Erysipelotrichaceae_noname | rs10915295, rs71510947, rs7671513, rs2926850, rs13238712, rs6557756, rs112684584, rs9316496, rs61956724, rs444953, rs4338887, rs139790375, |
| g__Holdemania | rs10027188, rs140472622, |
| g__Parasutterella | rs10177566, rs34613239, rs9847008, rs11715674, rs10036326, rs10981640, rs11595913, rs11067048, rs56085972, rs17472393, rs62003625, rs6026349, |
| g__Sutterella | rs61790480, rs6478652, rs1421280, rs7179502, |
| g__Sutterellaceae_unclassified | rs116693483, rs56246718, rs62501151, rs1685109, rs10898147, rs1985586, |
| s__Bacteroides_nordii | rs8098967, |
| s__Coprococcus_sp_ART55_1 | rs12329315, rs148177109, rs35529952, rs3801563, |
| s__Lachnospiraceae_bacterium_5_1_63FAA | rs10785016, rs12047309, rs147524453, rs17661696, rs3935551, |
| s__Gordonibacter_pamelaeae | rs12637754, rs2966752, rs62481637, rs7553861, |
| s__Lactobacillus_delbrueckii | rs11113894, rs1118894, rs11734017, rs12488234, rs1458987, rs2332915, rs278731, rs28635178, rs299199, rs35870858, rs4333664, rs4433502, rs4450413, rs60200511, rs6517636, rs735027, |
| s__Lachnospiraceae_bacterium_1_1_57FAA | rs74322752, rs76321722, |
| s__Bacteroides_salyersiae | rs117662076, rs12534702, rs1265300, rs12896430, rs16845299, rs17111728, rs3776996, rs412657, rs76194327, rs780878, rs9960494, |
| s__Butyrivibrio_crossotus | rs111544141, rs114804117, rs13055049, rs1389373, rs17692191, rs17709673, rs3807927, rs3935, rs4900028, rs56347647, rs9459627, |
| s__Parabacteroides_johnsonii | rs114845775, rs11724182, rs11806181, rs12101679, rs13265628, rs17693136, rs568692, rs598632, rs62163269, |
| s__Ruminococcaceae_bacterium_D16 | rs114446285, rs11626630, rs12621499, rs3916427, rs4239490, rs4416113, rs4805013, rs570295, rs9414689, rs9508731, |
| s__Streptococcus_parasanguinis | rs117329402, rs1893286, rs6481791, |
| s__Parabacteroides_goldsteinii | rs12617695, rs12879040, rs1357368, rs2898012, rs3973922, rs8006060, rs988862, |
| s__Bacteroides_intestinalis | rs1042840, rs4793180, rs4814260, rs72656234, |
| s__Streptococcus_salivarius | rs1810052, |
| s__Bacteroides_clarus | rs1157623, rs11676681, rs1470205, rs1667938, rs2989472, rs450741, rs4867972, rs61944136, rs814760, |
| s__Bacteroides_finegoldii | rs10215754, rs10744662, rs10970830, rs111426764, rs116556966, rs11773071, rs12889912, rs1430034, rs2393782, rs2717057, rs4809943, rs496238, rs58346872, rs58668331, rs62210593, rs66535021, rs73188646, rs74904967, |
| s__Eggerthella_unclassified | rs1245457, rs141306664, rs35315167, |
| s__Rothia_mucilaginosa | rs1576176, rs17049177, rs616801, rs888195, |
| s__Lachnospiraceae_bacterium_8_1_57FAA | rs11127034, rs11223731, rs11581135, rs12986007, rs1346173, rs140741191, rs16899159, rs28521978, rs3750889, rs4559153, rs4970837, rs61854800, rs61895387, rs752704, rs76588436, rs78404253, rs8089654, |
| s__Bifidobacterium_bifidum | rs10506461, rs11630244, rs12512757, rs17453521, rs4861647, rs56249713, rs57711736, rs59340188, rs62555927, rs722497, rs7334864, rs76631862, rs8176645, rs849116, rs9525230, rs9848175, |
| s__Streptococcus_thermophilus | rs13086575, rs6900225, rs74654613, rs7524171, |
| s__Eubacterium_biforme | rs11044047, rs1978600, rs2293361, rs56702013, rs720784, rs72891473, rs79529135, rs9972236, |
| s__Phascolarctobacterium_succinatutens | rs116643293, rs12592302, rs1355585, rs173961, rs2395296, rs3798491, rs67541505, rs74338790, rs79825773, rs9389726, |
| s__Holdemania_filiformis | rs113638576, rs72661091, rs77053869, |
| s__Haemophilus_parainfluenzae | rs28693594, rs56162052, rs7132825, rs76236729, rs7651754, rs781762, |
| s__Holdemania_unclassified | rs10123164, rs10247831, rs117158611, rs28368536, rs310007, rs4868427, rs4922067, rs67887362, rs7169918, rs746810, rs78014650, |
| s__Veillonella_unclassified | rs11102545, rs12978412, rs3000812, rs325705, rs62111248, rs6548355, rs6601127, rs7274039, rs72743290, rs8017450, rs9797071, |
| s__Clostridium_leptum | rs11858399, rs17398712, rs67194754, rs7073292, rs72662817, rs79201749, rs9356977, |
| s__Paraprevotella_xylaniphila | rs113640907, rs2005815, rs311865, rs3919827, rs4709313, rs595638, rs6052686, rs61801633, rs62240505, rs6970331, rs9314321, |
| s__Flavonifractor_plautii | rs13061928, rs1872436, rs5768347, rs77544959, rs9513795, rs9848037, |
| s__Eubacterium_ventriosum | rs1011345, rs10502943, rs12124525, rs3815679, rs73040331, rs79951774, |
| s__Desulfovibrio_piger | rs10030640, rs196229, rs4854535, rs61818112, rs75364852, rs77902968, |
| s__Bacteroides_eggerthii | rs114970711, rs13312424, rs1861386, rs2487307, rs286623, rs4767925, rs7791761, rs9536634, |
| s__Ruminococcus_callidus | rs12098699, rs1321631, rs35200886, rs4268390, rs79207650, |
| s__Oxalobacter_formigenes | rs10865362, rs11068499, rs12725104, rs17562516, rs1861231, rs35921003, rs3745098, rs4889373, rs73918563, |
| s__Roseburia_unclassified | rs13127387, rs17665612, rs2121787, rs2568643, rs4883997, rs57978465, rs62006238, rs7217715, rs7289868, rs76776955, rs7720468, rs8013597, rs856250, rs894795, |
| s__Alistipes_sp_AP11 | rs11664566, rs12022867, rs13333427, rs1553042, rs2293915, rs28450200, rs34886974, rs55790429, rs71385354, rs73147950, rs9958338, |
| s__Dialister_invisus | rs2499076, rs2967172, rs4832418, rs512089, rs55976608, rs72748807, |
| s__Bacteroides_fragilis | rs10137996, rs11770593, rs12565327, rs1285852, rs12900979, rs3797248, rs3885195, rs4430574, rs4772337, rs4902984, rs55835510, rs6458025, rs6909908, rs76722389, rs77062729, rs912, rs9818008, |
| s__Burkholderiales_bacterium_1_1_47 | rs10507465, rs10895784, rs11144350, rs12941005, rs13257050, rs1533824, rs16923154, rs1792325, rs1860691, rs4717625, rs6664382, rs73653217, rs9872584, |
| s__Bacteroides_massiliensis | rs112691179, rs12621563, rs17807243, rs4916966, rs4937922, rs61944855, rs6532564, |
| s__Pseudoflavonifractor_capillosus | rs10236678, rs11074766, rs1182140, rs4352232, rs4424244, rs6761789, rs6778836, rs74412941, rs75202637, rs7790846, rs7990301, |
| s__Adlercreutzia_equolifaciens | rs148883344, rs609909, rs62529678, rs72682613, rs73546364, rs7532302, rs9347737, |
| s__Bacteroides_cellulosilyticus | rs10450862, rs11096868, rs116972192, rs12976637, rs2026546, rs2411041, rs2838820, rs35946101, rs4857402, rs6049103, rs62338073, rs7030947, rs7099427, |
| s__Eubacterium_ramulus | rs10848471, rs10961097, rs113157937, rs12474753, rs12935244, rs2368038, rs6590368, rs6731499, rs75578397, rs9804152, |
| s__Eubacterium_hallii | rs10014686, rs10848037, rs1330325, rs17568569, rs17749540, rs1952183, rs2871118, rs2901343, rs4692552, rs62468608, rs6487088, rs73443336, |
| s__Parabacteroides_unclassified | rs10281779, rs10911039, rs12697058, rs12809872, rs1580572, rs2449506, rs303729, rs4943000, |
| s__Paraprevotella_unclassified | rs10760885, rs11938983, rs12895134, rs1969724, rs246783, rs4767918, rs6465823, rs7015972, rs72810711, rs891501, |
| s__Escherichia_coli | rs10024750, rs10752292, rs11149171, rs12623651, rs17729286, rs2600191, rs62578888, rs6857075, rs731362, rs75297146, rs78009268, rs9466145, |
| s__Parasutterella_excrementihominis | rs10036326, rs10177566, rs10981640, rs11067048, rs11595913, rs11715674, rs34613239, rs56085972, rs6026349, rs62003625, rs7320949, rs9847008, |
| s__Dorea_formicigenerans | rs11631507, rs73146738, |
| s__Ruminococcus_torques | rs10832975, rs11161562, rs13109150, rs17696544, rs2229594, rs4457100, rs61213519, rs775133, |
| s__Coprococcus_catus | rs10107806, rs341677, rs34169081, rs3770472, rs417265, |
| s__Ruminococcus_bromii | rs10864073, rs12041621, rs12131104, rs1884673, rs34762184, rs4884323, rs9865704, |
| s__Roseburia_intestinalis | rs12592099, rs2087796, rs4834490, rs6871001, rs7112918, rs72697383, rs76557325, rs778342, rs8006559, |
| s__Alistipes_indistinctus | rs10966460, rs11957434, rs12332306, rs1362650, rs1443711, rs2055593, rs2789623, rs4897508, rs62390586, rs62493320, rs73102812, rs8015391, |
| s__Sutterella_wadsworthensis | rs1421280, rs61790480, rs6478652, rs7179502, |
| s__Bilophila_wadsworthia | rs10276776, rs11768363, rs12724599, rs1688043, rs34196897, rs35334318, rs6852392, |
| s__Bacteroides_caccae | rs11654291, rs12367159, rs12739496, rs144905245, rs149486873, rs2204037, rs2457684, rs34612513, rs868452, |
| s__Eubacterium_siraeum | rs11002091, rs117632297, rs13040204, rs1358421, rs2660277, rs2717881, rs28706788, rs2906787, rs35239958, rs544964, rs66806908, rs74413687, rs7665837, rs8050653, rs9805811, |
| s__Ruminococcus_obeum | rs1005544, rs11072031, rs12581302, rs140443231, rs17773250, rs28802725, rs36122201, rs4445887, rs4761660, rs6570332, rs708686, rs73098536, rs7793841, |
| s__Dorea_longicatena | rs11028061, rs11759411, rs12499537, rs13106159, rs17475926, rs2935621, rs6729788, rs6957565, rs8032562, rs9292972, |
| s__Prevotella_copri | rs12657476, rs12870360, rs2330438, rs2421570, rs4503137, rs55749361, rs6005414, rs6745806, rs7088001, rs72799593, rs73654027, rs79216963, rs7930251, rs9517773, |
| s__Parabacteroides_merdae | rs10733815, rs12116657, rs148330790, rs1508792, rs2005874, rs4237122, rs66722558, rs702101, rs7631540, |
| s__Akkermansia_muciniphila | rs12710731, rs1298928, rs1532932, rs166570, rs2523176, rs35895642, rs36119306, rs4075877, rs4670221, rs55926131, rs68058313, rs79041579, rs7912369, rs9368149, |
| s__Parabacteroides_distasonis | rs3735683, rs3814989, rs4618483, rs60248492, rs62227335, rs6603134, rs6688656, rs6970565, rs7853135, |
| s__Eubacterium_eligens | rs10927575, rs11678512, rs11933020, rs13060820, rs2184120, rs2426807, rs396720, rs4268153, rs4775557, rs56105508, rs6922056, rs77362317, rs79969063, |
| s__Bifidobacterium_adolescentis | rs10063054, rs11986537, rs144770262, rs4110696, rs4256405, rs4351047, rs4646215, rs4850073, rs6455422, rs6754311, rs74394156, rs8045380, |
| s__Alistipes_senegalensis | rs13381162, rs13393033, rs3786306, rs4935713, rs56168514, rs592284, rs7155195, rs72643019, rs73083250, rs7944200, rs852540, rs9573198, |
| s__Roseburia_inulinivorans | rs12418335, rs2273934, rs7630416, rs77776469, |
| s__Bifidobacterium_longum | rs10033190, rs10843776, rs11721919, rs11786426, rs13264085, rs13391731, rs1446585, rs6546996, rs6780177, rs73412051, rs77019567, rs7761703, |
| s__Bilophila_unclassified | rs11067415, rs11260998, rs12913320, rs13009600, rs2579005, rs2990682, rs6127771, rs6958516, rs765025, rs77848425, |
| s__Bacteroides_ovatus | rs1021440, rs112057053, rs11873510, rs190949005, rs2642158, rs303925, rs4867356, rs59809293, rs67659622, rs72677788, rs73240572, rs75642318, rs7866097, rs9304475, rs9684138, |
| s__Bacteroides_dorei | rs10119693, rs11037453, rs11708946, rs227442, rs2291958, rs2721884, rs387171, rs62523331, rs6585011, rs72819063, rs795988, rs7998315, rs838134, rs9313811, rs9518837, |
| s__Collinsella_aerofaciens | rs11736801, rs17553786, rs386274, rs550057, rs61877169, rs61940669, rs7147611, rs72774015, rs72845940, rs7831606, |
| s__Bacteroidales_bacterium_ph8 | rs12449080, rs12602625, rs2008778, rs203770, rs2465095, rs2700958, rs35445518, rs55712042, |
| s__Alistipes_shahii | rs11161058, rs12134195, rs17070727, rs1728059, rs34355813, rs34990336, rs4693197, rs6508629, rs7043141, rs79555856, rs9886784, |
| s__Alistipes_onderdonkii | rs11041738, rs11104946, rs117024060, rs488197, rs7660775, rs7804962, rs8011841, |
| s__Bacteroides_vulgatus | rs11646266, rs13193662, rs142222485, rs1498839, rs1514626, rs56282904, rs6044532, rs6732796, |
| s__Eubacterium_rectale | rs12807499, rs1452438, rs1867204, rs1890115, rs1939678, rs2449527, rs36042240, rs4333970, rs6021385, rs6695790, rs72645666, |
| s__Oscillibacter_unclassified | rs10769221, rs11087270, rs12446334, rs13119423, rs34865636, rs55682375, rs56001939, rs7255582, |
| s__Faecalibacterium_prausnitzii | rs10113323, rs12197028, rs12415307, rs2107539, rs36720, rs3785822, rs61798216, rs74949229, rs840787, |
| s__Odoribacter_splanchnicus | rs11111, rs117633633, rs13096936, rs152172, rs2383400, rs2470427, rs495710, rs5754282, rs6583351, rs7011780, rs7019674, rs73619447, rs74682567, rs76880769, |
| s__Coprobacter_fastidiosus | rs112428203, rs12232484, rs12463384, rs1896610, rs2697923, rs75238635, rs79594952, |
| s__Coprococcus_comes | rs10987256, rs11889243, rs12584973, rs12872336, rs17106044, rs17138993, rs6772892, rs6796475, rs75781806, |
| s__Escherichia_unclassified | rs10900435, rs112644714, rs115534005, rs12300342, rs12700345, rs2791437, rs35630916, |
| s__Ruminococcus_lactaris | rs10858895, rs1878735, rs4245954, rs600038, rs7148318, rs72698095, rs7610129, rs7851638, |
| s__Bacteroides_faecis | rs2690011, rs7526994, rs7648378, rs79975503, |
| s__Bacteroides_uniformis | rs6694880, |
| s__Clostridium_asparagiforme | rs35665752, |
| s__Bifidobacterium_catenulatum | rs35948646, rs79651411, rs9451310, rs6953241, rs76993859, rs12580899, rs80133499, |
| s__Bacteroides_coprocola | rs274124, rs12078845, rs17141635, rs116667913, |
| s__Bacteroides_plebeius | rs2432926, rs7070312, rs12578852, rs10145379, rs1828518, rs34375501, rs28681338, |
| s__Bacteroides_thetaiotaomicron | rs4405810, rs77761288, rs72833501, rs74500162, rs1811207, rs10144174, rs12938892, rs62199113, |
| s__Barnesiella_intestinihominis | rs78335793, rs62135856, rs67396441, rs2132254, rs115004061, rs9884588, rs10026787, rs6920861, rs74448867, rs700801, rs8029854, rs1078988, rs34437635, |
| s__Paraprevotella_clara | rs11678259, rs113022358, rs6748817, rs13429913, rs4459949, rs56353270, rs9462447, rs2027516, rs9397388, rs12686124, rs1305081, rs57091306, rs73415022, |
| s__Alistipes_finegoldii | rs12028677, rs57119184, rs12192901, rs60475061, rs67032605, rs10829629, rs7962165, rs13381124, rs10407049, rs111585182, |
| s__Dorea_unclassified | rs1163447, rs73883469, rs112541720, rs113902057, rs13203138, rs3107045, rs11592751, rs4633561, rs10141037, rs6564810, rs62221575, |
| s__Lachnospiraceae_bacterium_3_1_46FAA | rs4494791, rs61945642, rs11058405, rs7984826, |
| s__Lachnospiraceae_bacterium_7_1_58FAA | rs116114211, rs6757314, rs4691694, rs28640142, rs66476925, rs2527213, rs77141557, rs10773460, rs4300413, rs139469000, rs12977498, |
| s__Roseburia_hominis | rs7590468, rs35004779, rs7786467, rs215996, rs2225380, rs722241, rs854661, |
| s__Subdoligranulum_unclassified | rs12622085, rs1850270, rs123043, rs6830068, rs11726764, rs4721723, rs7000832, rs7104786, rs10836532, rs2105601, rs73044678, rs2317917, rs225280, rs4816989, |
| f__Micrococcaceae | rs616801, rs17049177, rs709159, rs9268614, rs80010435, rs888195, |
| .f__Bifidobacteriaceae | rs1546579, rs182549, rs74452406, rs11732081, rs4859454, rs4472164, rs12657170, rs10455532, rs77432108, rs61939486, rs10873640, rs34823013, |
| f__Coriobacteriaceae | rs77502228, rs6793441, rs28806584, rs4234207, rs2907732, rs78362376, rs7831606, rs550057, rs11652058, rs2849714, |
| f__Bacteroidaceae | rs2774282, rs742682, rs28414316, rs62365577, rs73046099, rs56178001, rs80306758, rs4278144, rs680138, rs2571, rs505128, rs71446723, rs61510729, rs9564193, |
| f__Bacteroidales_noname | rs2465095, rs3106091, rs4294998, rs55712042, rs2700958, rs12449080, rs2008778, rs12602625, rs35445518, |
| f__Porphyromonadaceae | rs41265179, rs10166236, rs1452703, rs13070029, rs1363927, rs12673975, rs11979513, rs9694714, rs2236547, rs366032, rs56238206, rs2448269, rs3863318, rs9542263, rs12456364, rs1109499, rs113710653, rs5767121, |
| f__Prevotellaceae | rs1510611, rs73163971, rs113315281, rs26702, rs4301441, rs7862521, rs4432059, rs55994980, |
| f__Rikenellaceae | rs4949447, rs11211406, rs952822, rs73344869, rs2465506, rs61881300, rs74459573, rs77318370, rs9511112, rs6131687, |
| f__Lactobacillaceae | rs6684536, rs11689862, rs3135176, rs112241999, rs76832256, rs278502, rs12204031, rs1149732, rs2598336, rs17457331, rs9559303, rs2899931, rs2332915, |
| f__Streptococcaceae | rs9828991, rs28719561, rs11153641, rs13235500, rs73447940, rs2610946, rs75955305, rs10869688, rs41294029, rs73242161, rs2668320, rs72679838, rs74064457, rs1901115, rs3892326, |
| f__Clostridiaceae | rs1572504, rs28577018, rs11969660, rs10106275, rs55834012, rs4924450, rs111604729, rs12923305, |
| f__Clostridiales_noname | rs315026, rs12039057, rs36131910, rs74564383, rs72896141, rs35903463, rs7402939, rs4939587, |
| f__Eubacteriaceae | rs1942648, rs17218256, rs3898456, rs4113953, rs9600403, rs2252668, rs6021384, |
| f__Lachnospiraceae | rs11210592, rs11892661, rs12619063, rs9882102, rs116656500, rs2903645, rs166135, rs61025394, rs6558174, rs7078960, rs1892388, rs11625794, |
| f__Oscillospiraceae | rs56001939, rs13119423, rs55682375, rs10769221, rs12446334, rs7255582, rs34865636, rs11087270, |
| f__Ruminococcaceae | rs4446400, rs154221, rs6887055, rs111854245, rs17206746, rs74949229, rs117996720, |
| f__Erysipelotrichaceae | rs78685125, rs4975752, rs733330, rs75298326, rs11652559, rs62131327, rs73037929, rs73114746, |
| f__Acidaminococcaceae | rs76411331, rs7797421, rs7787688, rs55751855, rs2303658, rs11850002, rs67391746, |
| o__Selenomonadales.f__Veillonellaceae | rs11804833, rs6701211, rs62165977, rs258816, rs7746214, rs35025485, rs2347496, rs1882135, rs62024270, rs28393833, rs6105994, rs2313533, |
| f__Burkholderiales_noname | rs6664382, rs9872584, rs1860691, rs4717625, rs13257050, rs11144350, rs73653217, rs1533824, rs1792325, rs10895784, rs16923154, rs10507465, rs12941005, |
| f__Oxalobacteraceae | rs12725104, rs1861231, rs10865362, rs35921003, rs11068499, rs4889373, rs17562516, rs3745098, rs73918563, |
| .f__Sutterellaceae | rs2004833, rs11113910, rs59033852, rs76883601, rs1420536, rs9673456, rs74034215, rs28517505, rs2834333, |
| f__Desulfovibrionaceae | rs2990682, rs73966738, rs10087056, rs11219805, rs237461, |
| f__Enterobacteriaceae | rs113117201, rs13138313, rs13166339, rs115534005, rs112572980, |
| f__Pasteurellaceae | rs12254965, rs7132825, rs56162052, |
| f__Verrucomicrobiaceae | rs12710731, rs4670221, rs6823767, rs68058313, rs1298928, rs9368149, rs36119306, rs7912369, rs4075877, rs166570, rs55926131, rs35895642, rs1532932, rs79041579, rs2523176, |
| o__Actinomycetales | rs13236023, rs888195, rs7334276, |
| o__Bifidobacteriales | rs1546579, rs182549, rs74452406, rs11732081, rs4859454, rs4472164, rs12657170, rs10455532, rs77432108, rs61939486, rs10873640, rs34823013, |
| o__Coriobacteriales | rs77502228, rs6793441, rs28806584, rs4234207, rs2907732, rs78362376, rs7831606, rs550057, rs11652058, rs2849714, |
| c__Bacteroidia.o__Bacteroidales | rs2807619, rs10158939, rs1023692, rs9843097, rs2603095, rs73098732, rs13283363, rs74949229, rs10836532, rs28538857, rs11657406, rs783253, rs2698177, |
| c__Bacilli.o__Lactobacillales | rs12064418, rs75005855, rs11694441, rs76752365, rs17052168, rs7703260, rs946119, rs1960416, rs77655905, rs12889801, rs1400577, rs3892326, |
| o__Clostridiales | rs1023692, rs7794348, rs674292, rs12675379, rs74949229, rs34566098, rs10766557, |
| o__Erysipelotrichales | rs78685125, rs4975752, rs733330, rs75298326, rs11652559, rs62131327, rs73037929, rs73114746, |
| o__Selenomonadales | rs12472147, rs176564, rs10736970, rs78791321, rs77764206, rs2282037, rs12979658, |
| c__Betaproteobacteria.o__Burkholderiales | rs12734494, rs9428844, rs62351959, rs12646537, rs4711841, rs4895929, rs59033852, rs11626416, rs78026425, rs9673456, rs79598028, rs74034215, rs2834333, rs713716, |
| o__Desulfovibrionales | rs2990682, rs73966738, rs10087056, rs11219805, rs237461, |
| o__Enterobacteriales | rs113117201, rs13138313, rs13166339, rs115534005, rs112572980, |
| o__Pasteurellales | rs12254965, rs7132825, rs56162052, |
| o__Verrucomicrobiales | rs12710731, rs4670221, rs6823767, rs68058313, rs1298928, rs9368149, rs36119306, rs7912369, rs4075877, rs166570, rs55926131, rs35895642, rs1532932, rs79041579, rs2523176, |
| p__Actinobacteria | rs182549, rs74452406, rs795981, rs62347983, rs318801, rs35344390, rs2197344, rs61939486, rs926010, rs12955365, rs6044857, |
| k__Bacteria.p__Bacteroidetes | rs2807619, rs10158939, rs1023692, rs9843097, rs2603095, rs73098732, rs13283363, rs74949229, rs10836532, rs28538857, rs11657406, rs783253, rs2698177, |
| p__Firmicutes | rs1023692, rs11940931, rs7794348, rs74949229, rs34566098, rs10833110, |
| k__Bacteria.p__Proteobacteria | rs857127, rs2004833, rs1447497, rs73232724, rs68010592, rs79965903, rs12661131, rs10943378, rs12598268, rs28431837, rs35223586, |
| p__Verrucomicrobia | rs12710731, rs4670221, rs6823767, rs68058313, rs1298928, rs9368149, rs36119306, rs7912369, rs4075877, rs166570, rs55926131, rs35895642, rs1532932, rs79041579, rs2523176, |
| s__Bacteroides_stercoris | rs11125607, rs9809140, rs78677736, rs73812271, rs1549206, rs12110522, rs10101109, rs3003597, |
| s__Bacteroides_xylanisolvens | rs17565877, rs1137722, rs7380390, rs4869373, rs463212, rs111452233, rs4733774, rs11522665, rs12416788, rs10862904, rs12443593, rs2302675, rs34558519, rs117675525, |
| s__Alistipes_putredinis | rs143528380, rs6459977, rs3118528, rs874372, rs2241820, rs6489044, rs77715803, |

**Table S4. *F* statistic of IVs in Mendelian randomization analysis.**

| **Outcome** | **Exposure** | **F** |
| --- | --- | --- |
| BCR | COLANSYN.PWY:colanic.acid.building.blocks.biosynthesis | 477.226 |
| BC | COLANSYN.PWY:colanic.acid.building.blocks.biosynthesis | 477.226 |
| CR | COLANSYN.PWY:colanic.acid.building.blocks.biosynthesis | 477.226 |
| BC | PWY.3781:aerobic.respiration.I:cytochrome.c. | 476.671 |
| CR | PWY.2942:L.lysine.biosynthesis.III | 462.124 |
| BCR | s__Bifidobacterium_adolescentis | 459.825 |
| BC | s__Bifidobacterium_adolescentis | 459.825 |
| CR | s__Bifidobacterium_adolescentis | 459.825 |
| BR | g__Bifidobacterium | 458.111 |
| CRC | P122.PWY:heterolactic.fermentation | 449.136 |
| BC | p__Proteobacteria.c__Gammaproteobacteria | 447.320 |
| CRC | p__Proteobacteria.c__Gammaproteobacteria | 447.320 |
| CC | p__Proteobacteria.c__Gammaproteobacteria | 447.320 |
| CC | ENTBACSYN.PWY:enterobactin.biosynthesis | 443.601 |
| BCR | s__Bacteroides_ovatus | 443.169 |
| BC | s__Bacteroides_ovatus | 443.169 |
| BR | s__Bacteroides_ovatus | 443.169 |
| CC | s__Bacteroides_ovatus | 443.169 |
| CRC | PEPTIDOGLYCANSYN.PWY:peptidoglycan.biosynthesis.I:meso.diaminopimelate.containing. | 442.267 |
| CC | PEPTIDOGLYCANSYN.PWY:peptidoglycan.biosynthesis.I:meso.diaminopimelate.containing. | 442.267 |
| CR | THRESYN.PWY:superpathway.of.L.threonine.biosynthesis | 442.255 |
| BR | PWY.7400:L.arginine.biosynthesis.IV:archaebacteria. | 441.867 |
| CR | PWY.7400:L.arginine.biosynthesis.IV:archaebacteria. | 441.867 |
| BR | c__Negativicutes.o__Selenomonadales | 441.441 |
| BR | p__Firmicutes.c__Negativicutes | 441.437 |
| CRC | NONMEVIPP.PWY:methylerythritol.phosphate.pathway.I | 441.412 |
| CC | X1CMET2.PWY:N10.formyl.tetrahydrofolate.biosynthesis | 441.165 |
| CRC | s__Roseburia_inulinivorans | 441.102 |
| CC | s__Roseburia_inulinivorans | 441.102 |
| BCR | PWY.5989:stearate.biosynthesis.II:bacteria.and.plants. | 441.083 |
| CRC | PWY.6629:superpathway.of.L.tryptophan.biosynthesis | 440.608 |
| CC | PWY.6629:superpathway.of.L.tryptophan.biosynthesis | 440.608 |
| CR | PWY.6629:superpathway.of.L.tryptophan.biosynthesis | 440.608 |
| CC | o__Clostridiales.f__Clostridiaceae | 438.915 |
| BR | PWY.5088:L.glutamate.degradation.VIII:to.propanoate. | 438.768 |
| BCR | c__Bacteroidia.o__Bacteroidales | 438.234 |
| BC | c__Bacteroidia.o__Bacteroidales | 438.234 |
| BCR | p__Bacteroidetes.c__Bacteroidia | 438.214 |
| BC | p__Bacteroidetes.c__Bacteroidia | 438.214 |
| BC | FUC.RHAMCAT.PWY:superpathway.of.fucose.and.rhamnose.degradation | 438.112 |
| BCR | k__Bacteria.p__Bacteroidetes | 438.095 |
| BC | k__Bacteria.p__Bacteroidetes | 438.095 |
| CRC | ARGORNPROST.PWY:arginine:ornithine.and.proline.interconversion | 437.993 |
| CC | ARGORNPROST.PWY:arginine:ornithine.and.proline.interconversion | 437.993 |
| BR | o__Burkholderiales.f__Sutterellaceae | 437.964 |
| CRC | o__Burkholderiales.f__Sutterellaceae | 437.964 |
| CC | o__Burkholderiales.f__Sutterellaceae | 437.964 |
| BR | PWY.6628:superpathway.of.L.phenylalanine.biosynthesis | 437.875 |
| BCR | PWY.6471:peptidoglycan.biosynthesis.IV:Enterococcus.faecium. | 437.425 |
| BR | PWY.6471:peptidoglycan.biosynthesis.IV:Enterococcus.faecium. | 437.425 |
| BC | HISDEG.PWY:L.histidine.degradation.I | 437.370 |
| BC | s__Bifidobacterium_longum | 437.199 |
| BR | o__Bacteroidales.f__Prevotellaceae | 437.124 |
| CC | PWY_REDCITCYC:TCA.cycle.VIII:helicobacter. | 436.667 |
| BC | PWY.7371:1.4.dihydroxy.6.naphthoate.biosynthesis.II | 436.540 |
| BCR | KDO.NAGLIPASYN.PWY:superpathway.of:Kdo.2.lipid.A.biosynthesis | 436.515 |
| CC | g__Rothia | 436.477 |
| BC | PWY.5104:L.isoleucine.biosynthesis.IV | 436.279 |
| CR | PWY.5104:L.isoleucine.biosynthesis.IV | 436.279 |
| BC | PWY.7456:mannan.degradation | 435.955 |
| CC | PWY0.1297:superpathway.of.purine.deoxyribonucleosides.degradation | 435.704 |
| CRC | PWY.7237:myo:.chiro:and.scillo.inositol.degradation | 435.555 |
| CRC | SO4ASSIM.PWY:sulfate.reduction.I:assimilatory. | 435.362 |
| CC | SO4ASSIM.PWY:sulfate.reduction.I:assimilatory. | 435.362 |
| CR | SO4ASSIM.PWY:sulfate.reduction.I:assimilatory. | 435.362 |
| BCR | g__Butyrivibrio | 435.335 |
| CRC | s__Parabacteroides_distasonis | 435.205 |
| CR | s__Parabacteroides_distasonis | 435.205 |
| BCR | PWY.7209:superpathway.of.pyrimidine.ribonucleosides.degradation | 435.051 |
| BC | PWY.7209:superpathway.of.pyrimidine.ribonucleosides.degradation | 435.051 |
| BR | PWY.7209:superpathway.of.pyrimidine.ribonucleosides.degradation | 435.051 |
| CR | s__Roseburia_hominis | 434.868 |
| CC | g__Clostridium | 434.731 |
| CRC | DTDPRHAMSYN.PWY:dTDP.L.rhamnose.biosynthesis.I | 434.471 |
| CC | s__Eubacterium_ventriosum | 434.257 |
| BR | PWY66.422:D.galactose.degradation.V:Leloir.pathway. | 433.906 |
| CRC | PWY66.422:D.galactose.degradation.V:Leloir.pathway. | 433.906 |
| CC | GLYCOCAT.PWY:glycogen.degradation.I:bacterial. | 433.895 |
| CRC | HISTSYN.PWY:L.histidine.biosynthesis | 433.759 |
| CR | g__Dorea | 433.537 |
| CC | o__Actinomycetales.f__Micrococcaceae | 433.433 |
| BR | PWY.4242:pantothenate.and.coenzyme.A.biosynthesis.III | 433.413 |
| BCR | g__Desulfovibrio | 433.226 |
| CR | g__Desulfovibrio | 433.226 |
| BC | s__Bacteroides_finegoldii | 433.183 |
| CRC | PWY.6630:superpathway.of.L.tyrosine.biosynthesis | 432.767 |
| BC | NONOXIPENT.PWY:pentose.phosphate.pathway:non.oxidative.branch. | 432.765 |
| CRC | NONOXIPENT.PWY:pentose.phosphate.pathway:non.oxidative.branch. | 432.765 |
| BR | o__Selenomonadales.f__Veillonellaceae | 432.611 |
| CC | PWY.6507:4.deoxy.L.threo.hex.4.enopyranuronate.degradation | 432.484 |
| BCR | PWY.5913:TCA.cycle.VI:obligate.autotrophs. | 432.400 |
| BC | PWY.5913:TCA.cycle.VI:obligate.autotrophs. | 432.400 |
| BCR | PWY.6823:molybdenum.cofactor.biosynthesis | 432.388 |
| CR | s__Oscillibacter_unclassified | 432.380 |
| BCR | s__Streptococcus_parasanguinis | 432.348 |
| BC | s__Streptococcus_parasanguinis | 432.348 |
| CC | s__Streptococcus_parasanguinis | 432.348 |
| CRC | g__Streptococcus | 432.304 |
| CC | g__Streptococcus | 432.304 |
| CR | PWY.5505:L.glutamate.and.L.glutamine.biosynthesis | 432.225 |
| BR | s__Eubacterium_hallii | 432.219 |
| CRC | PWY.6608:guanosine.nucleotides.degradation.III | 432.156 |
| CR | PWY.6608:guanosine.nucleotides.degradation.III | 432.156 |
| CR | o__Clostridiales.f__Oscillospiraceae | 432.082 |
| CR | PENTOSE.P.PWY:pentose.phosphate.pathway | 431.974 |
| CR | s__Bacteroides_xylanisolvens | 431.854 |
| BCR | PWY.7219:adenosine.ribonucleotides.de.novo.biosynthesis | 431.465 |
| BC | PWY.7219:adenosine.ribonucleotides.de.novo.biosynthesis | 431.465 |
| CRC | PWY.2941:L.lysine.biosynthesis.II | 431.106 |
| CC | PWY.2941:L.lysine.biosynthesis.II | 431.106 |
| BR | CRNFORCAT.PWY:creatinine.degradation.I | 431.001 |
| CC | CRNFORCAT.PWY:creatinine.degradation.I | 431.001 |
| CR | s__Coprococcus_sp_ART55_1 | 430.963 |
| BR | HSERMETANA.PWY:L.methionine.biosynthesis.III | 430.955 |
| CC | HSERMETANA.PWY:L.methionine.biosynthesis.III | 430.955 |
| BCR | GALACT.GLUCUROCAT.PWY:superpathway.of.hexuronide.and.hexuronate.degradation | 430.942 |
| CC | p__Proteobacteria.c__Betaproteobacteria | 430.859 |
| BR | PWY.5097:L.lysine.biosynthesis.VI | 430.666 |
| CC | PWY.5097:L.lysine.biosynthesis.VI | 430.666 |
| CC | s__Rothia_mucilaginosa | 430.507 |
| BCR | g__Subdoligranulum | 430.296 |
| BC | g__Barnesiella | 430.242 |
| CR | g__Barnesiella | 430.242 |
| CC | COBALSYN.PWY:adenosylcobalamin.salvage.from.cobinamide.I | 430.208 |
| CC | PWY0.1296:purine.ribonucleosides.degradation | 430.177 |
| CRC | s__Streptococcus_thermophilus | 429.975 |
| CC | s__Streptococcus_thermophilus | 429.975 |
| CR | s__Streptococcus_thermophilus | 429.975 |
| BR | HOMOSER.METSYN.PWY:L.methionine.biosynthesis.I | 429.892 |
| BC | g__Faecalibacterium | 429.624 |
| BC | PWY.6731:starch.degradation.III | 429.590 |
| BR | PWY_HEME.BIOSYNTHESIS.II:heme.biosynthesis.I:aerobic. | 429.548 |
| BC | c__Betaproteobacteria.o__Burkholderiales | 429.546 |
| CR | AST.PWY:L.arginine.degradation.II:AST.pathway. | 429.445 |
| BC | s__Faecalibacterium_prausnitzii | 429.291 |
| BCR | s__Subdoligranulum_unclassified | 429.214 |
| BCR | s__Pseudoflavonifractor_capillosus | 429.041 |
| BC | s__Pseudoflavonifractor_capillosus | 429.041 |
| CRC | s__Pseudoflavonifractor_capillosus | 429.041 |
| BCR | TRPSYN.PWY:L.tryptophan.biosynthesis | 429.010 |
| BC | TRPSYN.PWY:L.tryptophan.biosynthesis | 429.010 |
| BCR | g__Alistipes | 428.958 |
| CR | PHOSLIPSYN.PWY:superpathway.of.phospholipid.biosynthesis.I:bacteria. | 428.890 |
| BCR | o__Bacteroidales.f__Rikenellaceae | 428.678 |
| CRC | P23.PWY:reductive.TCA.cycle.I | 428.633 |
| BR | PWY.6125:superpathway.of.guanosine.nucleotides.de.novo.biosynthesis.II | 428.566 |
| CC | PWY.6125:superpathway.of.guanosine.nucleotides.de.novo.biosynthesis.II | 428.566 |
| BCR | s__Holdemania_filiformis | 428.517 |
| BR | s__Holdemania_filiformis | 428.517 |
| BC | PWY.5022:4.aminobutanoate.degradation.V | 428.327 |
| CRC | s__Parabacteroides_goldsteinii | 427.910 |
| CC | s__Parabacteroides_goldsteinii | 427.910 |
| CR | PWY.7198:pyrimidine.deoxyribonucleotides.de.novo.biosynthesis.IV | 427.850 |
| BCR | PWY.5838:superpathway.of.menaquinol.8.biosynthesis.I | 427.633 |
| BR | PWY.6545:pyrimidine.deoxyribonucleotides.de.novo.biosynthesis.III | 427.613 |
| BC | P42.PWY:incomplete.reductive.TCA.cycle | 427.600 |
| BR | PWY.5971:palmitate.biosynthesis.II:bacteria.and.plants. | 427.517 |
| CC | PWY.5971:palmitate.biosynthesis.II:bacteria.and.plants. | 427.517 |
| CC | s__Roseburia_intestinalis | 427.503 |
| CC | PWY0.1241:ADP.L.glycero:beta:D.manno.heptose.biosynthesis | 427.446 |
| CR | s__Dorea_longicatena | 427.303 |
| BCR | g__Pseudoflavonifractor | 427.077 |
| BC | g__Pseudoflavonifractor | 427.077 |
| CRC | g__Pseudoflavonifractor | 427.077 |
| CC | PWY.724:superpathway.of.L.lysine:L.threonine.and.L.methionine.biosynthesis.II | 427.041 |
| BR | s__Bacteroides_clarus | 426.969 |
| BCR | g__Gordonibacter | 426.906 |
| BR | g__Gordonibacter | 426.906 |
| CC | o__Lactobacillales.f__Streptococcaceae | 426.892 |
| BCR | s__Gordonibacter_pamelaeae | 426.595 |
| BR | s__Gordonibacter_pamelaeae | 426.595 |
| BCR | PWY0.781:aspartate.superpathway | 426.555 |
| BR | PWY0.781:aspartate.superpathway | 426.555 |
| BR | PWY.7196:superpathway.of.pyrimidine.ribonucleosides.salvage | 426.483 |
| CR | PWY.7196:superpathway.of.pyrimidine.ribonucleosides.salvage | 426.483 |
| CC | s__Bacteroides_faecis | 426.434 |
| BCR | g__Coprococcus | 426.329 |
| BC | g__Coprococcus | 426.329 |
| BR | g__Coprococcus | 426.329 |
| BCR | COA.PWY:coenzyme.A.biosynthesis.I | 426.319 |
| BC | COA.PWY:coenzyme.A.biosynthesis.I | 426.319 |
| CC | COA.PWY:coenzyme.A.biosynthesis.I | 426.319 |
| CRC | s__Sutterella_wadsworthensis | 426.284 |
| CC | s__Sutterella_wadsworthensis | 426.284 |
| CR | GLYCOL.GLYOXDEG.PWY:superpathway.of.glycol.metabolism.and.degradation | 426.126 |
| CR | s__Bacteroides_eggerthii | 426.043 |
| CRC | FAO.PWY:fatty.acid:beta:oxidation.I | 425.682 |
| CC | s__Paraprevotella_xylaniphila | 425.682 |
| BR | g__Odoribacter | 425.670 |
| CR | PWY.7392:taxadiene.biosynthesis:engineered. | 425.623 |
| CR | PWY.7323:superpathway.of.GDP.mannose.derived.O.antigen.building.blocks.biosynthesis | 425.525 |
| BCR | PWY_GLYOXYLATE.BYPASS:glyoxylate.cycle | 425.321 |
| BCR | o__Bacteroidales.f__Bacteroidaceae | 425.070 |
| BCR | GLUCARDEG.PWY:D.glucarate.degradation.I | 425.055 |
| BC | GLUCARDEG.PWY:D.glucarate.degradation.I | 425.055 |
| CC | GLUCARDEG.PWY:D.glucarate.degradation.I | 425.055 |
| BCR | s__Bilophila_wadsworthia | 424.851 |
| BR | s__Bilophila_wadsworthia | 424.851 |
| BCR | BRANCHED.CHAIN.AA.SYN.PWY:superpathway.of.branched.amino.acid.biosynthesis | 424.800 |
| BC | BRANCHED.CHAIN.AA.SYN.PWY:superpathway.of.branched.amino.acid.biosynthesis | 424.800 |
| BR | s__Eubacterium_rectale | 424.712 |
| CRC | g__Flavonifractor | 424.581 |
| CC | g__Flavonifractor | 424.581 |
| CC | o__Clostridiales.f__Lachnospiraceae | 424.561 |
| CC | p__Firmicutes.c__Bacilli | 424.344 |
| BCR | PWY.6284:superpathway.of.unsaturated.fatty.acids.biosynthesis:E:coli | 424.309 |
| BC | PWY.6284:superpathway.of.unsaturated.fatty.acids.biosynthesis:E:coli | 424.309 |
| BCR | o__Selenomonadales.f__Acidaminococcaceae | 424.295 |
| BR | o__Selenomonadales.f__Acidaminococcaceae | 424.295 |
| CC | P162.PWY:L.glutamate.degradation.V:via.hydroxyglutarate. | 424.288 |
| CR | s__Veillonella_unclassified | 424.042 |
| BCR | g__Bacteroides | 424.014 |
| CRC | PWY0.1479:tRNA.processing | 423.966 |
| CR | PWY0.1479:tRNA.processing | 423.966 |
| BCR | P461.PWY:hexitol.fermentation.to.lactate:formate:ethanol.and.acetate | 423.715 |
| CC | PWY.6519:8.amino.7.oxononanoate.biosynthesis.I | 423.403 |
| CR | PWY.6519:8.amino.7.oxononanoate.biosynthesis.I | 423.403 |
| CC | s__Flavonifractor_plautii | 423.162 |
| CRC | PWY.6263:superpathway.of.menaquinol.8.biosynthesis.II | 423.005 |
| CC | PWY.6263:superpathway.of.menaquinol.8.biosynthesis.II | 423.005 |
| BR | PWY.5686:UMP.biosynthesis | 422.958 |
| CRC | g__Sutterella | 422.711 |
| CC | g__Sutterella | 422.711 |
| BR | g__Lactobacillus | 422.581 |
| BC | PWY0.1298:superpathway.of.pyrimidine.deoxyribonucleosides.degradation | 422.422 |
| CC | PWY0.1298:superpathway.of.pyrimidine.deoxyribonucleosides.degradation | 422.422 |
| CRC | g__Parabacteroides | 422.412 |
| CR | g__Parabacteroides | 422.412 |
| CRC | s__Lactobacillus_delbrueckii | 422.331 |
| CC | s__Lactobacillus_delbrueckii | 422.331 |
| BC | PWY4FS.7:phosphatidylglycerol.biosynthesis.I:plastidic. | 422.234 |
| CC | s__Desulfovibrio_piger | 422.001 |
| BCR | s__Bifidobacterium_catenulatum | 421.916 |
| CRC | o__Erysipelotrichales.f__Erysipelotrichaceae | 421.799 |
| CR | o__Erysipelotrichales.f__Erysipelotrichaceae | 421.799 |
| CRC | c__Erysipelotrichia.o__Erysipelotrichales | 421.786 |
| CR | c__Erysipelotrichia.o__Erysipelotrichales | 421.786 |
| BCR | PWY.5189:tetrapyrrole.biosynthesis.II:from.glycine. | 421.780 |
| CRC | p__Firmicutes.c__Erysipelotrichia | 421.762 |
| CR | p__Firmicutes.c__Erysipelotrichia | 421.762 |
| BR | o__Lactobacillales.f__Lactobacillaceae | 421.719 |
| BR | g__Sutterellaceae_unclassified | 421.539 |
| BR | PWY.5100:pyruvate.fermentation.to.acetate.and.lactate.II | 421.458 |
| BR | s__Lachnospiraceae_bacterium_3_1_46FAA | 421.198 |
| BR | s__Prevotella_copri | 421.085 |
| CC | s__Prevotella_copri | 421.085 |
| BCR | PANTOSYN.PWY:pantothenate.and.coenzyme.A.biosynthesis.I | 421.013 |
| BC | PANTOSYN.PWY:pantothenate.and.coenzyme.A.biosynthesis.I | 421.013 |
| CR | c__Gammaproteobacteria.o__Pasteurellales | 420.951 |
| CC | s__Holdemania_unclassified | 420.939 |
| CR | o__Pasteurellales.f__Pasteurellaceae | 420.894 |
| BCR | PWY.6897:thiamin.salvage.II | 420.189 |
| CRC | s__Eubacterium_siraeum | 419.934 |
| CR | s__Eubacterium_siraeum | 419.934 |
| BCR | PWY.6123:inosine.5:phosphate.biosynthesis.I | 419.121 |
| CRC | PWY.6123:inosine.5:phosphate.biosynthesis.I | 419.121 |
| CR | PWY.6690:cinnamate.and.3.hydroxycinnamate.degradation.to.2.oxopent.4.enoate | 418.894 |
| BR | s__Coprococcus_catus | 418.633 |
| CRC | s__Coprococcus_catus | 418.633 |
| CR | s__Coprococcus_catus | 418.633 |
| BR | POLYAMSYN.PWY:superpathway.of.polyamine.biosynthesis.I | 417.846 |
| CRC | UBISYN.PWY:superpathway.of.ubiquinol.8.biosynthesis:prokaryotic. | 417.452 |
| BC | s__Lachnospiraceae_bacterium_5_1_63FAA | 417.274 |
| BCR | PWY.4984:urea.cycle | 413.987 |
| BC | PWY.4984:urea.cycle | 413.987 |
| CRC | PWY.4984:urea.cycle | 413.987 |
| CC | PWY.4984:urea.cycle | 413.987 |
| CRC | s__Lachnospiraceae_bacterium_1_1_57FAA | 410.731 |
| CR | s__Lachnospiraceae_bacterium_1_1_57FAA | 410.731 |

*Note:* CRC: colorectal cancer; CC: malignant tumors of colon; CR: malignant tumors of rectum; BCR: benign tumors of colorectum; BC: benign tumors of colon; BR: benign tumors of rectum.

**Table S5. Results of sensitivity analysis in Mendelian randomization analysis.**

| **Outcome** | | **Exposure** | **Method** | **nsnp** | **Q** | **Q_p** | **egger_p** | **Presso_p** |
| --- | --- | --- | --- | --- | --- | --- | --- | --- |
| CC | ARGORNPROST.PWY:arginine:ornithine.and.proline.interconversion | | IVW(M) | 7 | 1.48 | 0.92 | 0.85 | 0.98 |
| CC | c__Bacilli | | MR Egger | 13 | 6.95 | 0.80 | 0.03 | 0.45 |
| BCR | c__Bacteroidia | | MR Egger | 13 | 7.69 | 0.74 | 0.03 | 0.39 |
| BC | c__Bacteroidia | | MR Egger | 13 | 10.16 | 0.52 | 0.03 | 0.24 |
| CC | c__Betaproteobacteria | | IVW(M) | 13 | 8.42 | 0.68 | 0.63 | 0.75 |
| CR | c__Erysipelotrichia | | IVW(M) | 8 | 1.53 | 0.96 | 0.77 | 0.97 |
| CRC | c__Erysipelotrichia | | IVW(M) | 8 | 3.39 | 0.76 | 0.83 | 0.83 |
| BC | c__Gammaproteobacteria | | IVW(M) | 4 | 0.34 | 0.84 | 0.60 | 0.91 |
| CRC | c__Gammaproteobacteria | | IVW(M) | 4 | 0.31 | 0.86 | 0.13 | 0.51 |
| CC | c__Gammaproteobacteria | | IVW(M) | 4 | 2.41 | 0.30 | 0.76 | 0.49 |
| BR | c__Negativicutes | | IVW(M) | 7 | 0.73 | 0.98 | 0.96 | 0.99 |
| CC | COA.PWY:coenzyme.A.biosynthesis.I | | IVW(M) | 9 | 1.22 | 0.99 | 0.56 | 0.98 |
| BC | COLANSYN.PWY:colanic.acid.building.blocks.biosynthesis | | IVW(M) | 2 | 0.21 | 0.65 | NA | NA |
| BCR | f__Acidaminococcaceae | | IVW(M) | 7 | 2.95 | 0.71 | 0.48 | 0.72 |
| BR | f__Acidaminococcaceae | | IVW(M) | 7 | 3.76 | 0.58 | 0.15 | 0.44 |
| BCR | f__Bacteroidaceae | | MR Egger | 13 | 6.11 | 0.87 | 0.02 | 0.46 |
| CC | f__Clostridiaceae | | IVW(M) | 8 | 1.34 | 0.97 | 0.59 | 0.96 |
| CR | f__Erysipelotrichaceae | | IVW(M) | 8 | 1.53 | 0.96 | 0.77 | 0.97 |
| CRC | f__Erysipelotrichaceae | | IVW(M) | 8 | 3.39 | 0.76 | 0.83 | 0.83 |
| CC | f__Lachnospiraceae | | IVW(M) | 11 | 9.03 | 0.43 | 0.17 | 0.38 |
| BR | f__Lactobacillaceae | | IVW(M) | 12 | 13.17 | 0.21 | 0.47 | 0.29 |
| CC | f__Micrococcaceae | | IVW(M) | 6 | 0.68 | 0.95 | 0.68 | 0.98 |
| CR | f__Oscillospiraceae | | IVW(M) | 7 | 1.84 | 0.87 | 0.92 | 0.95 |
| CR | f__Pasteurellaceae | | IVW(M) | 3 | 0.46 | 0.50 | 0.97 | NA |
| BR | f__Prevotellaceae | | IVW(M) | 8 | 2.14 | 0.91 | 0.91 | 0.94 |
| BCR | f__Rikenellaceae | | IVW(M) | 10 | 1.78 | 0.99 | 0.56 | 0.98 |
| CC | f__Streptococcaceae | | IVW(M) | 13 | 2.52 | 1.00 | 0.99 | 1.00 |
| BR | f__Sutterellaceae | | IVW(M) | 8 | 2.73 | 0.84 | 0.76 | 0.89 |
| CRC | f__Sutterellaceae | | MR Egger | 8 | 7.74 | 0.26 | 0.00 | 0.01 |
| CC | f__Sutterellaceae | | MR Egger | 8 | 4.86 | 0.56 | 0.02 | 0.24 |
| BR | f__Veillonellaceae | | IVW(M) | 11 | 6.70 | 0.67 | 0.92 | 0.75 |
| BCR | g__Alistipes | | IVW(M) | 10 | 1.76 | 0.99 | 0.55 | 0.98 |
| BCR | g__Bacteroides | | MR Egger | 14 | 6.88 | 0.87 | 0.02 | 0.54 |
| BC | g__Barnesiella | | IVW(M) | 14 | 8.08 | 0.78 | 0.72 | 0.82 |
| CR | g__Barnesiella | | MR Egger | 14 | 10.83 | 0.54 | 0.03 | 0.26 |
| BR | g__Bifidobacterium | | IVW(M) | 10 | 4.26 | 0.83 | 0.86 | 0.88 |
| BCR | g__Butyrivibrio | | IVW(M) | 10 | 6.54 | 0.59 | 0.43 | 0.70 |
| CC | g__Clostridium | | IVW(M) | 9 | 1.35 | 0.99 | 0.54 | 0.98 |
| BCR | g__Coprococcus | | MR Egger | 10 | 6.51 | 0.59 | 0.01 | 0.16 |
| BC | g__Coprococcus | | MR Egger | 10 | 5.14 | 0.74 | 0.02 | 0.28 |
| BR | g__Coprococcus | | MR Egger | 10 | 2.74 | 0.95 | 0.00 | 0.28 |
| CR | g__Desulfovibrio | | IVW(M) | 2 | 0.06 | 0.80 | NA | NA |
| BCR | g__Desulfovibrio | | IVW(M) | 2 | 0.17 | 0.68 | NA | NA |
| CR | g__Dorea | | IVW(M) | 5 | 1.50 | 0.68 | 0.87 | 0.87 |
| BC | g__Faecalibacterium | | IVW(M) | 7 | 1.04 | 0.96 | 0.69 | 0.98 |
| CC | g__Flavonifractor | | IVW(M) | 5 | 0.17 | 0.98 | 0.46 | 0.97 |
| CRC | g__Flavonifractor | | IVW(M) | 5 | 1.09 | 0.78 | 0.07 | 0.42 |
| BCR | g__Gordonibacter | | IVW(M) | 4 | 0.01 | 0.99 | 0.85 | 0.99 |
| BR | g__Gordonibacter | | IVW(M) | 4 | 0.05 | 0.98 | 0.22 | 0.64 |
| BR | g__Lactobacillus | | IVW(M) | 11 | 10.40 | 0.32 | 0.38 | 0.35 |
| BR | **g__Odoribacter** | | IVW(M) | 9 | 1.94 | 0.96 | 0.59 | 0.97 |
| CRC | g__Parabacteroides | | MR Egger | 13 | 2.54 | 1.00 | 0.00 | 0.27 |
| CR | g__Parabacteroides | | MR Egger | 13 | 6.29 | 0.85 | 0.00 | 0.10 |
| CRC | g__Pseudoflavonifractor | | IVW(M) | 9 | 6.56 | 0.48 | 0.45 | 0.56 |
| BCR | g__Pseudoflavonifractor | | IVW(M) | 9 | 7.40 | 0.39 | 0.76 | 0.54 |
| BC | g__Pseudoflavonifractor | | IVW(M) | 9 | 3.89 | 0.79 | 0.19 | 0.69 |
| CC | g__Rothia | | IVW(M) | 6 | 0.68 | 0.95 | 0.68 | 0.98 |
| CRC | g__Streptococcus | | IVW(M) | 7 | 4.11 | 0.53 | 0.80 | 0.68 |
| CC | g__Streptococcus | | IVW(M) | 7 | 0.79 | 0.98 | 0.70 | 0.99 |
| BCR | g__Subdoligranulum | | IVW(M) | 13 | 5.96 | 0.88 | 0.86 | 0.92 |
| CRC | g__Sutterella | | IVW(M) | 4 | 1.44 | 0.49 | 0.77 | 0.73 |
| CC | g__Sutterella | | IVW(M) | 4 | 1.64 | 0.44 | 0.79 | 0.70 |
| BR | g__Sutterellaceae_unclassified | | IVW(M) | 6 | 3.63 | 0.46 | 0.27 | 0.45 |
| BCR | GLUCARDEG.PWY:D.glucarate.degradation.I | | IVW(M) | 3 | 0.00 | 0.95 | 0.89 | NA |
| CC | GLUCARDEG.PWY:D.glucarate.degradation.I | | IVW(M) | 3 | 0.09 | 0.77 | 0.69 | NA |
| BC | GLUCARDEG.PWY:D.glucarate.degradation.I | | IVW(M) | 3 | 0.06 | 0.81 | 0.78 | NA |
| CR | GLYCOL.GLYOXDEG.PWY:superpathway.of.glycol.metabolism.and.degradation | | IVW(M) | 11 | 8.84 | 0.45 | 0.55 | 0.53 |
| BR | HOMOSER.METSYN.PWY:L.methionine.biosynthesis.I | | IVW(M) | 8 | 1.12 | 0.98 | 0.35 | 0.94 |
| BR | HSERMETANA.PWY:L.methionine.biosynthesis.III | | IVW(M) | 16 | 11.72 | 0.63 | 0.83 | 0.73 |
| BCR | o__Bacteroidales | | MR Egger | 13 | 7.68 | 0.74 | 0.03 | 0.39 |
| BC | o__Bacteroidales | | MR Egger | 13 | 10.15 | 0.52 | 0.03 | 0.24 |
| BC | o__Burkholderiales | | IVW(M) | 14 | 8.49 | 0.75 | 0.53 | 0.74 |
| CR | o__Erysipelotrichales | | IVW(M) | 8 | 1.53 | 0.96 | 0.77 | 0.97 |
| CRC | o__Erysipelotrichales | | IVW(M) | 8 | 3.39 | 0.76 | 0.83 | 0.83 |
| CR | o__Pasteurellales | | IVW(M) | 3 | 0.46 | 0.50 | 0.97 | NA |
| BR | o__Selenomonadales | | IVW(M) | 7 | 0.73 | 0.98 | 0.96 | 0.99 |
| BCR | p__Bacteroidetes | | MR Egger | 13 | 7.69 | 0.74 | 0.03 | 0.39 |
| BC | p__Bacteroidetes | | MR Egger | 13 | 10.16 | 0.52 | 0.03 | 0.24 |
| CRC | P122.PWY:heterolactic.fermentation | | IVW(M) | 5 | 0.00 | 1.00 | 0.66 | 1.00 |
| BCR | PANTOSYN.PWY:pantothenate.and.coenzyme.A.biosynthesis.I | | IVW(M) | 8 | 3.34 | 0.77 | 0.99 | 0.88 |
| BC | PANTOSYN.PWY:pantothenate.and.coenzyme.A.biosynthesis.I | | IVW(M) | 8 | 4.37 | 0.63 | 0.45 | 0.72 |
| CR | PENTOSE.P.PWY:pentose.phosphate.pathway | | IVW(M) | 12 | 3.00 | 0.98 | 0.72 | 0.99 |
| BCR | PWY.4984:urea.cycle | | IVW(M) | 8 | 3.00 | 0.81 | 0.92 | 0.89 |
| BC | PWY.4984:urea.cycle | | IVW(M) | 8 | 1.42 | 0.96 | 0.68 | 0.98 |
| BR | PWY.5686:UMP.biosynthesis | | IVW(M) | 6 | 1.24 | 0.87 | 0.59 | 0.91 |
| CC | PWY.5971:palmitate.biosynthesis.II:bacteria.and.plants. | | IVW(M) | 11 | 9.34 | 0.41 | 0.18 | 0.36 |
| CRC | PWY.6123:inosine.5:phosphate.biosynthesis.I | | IVW(M) | 6 | 0.79 | 0.94 | 0.47 | 0.93 |
| BCR | PWY.6284:superpathway.of.unsaturated.fatty.acids.biosynthesis:E:coli. | | IVW(M) | 9 | 0.77 | 1.00 | 0.91 | 1.00 |
| BR | PWY.6471:peptidoglycan.biosynthesis.IV:Enterococcus.faecium. | | IVW(M) | 3 | 0.52 | 0.47 | 0.80 | NA |
| CRC | PWY.6608:guanosine.nucleotides.degradation.III | | IVW(M) | 10 | 4.26 | 0.83 | 0.62 | 0.92 |
| CR | PWY.6608:guanosine.nucleotides.degradation.III | | IVW(M) | 10 | 4.52 | 0.81 | 0.19 | 0.79 |
| CR | PWY.6690:cinnamate.and.3.hydroxycinnamate.degradation.to.2.oxopent.4.enoate | | IVW(M) | 8 | 4.01 | 0.67 | 0.54 | 0.75 |
| BC | PWY.7219:adenosine.ribonucleotides.de.novo.biosynthesis | | IVW(M) | 12 | 7.70 | 0.66 | 0.38 | 0.70 |
| BC | PWY.7371:1.4.dihydroxy.6.naphthoate.biosynthesis.II | | IVW(M) | 8 | 5.41 | 0.49 | 0.40 | 0.57 |
| BCR | PWY_GLYOXYLATE.BYPASS:glyoxylate.cycle | | IVW(M) | 11 | 4.80 | 0.85 | 0.51 | 0.88 |
| CC | PWY0.1297:superpathway.of.purine.deoxyribonucleosides.degradation | | IVW(M) | 10 | 4.04 | 0.85 | 0.75 | 0.94 |
| CC | PWY0.1298:superpathway.of.pyrimidine.deoxyribonucleosides.degradation | | IVW(M) | 14 | 10.90 | 0.54 | 0.41 | 0.59 |
| CRC | PWY0.1479:tRNA.processing | | IVW(M) | 9 | 4.35 | 0.74 | 0.91 | 0.86 |
| CR | PWY0.1479:tRNA.processing | | IVW(M) | 9 | 3.97 | 0.78 | 1.00 | 0.88 |
| BC | PWY4FS.7:phosphatidylglycerol.biosynthesis.I:plastidic. | | IVW(M) | 14 | 7.14 | 0.85 | 0.65 | 0.86 |
| BR | s__Bacteroides_clarus | | MR Egger | 9 | 5.85 | 0.56 | 0.01 | 0.15 |
| CR | s__Bacteroides_eggerthii | | IVW(M) | 7 | 3.04 | 0.69 | 0.85 | 0.78 |
| CC | s__Bacteroides_faecis | | IVW(M) | 4 | 0.12 | 0.94 | 0.92 | 0.98 |
| BC | s__Bacteroides_finegoldii | | MR Egger | 15 | 8.09 | 0.84 | 0.01 | 0.34 |
| BCR | s__Bacteroides_ovatus | | IVW(M) | 14 | 11.50 | 0.49 | 0.15 | 0.41 |
| BC | s__Bacteroides_ovatus | | IVW(M) | 14 | 11.76 | 0.47 | 0.18 | 0.40 |
| BR | s__Bacteroides_ovatus | | IVW(M) | 14 | 7.22 | 0.84 | 0.82 | 0.89 |
| CC | s__Bacteroides_ovatus | | MR Egger | 14 | 9.09 | 0.69 | 0.03 | 0.37 |
| CR | s__Bacteroides_xylanisolvens | | IVW(M) | 13 | 5.29 | 0.92 | 0.32 | 0.91 |
| BCR | s__Bifidobacterium_adolescentis | | MR Egger | 10 | 3.44 | 0.90 | 0.00 | 0.21 |
| BC | s__Bifidobacterium_adolescentis | | MR Egger | 10 | 6.21 | 0.62 | 0.00 | 0.11 |
| CR | s__Bifidobacterium_adolescentis | | MR Egger | 10 | 10.21 | 0.25 | 0.02 | 0.03 |
| BCR | s__Bifidobacterium_catenulatum | | IVW(M) | 6 | 0.11 | 1.00 | 0.95 | 1.00 |
| BC | s__Bifidobacterium_longum | | IVW(M) | 11 | 7.99 | 0.53 | 0.57 | 0.61 |
| BCR | s__Bilophila_wadsworthia | | MR Egger | 7 | 4.53 | 0.48 | 0.00 | 0.04 |
| BR | s__Bilophila_wadsworthia | | MR Egger | 7 | 5.71 | 0.34 | 0.00 | 0.01 |
| CRC | **s__Coprococcus_catus** | | IVW(M) | 4 | 1.34 | 0.51 | 0.84 | 0.73 |
| CR | **s__Coprococcus_catus** | | IVW(M) | 4 | 0.22 | 0.90 | 0.86 | 0.96 |
| BR | **s__Coprococcus_catus** | | IVW(M) | 4 | 1.21 | 0.55 | 0.93 | 0.81 |
| CR | s__Coprococcus_sp_ART55_1 | | IVW(M) | 4 | 3.51 | 0.17 | 0.87 | 0.35 |
| CC | **s__Desulfovibrio_piger** | | IVW(M) | 6 | 0.51 | 0.97 | 0.94 | 0.99 |
| CR | s__Dorea_longicatena | | IVW(M) | 9 | 3.10 | 0.88 | 0.92 | 0.94 |
| BR | s__Eubacterium_hallii | | MR Egger | 12 | 12.34 | 0.26 | 0.00 | 0.02 |
| BR | s__Eubacterium_rectale | | IVW(M) | 8 | 0.51 | 1.00 | 0.38 | 0.99 |
| CRC | s__Eubacterium_siraeum | | IVW(M) | 14 | 18.40 | 0.10 | 0.43 | 0.11 |
| CR | s__Eubacterium_siraeum | | IVW(M) | 14 | 16.81 | 0.16 | 0.88 | 0.22 |
| CC | **s__Eubacterium_ventriosum** | | IVW(M) | 6 | 1.91 | 0.75 | 0.78 | 0.84 |
| BC | s__Faecalibacterium_prausnitzii | | IVW(M) | 8 | 1.03 | 0.98 | 0.68 | 0.99 |
| CC | s__Flavonifractor_plautii | | IVW(M) | 6 | 4.57 | 0.33 | 0.68 | 0.54 |
| BCR | **s__Gordonibacter_pamelaeae** | | IVW(M) | 4 | 0.01 | 0.99 | 0.85 | 1.00 |
| BR | **s__Gordonibacter_pamelaeae** | | IVW(M) | 4 | 0.04 | 0.98 | 0.22 | 0.65 |
| BCR | **s__Holdemania_filiformis** | | IVW(M) | 3 | 0.06 | 0.81 | 0.38 | NA |
| BR | **s__Holdemania_filiformis** | | IVW(M) | 3 | 0.00 | 0.99 | 0.80 | NA |
| CC | s__Holdemania_unclassified | | IVW(M) | 10 | 7.55 | 0.48 | 0.78 | 0.62 |
| CRC | s__Lachnospiraceae_bacterium_1_1_57FAA | | Wald ratio | 1 | NA | NA | NA | NA |
| CR | s__Lachnospiraceae_bacterium_1_1_57FAA | | Wald ratio | 1 | NA | NA | NA | NA |
| BC | **s__Lachnospiraceae_bacterium_3_1_46FAA** | | IVW(M) | 5 | 1.34 | 0.72 | 0.07 | 0.42 |
| BR | s__Lachnospiraceae_bacterium_3_1_46FAA | | IVW(M) | 4 | 0.32 | 0.85 | 0.96 | 0.96 |
| CC | s__Lachnospiraceae_bacterium_5_1_63FAA | | IVW(M) | 14 | 12.72 | 0.39 | 0.17 | 0.37 |
| CRC | **s__Lactobacillus_delbrueckii** | | IVW(M) | 14 | 19.87 | 0.07 | 0.35 | 0.06 |
| CR | s__Lactobacillus_delbrueckii | | IVW(M) | 7 | 1.83 | 0.87 | 0.90 | 0.95 |
| CRC | s__Oscillibacter_unclassified | | IVW(M) | 8 | 3.33 | 0.77 | 0.40 | 0.80 |
| CR | s__Parabacteroides_distasonis | | IVW(M) | 8 | 4.65 | 0.59 | 0.36 | 0.66 |
| CRC | s__Parabacteroides_distasonis | | IVW(M) | 7 | 5.93 | 0.31 | 0.95 | 0.45 |
| CC | s__Parabacteroides_goldsteinii | | IVW(M) | 7 | 6.22 | 0.29 | 0.76 | 0.42 |
| CC | s__Paraprevotella_xylaniphila | | IVW(M) | 11 | 3.31 | 0.95 | 0.97 | 0.97 |
| BR | s__Prevotella_copri | | MR Egger | 12 | 11.35 | 0.33 | 0.06 | 0.22 |
| BR | s__Prevotella_copri | | IVW(M) | 12 | 11.35 | 0.33 | 0.06 | 0.22 |
| CC | s__Prevotella_copri | | IVW(M) | 12 | 8.95 | 0.54 | 0.59 | 0.66 |
| BCR | s__Pseudoflavonifractor_capillosus | | IVW(M) | 11 | 6.27 | 0.71 | 0.57 | 0.75 |
| BC | s__Pseudoflavonifractor_capillosus | | IVW(M) | 11 | 3.80 | 0.92 | 0.43 | 0.91 |
| CRC | s__Pseudoflavonifractor_capillosus | | IVW(M) | 11 | 9.58 | 0.39 | 0.73 | 0.50 |
| CR | s__Roseburia_hominis | | IVW(M) | 7 | 1.65 | 0.89 | 0.74 | 0.97 |
| CC | s__Roseburia_intestinalis | | MR Egger | 9 | 9.50 | 0.22 | 0.02 | 0.05 |
| CRC | s__Roseburia_inulinivorans | | IVW(M) | 4 | 0.02 | 0.99 | 0.91 | 1.00 |
| CC | s__Roseburia_inulinivorans | | IVW(M) | 4 | 0.30 | 0.86 | 0.58 | 0.91 |
| CC | s__Rothia_mucilaginosa | | IVW(M) | 4 | 0.12 | 0.94 | 0.78 | 0.96 |
| BC | s__Streptococcus_parasanguinis | | IVW(M) | 3 | 0.00 | 0.98 | 0.61 | NA |
| BCR | s__Streptococcus_parasanguinis | | IVW(M) | 3 | 0.14 | 0.71 | 0.71 | NA |
| CC | s__Streptococcus_parasanguinis | | IVW(M) | 3 | 0.45 | 0.50 | 0.99 | NA |
| CRC | s__Streptococcus_thermophilus | | IVW(M) | 4 | 0.74 | 0.69 | 0.21 | 0.63 |
| CC | s__Streptococcus_thermophilus | | IVW(M) | 4 | 0.63 | 0.73 | 0.42 | 0.72 |
| CR | s__Streptococcus_thermophilus | | IVW(M) | 4 | 3.07 | 0.22 | 0.67 | 0.44 |
| BCR | s__Subdoligranulum_unclassified | | IVW(M) | 12 | 5.90 | 0.82 | 0.80 | 0.91 |
| CRC | s__Sutterella_wadsworthensis | | IVW(M) | 4 | 1.44 | 0.49 | 0.76 | 0.73 |
| CC | s__Sutterella_wadsworthensis | | IVW(M) | 4 | 1.71 | 0.43 | 0.82 | 0.70 |
| CR | s__Veillonella_unclassified | | IVW(M) | 10 | 5.21 | 0.73 | 0.26 | 0.74 |
| CRC | SO4ASSIM.PWY:sulfate.reduction.I:assimilatory. | | IVW(M) | 4 | 0.01 | 1.00 | 0.89 | 1.00 |
| CC | SO4ASSIM.PWY:sulfate.reduction.I:assimilatory. | | IVW(M) | 4 | 0.06 | 0.97 | 0.92 | 1.00 |
| CC | X1CMET2.PWY:N10.formyl.tetrahydrofolate.biosynthesis | | IVW(M) | 9 | 2.16 | 0.95 | 0.57 | 0.94 |

*Note:* CRC: colorectal cancer; CC: malignant tumors of colon; CR: malignant tumors of rectum; BCR: benign tumors of colorectum; BC: benign tumors of colon; BR: benign tumors of rectum; IVW(M), Inverse variance weighted (multiplicative random effects); nsnp is the number of SNPs being used as IVs.

**Table S6. Steiger test results from exposure to outcome.**

| **Exposure** | **Outcome** | **snp_r2.exposure** | **snp_r2.outcome** | **Correct causal direction** | **steiger_p** |
| --- | --- | --- | --- | --- | --- |
| ARGORNPROST.PWY..arginine..ornithine.and.proline.interconversion | CC | 0.02 | 2.15E-05 | TRUE | 7.31E-33 |
| c__Bacilli | CC | 0.04 | 5.41E-05 | TRUE | 4.29E-56 |
| c__Bacteroidia | BC | 0.04 | 6.74E-05 | TRUE | 2.52E-59 |
| c__Bacteroidia | BCR | 0.04 | 6.38E-05 | TRUE | 1.78E-59 |
| c__Betaproteobacteria | CC | 0.04 | 5.49E-05 | TRUE | 9.53E-58 |
| c__Erysipelotrichia | CRC | 0.02 | 2.85E-05 | TRUE | 1.20E-34 |
| c__Erysipelotrichia | CR | 0.02 | 1.56E-05 | TRUE | 2.75E-35 |
| c__Gammaproteobacteria | CC | 0.01 | 2.89E-05 | TRUE | 1.61E-19 |
| c__Gammaproteobacteria | CRC | 0.01 | 2.99E-05 | TRUE | 1.72E-19 |
| c__Gammaproteobacteria | BC | 0.01 | 7.96E-06 | TRUE | 2.01E-20 |
| c__Negativicutes | BR | 0.02 | 2.03E-05 | TRUE | 1.56E-33 |
| COA.PWY..coenzyme.A.biosynthesis.I | CC | 0.02 | 1.69E-05 | TRUE | 2.14E-40 |
| COLANSYN.PWY..colanic.acid.building.blocks.biosynthesis | BC | 0.01 | 1.08E-05 | TRUE | 8.00E-12 |
| f__Acidaminococcaceae | BCR | 0.02 | 3.07E-05 | TRUE | 1.34E-30 |
| f__Acidaminococcaceae | BR | 0.02 | 5.62E-05 | TRUE | 1.01E-29 |
| f__Bacteroidaceae | BCR | 0.04 | 6.39E-05 | TRUE | 6.87E-56 |
| f__Clostridiaceae | CC | 0.02 | 1.41E-05 | TRUE | 3.84E-38 |
| f__Erysipelotrichaceae | CRC | 0.02 | 2.85E-05 | TRUE | 1.18E-34 |
| f__Erysipelotrichaceae | CR | 0.02 | 1.56E-05 | TRUE | 2.71E-35 |
| f__Lachnospiraceae | CC | 0.03 | 6.89E-05 | TRUE | 9.09E-47 |
| f__Lactobacillaceae | BR | 0.03 | 9.09E-05 | TRUE | 9.17E-50 |
| f__Micrococcaceae | CC | 0.02 | 1.09E-05 | TRUE | 3.13E-28 |
| f__Oscillospiraceae | CR | 0.02 | 1.82E-05 | TRUE | 3.43E-32 |
| f__Pasteurellaceae | CR | 0.01 | 6.71E-06 | TRUE | 5.31E-14 |
| f__Prevotellaceae | BR | 0.02 | 2.08E-05 | TRUE | 1.69E-37 |
| f__Rikenellaceae | BCR | 0.03 | 1.43E-05 | TRUE | 8.82E-46 |
| f__Streptococcaceae | CC | 0.04 | 2.09E-05 | TRUE | 2.11E-58 |
| f__Sutterellaceae | CC | 0.02 | 5.15E-05 | TRUE | 2.34E-36 |
| f__Sutterellaceae | CRC | 0.02 | 9.02E-05 | TRUE | 2.87E-35 |
| f__Sutterellaceae | BR | 0.02 | 3.24E-05 | TRUE | 4.57E-37 |
| f__Veillonellaceae | BR | 0.03 | 5.21E-05 | TRUE | 3.83E-49 |
| g__Alistipes | BCR | 0.03 | 1.43E-05 | TRUE | 7.74E-46 |
| g__Bacteroides | BCR | 0.04 | 6.40E-05 | TRUE | 4.67E-60 |
| g__Barnesiella | CR | 0.04 | 7.17E-05 | TRUE | 1.61E-61 |
| g__Barnesiella | BC | 0.04 | 4.88E-05 | TRUE | 1.66E-62 |
| g__Bifidobacterium | BR | 0.03 | 2.85E-05 | TRUE | 1.76E-52 |
| g__Butyrivibrio | BCR | 0.03 | 4.82E-05 | TRUE | 2.68E-45 |
| g__Clostridium | CC | 0.03 | 1.51E-05 | TRUE | 4.47E-42 |
| g__Coprococcus | BC | 0.03 | 4.92E-05 | TRUE | 1.98E-43 |
| g__Coprococcus | BCR | 0.03 | 5.79E-05 | TRUE | 3.96E-43 |
| g__Coprococcus | BR | 0.03 | 5.27E-05 | TRUE | 2.89E-43 |
| g__Desulfovibrio | CR | 0.01 | 1.89E-05 | TRUE | 9.17E-10 |
| g__Desulfovibrio | BCR | 0.01 | 5.42E-06 | TRUE | 2.94E-10 |
| g__Dorea | CR | 0.01 | 1.68E-05 | TRUE | 1.96E-23 |
| g__Faecalibacterium | BC | 0.02 | 9.48E-06 | TRUE | 1.98E-32 |
| g__Flavonifractor | CC | 0.01 | 3.59E-05 | TRUE | 8.75E-22 |
| g__Flavonifractor | CRC | 0.01 | 4.23E-05 | TRUE | 1.33E-21 |
| g__Gordonibacter | BCR | 0.01 | 9.30E-06 | TRUE | 1.16E-18 |
| g__Gordonibacter | BR | 0.01 | 3.14E-05 | TRUE | 8.46E-18 |
| g__Lactobacillus | BR | 0.03 | 7.91E-05 | TRUE | 5.45E-46 |
| g__Odoribacter | BR | 0.02 | 1.96E-05 | TRUE | 3.74E-40 |
| g__Parabacteroides | CRC | 0.03 | 6.43E-05 | TRUE | 3.91E-55 |
| g__Parabacteroides | CR | 0.03 | 8.12E-05 | TRUE | 1.55E-54 |
| g__Pseudoflavonifractor | CRC | 0.02 | 7.00E-05 | TRUE | 2.00E-38 |
| g__Pseudoflavonifractor | BC | 0.02 | 3.82E-05 | TRUE | 1.58E-39 |
| g__Pseudoflavonifractor | BCR | 0.02 | 5.20E-05 | TRUE | 5.01E-39 |
| g__Rothia | CC | 0.02 | 1.09E-05 | TRUE | 1.34E-28 |
| g__Streptococcus | CC | 0.02 | 1.36E-05 | TRUE | 1.67E-32 |
| g__Streptococcus | CRC | 0.02 | 4.40E-05 | TRUE | 3.35E-31 |
| g__Subdoligranulum | BCR | 0.04 | 4.05E-05 | TRUE | 2.86E-58 |
| g__Sutterella | CC | 0.01 | 5.00E-05 | TRUE | 5.30E-17 |
| g__Sutterella | CRC | 0.01 | 2.44E-05 | TRUE | 1.08E-17 |
| g__Sutterellaceae_unclassified | BR | 0.02 | 4.45E-05 | TRUE | 1.86E-25 |
| GLUCARDEG.PWY..D.glucarate.degradation.I | CC | 0.01 | 6.64E-06 | TRUE | 2.99E-14 |
| GLUCARDEG.PWY..D.glucarate.degradation.I | BC | 0.01 | 8.21E-06 | TRUE | 3.54E-14 |
| GLUCARDEG.PWY..D.glucarate.degradation.I | BCR | 0.01 | 5.83E-06 | TRUE | 2.60E-14 |
| GLYCOL.GLYOXDEG.PWY..superpathway.of.glycol.metabolism.and.degradation | CR | 0.03 | 7.42E-05 | TRUE | 6.72E-47 |
| HOMOSER.METSYN.PWY..L.methionine.biosynthesis.I | BR | 0.02 | 2.53E-05 | TRUE | 4.24E-36 |
| HSERMETANA.PWY..L.methionine.biosynthesis.III | BR | 0.04 | 8.34E-05 | TRUE | 1.66E-70 |
| o__Bacteroidales | BC | 0.04 | 6.74E-05 | TRUE | 2.49E-59 |
| o__Bacteroidales | BCR | 0.04 | 6.38E-05 | TRUE | 1.76E-59 |
| o__Burkholderiales | BC | 0.04 | 5.18E-05 | TRUE | 3.68E-62 |
| o__Erysipelotrichales | CRC | 0.02 | 2.85E-05 | TRUE | 1.19E-34 |
| o__Erysipelotrichales | CR | 0.02 | 1.56E-05 | TRUE | 2.72E-35 |
| o__Pasteurellales | CR | 0.01 | 6.71E-06 | TRUE | 5.27E-14 |
| o__Selenomonadales | BR | 0.02 | 2.03E-05 | TRUE | 1.56E-33 |
| p__Bacteroidetes | BC | 0.04 | 6.74E-05 | TRUE | 2.71E-59 |
| p__Bacteroidetes | BCR | 0.04 | 6.38E-05 | TRUE | 1.91E-59 |
| P122.PWY..heterolactic.fermentation | CRC | 0.02 | 4.01E-06 | TRUE | 8.45E-26 |
| PANTOSYN.PWY..pantothenate.and.coenzyme.A.biosynthesis.I | BC | 0.02 | 7.21E-05 | TRUE | 4.33E-33 |
| PANTOSYN.PWY..pantothenate.and.coenzyme.A.biosynthesis.I | BCR | 0.02 | 6.06E-05 | TRUE | 2.03E-33 |
| PENTOSE.P.PWY..pentose.phosphate.pathway | CR | 0.03 | 2.44E-05 | TRUE | 7.50E-55 |
| PWY.4984..urea.cycle | BC | 0.02 | 2.25E-05 | TRUE | 1.02E-33 |
| PWY.4984..urea.cycle | BCR | 0.02 | 3.22E-05 | TRUE | 2.68E-33 |
| PWY.5686..UMP.biosynthesis | BR | 0.02 | 3.74E-05 | TRUE | 7.76E-26 |
| PWY.5971..palmitate.biosynthesis.II..bacteria.and.plants. | CC | 0.03 | 9.58E-05 | TRUE | 1.45E-46 |
| PWY.6123..inosine.5..phosphate.biosynthesis.I | CRC | 0.02 | 1.87E-05 | TRUE | 4.03E-26 |
| PWY.6284..superpathway.of.unsaturated.fatty.acids.biosynthesis..E..coli. | BCR | 0.02 | 8.91E-06 | TRUE | 1.20E-40 |
| PWY.6471..peptidoglycan.biosynthesis.IV..Enterococcus.faecium. | BR | 0.01 | 2.57E-05 | TRUE | 2.88E-14 |
| PWY.6608..guanosine.nucleotides.degradation.III | CRC | 0.03 | 3.76E-05 | TRUE | 4.44E-45 |
| PWY.6608..guanosine.nucleotides.degradation.III | CR | 0.03 | 5.81E-05 | TRUE | 2.82E-44 |
| PWY.6690..cinnamate.and.3.hydroxycinnamate.degradation.to.2.oxopent.4.enoate | CR | 0.02 | 4.51E-05 | TRUE | 1.50E-33 |
| PWY.7219..adenosine.ribonucleotides.de.novo.biosynthesis | BC | 0.03 | 6.46E-05 | TRUE | 5.06E-53 |
| PWY.7371..1.4.dihydroxy.6.naphthoate.biosynthesis.II | BC | 0.02 | 5.61E-05 | TRUE | 4.79E-36 |
| PWY_GLYOXYLATE.BYPASS..glyoxylate.cycle | BCR | 0.03 | 4.33E-05 | TRUE | 7.24E-48 |
| PWY0.1297..superpathway.of.purine.deoxyribonucleosides.degradation | CC | 0.03 | 4.22E-05 | TRUE | 1.48E-45 |
| PWY0.1298..superpathway.of.pyrimidine.deoxyribonucleosides.degradation | CC | 0.04 | 8.40E-05 | TRUE | 8.09E-59 |
| PWY0.1479..tRNA.processing | CRC | 0.02 | 4.11E-05 | TRUE | 7.50E-39 |
| PWY0.1479..tRNA.processing | CR | 0.02 | 3.86E-05 | TRUE | 6.15E-39 |
| PWY4FS.7..phosphatidylglycerol.biosynthesis.I..plastidic. | BC | 0.04 | 5.05E-05 | TRUE | 4.10E-60 |
| s__Bacteroides_clarus | BR | 0.02 | 6.04E-05 | TRUE | 9.73E-39 |
| s__Bacteroides_eggerthii | CR | 0.02 | 2.63E-05 | TRUE | 5.51E-31 |
| s__Bacteroides_faecis | CC | 0.01 | 1.92E-06 | TRUE | 3.68E-19 |
| s__Bacteroides_finegoldii | BC | 0.04 | 6.73E-05 | TRUE | 3.03E-67 |
| s__Bacteroides_ovatus | CC | 0.04 | 6.42E-05 | TRUE | 1.61E-65 |
| s__Bacteroides_ovatus | BC | 0.04 | 8.01E-05 | TRUE | 5.70E-65 |
| s__Bacteroides_ovatus | BCR | 0.04 | 8.19E-05 | TRUE | 6.33E-65 |
| s__Bacteroides_ovatus | BR | 0.04 | 4.55E-05 | TRUE | 2.42E-66 |
| s__Bacteroides_xylanisolvens | CR | 0.04 | 3.77E-05 | TRUE | 7.95E-59 |
| s__Bifidobacterium_adolescentis | CR | 0.03 | 7.94E-05 | TRUE | 1.67E-50 |
| s__Bifidobacterium_adolescentis | BC | 0.03 | 6.91E-05 | TRUE | 6.90E-51 |
| s__Bifidobacterium_adolescentis | BCR | 0.03 | 6.84E-05 | TRUE | 6.33E-51 |
| s__Bifidobacterium_catenulatum | BCR | 0.02 | 3.65E-06 | TRUE | 1.91E-27 |
| s__Bifidobacterium_longum | BC | 0.03 | 5.40E-05 | TRUE | 3.17E-50 |
| s__Bilophila_wadsworthia | BCR | 0.02 | 6.47E-05 | TRUE | 1.27E-29 |
| s__Bilophila_wadsworthia | BR | 0.02 | 8.34E-05 | TRUE | 3.96E-29 |
| s__Coprococcus_catus | CRC | 0.01 | 3.60E-05 | TRUE | 4.82E-17 |
| s__Coprococcus_catus | CR | 0.01 | 2.55E-05 | TRUE | 2.41E-17 |
| s__Coprococcus_catus | BR | 0.01 | 2.21E-05 | TRUE | 1.85E-17 |
| s__Coprococcus_sp_ART55_1 | CR | 0.01 | 4.51E-05 | TRUE | 9.73E-18 |
| s__Desulfovibrio_piger | CC | 0.02 | 1.04E-05 | TRUE | 6.70E-27 |
| s__Dorea_longicatena | CR | 0.02 | 2.51E-05 | TRUE | 3.76E-40 |
| s__Eubacterium_hallii | BR | 0.03 | 1.03E-04 | TRUE | 6.12E-52 |
| s__Eubacterium_rectale | BR | 0.02 | 9.98E-06 | TRUE | 3.96E-36 |
| s__Eubacterium_siraeum | CRC | 0.04 | 1.19E-04 | TRUE | 3.91E-57 |
| s__Eubacterium_siraeum | CR | 0.04 | 1.04E-04 | TRUE | 1.49E-57 |
| s__Eubacterium_ventriosum | CC | 0.02 | 3.29E-05 | TRUE | 2.20E-27 |
| s__Faecalibacterium_prausnitzii | BC | 0.02 | 9.95E-06 | TRUE | 6.55E-37 |
| s__Flavonifractor_plautii | CC | 0.02 | 4.12E-05 | TRUE | 9.32E-26 |
| s__Gordonibacter_pamelaeae | BCR | 0.01 | 9.30E-06 | TRUE | 1.22E-18 |
| s__Gordonibacter_pamelaeae | BR | 0.01 | 3.14E-05 | TRUE | 8.95E-18 |
| s__Holdemania_filiformis | BCR | 0.01 | 1.72E-05 | TRUE | 4.98E-14 |
| s__Holdemania_filiformis | BR | 0.01 | 1.17E-05 | TRUE | 3.14E-14 |
| s__Holdemania_unclassified | CC | 0.03 | 5.39E-05 | TRUE | 3.76E-42 |
| s__Lachnospiraceae_bacterium_1_1_57FAA | CRC | 0.00 | 2.00E-05 | TRUE | 7.00E-05 |
| s__Lachnospiraceae_bacterium_1_1_57FAA | CR | 0.00 | 2.37E-05 | TRUE | 8.12E-05 |
| s__Lachnospiraceae_bacterium_3_1_46FAA | BR | 0.01 | 1.22E-05 | TRUE | 4.73E-18 |
| s__Lachnospiraceae_bacterium_5_1_63FAA | BC | 0.01 | 4.26E-05 | TRUE | 6.71E-21 |
| s__Lactobacillus_delbrueckii | CC | 0.04 | 1.32E-04 | TRUE | 2.12E-57 |
| s__Lactobacillus_delbrueckii | CRC | 0.04 | 1.30E-04 | TRUE | 1.84E-57 |
| s__Oscillibacter_unclassified | CR | 0.02 | 1.82E-05 | TRUE | 3.08E-32 |
| s__Parabacteroides_distasonis | CRC | 0.02 | 3.56E-05 | TRUE | 1.43E-36 |
| s__Parabacteroides_distasonis | CR | 0.02 | 4.01E-05 | TRUE | 2.19E-36 |
| s__Parabacteroides_goldsteinii | CC | 0.02 | 5.50E-05 | TRUE | 2.78E-30 |
| s__Parabacteroides_goldsteinii | CRC | 0.02 | 5.18E-05 | TRUE | 2.20E-30 |
| s__Paraprevotella_xylaniphila | CC | 0.03 | 2.09E-05 | TRUE | 5.13E-49 |
| s__Prevotella_copri | CC | 0.03 | 6.40E-05 | TRUE | 1.85E-50 |
| s__Prevotella_copri | BR | 0.03 | 9.88E-05 | TRUE | 2.24E-49 |
| s__Pseudoflavonifractor_capillosus | CRC | 0.03 | 6.89E-05 | TRUE | 1.05E-47 |
| s__Pseudoflavonifractor_capillosus | BC | 0.03 | 4.98E-05 | TRUE | 2.02E-48 |
| s__Pseudoflavonifractor_capillosus | BCR | 0.03 | 5.50E-05 | TRUE | 3.11E-48 |
| s__Roseburia_hominis | CR | 0.02 | 2.61E-05 | TRUE | 3.24E-32 |
| s__Roseburia_intestinalis | CC | 0.02 | 9.90E-05 | TRUE | 9.94E-38 |
| s__Roseburia_inulinivorans | CC | 0.01 | 6.39E-06 | TRUE | 5.53E-20 |
| s__Roseburia_inulinivorans | CRC | 0.01 | 3.08E-06 | TRUE | 2.95E-20 |
| s__Rothia_mucilaginosa | CC | 0.01 | 5.26E-06 | TRUE | 3.32E-19 |
| s__Streptococcus_parasanguinis | CC | 0.01 | 6.55E-06 | TRUE | 1.10E-14 |
| s__Streptococcus_parasanguinis | BC | 0.01 | 5.79E-06 | TRUE | 9.68E-15 |
| s__Streptococcus_parasanguinis | BCR | 0.01 | 4.18E-06 | TRUE | 7.50E-15 |
| s__Streptococcus_thermophilus | CC | 0.01 | 1.56E-05 | TRUE | 1.33E-18 |
| s__Streptococcus_thermophilus | CRC | 0.01 | 3.77E-05 | TRUE | 7.00E-18 |
| s__Streptococcus_thermophilus | CR | 0.01 | 4.28E-05 | TRUE | 9.60E-18 |
| s__Subdoligranulum_unclassified | BCR | 0.03 | 3.98E-05 | TRUE | 1.88E-53 |
| s__Sutterella_wadsworthensis | CC | 0.01 | 5.00E-05 | TRUE | 2.88E-17 |
| s__Sutterella_wadsworthensis | CRC | 0.01 | 2.44E-05 | TRUE | 5.77E-18 |
| s__Veillonella_unclassified | CR | 0.03 | 6.33E-05 | TRUE | 2.03E-42 |
| SO4ASSIM.PWY..sulfate.reduction.I..assimilatory. | CC | 0.01 | 2.93E-06 | TRUE | 8.24E-20 |
| SO4ASSIM.PWY..sulfate.reduction.I..assimilatory. | CRC | 0.01 | 6.60E-06 | TRUE | 1.62E-19 |
| X1CMET2.PWY..N10.formyl.tetrahydrofolate.biosynthesis | CC | 0.03 | 2.16E-05 | TRUE | 6.95E-43 |

*Note:* CRC: colorectal cancer; CC: malignant tumors of colon; CR: malignant tumors of rectum; BCR: benign tumors of colorectum; BC: benign tumors of colon; BR: benign tumors of rectum.

**Table S7. Colocalization analysis where loci provide evidence of a shared causal variant.**

| **Exposure** | **Outcome** | **nsnps** | **PP.H0.abf** | **PP.H1.abf** | **PP.H2.abf** | **PP.H3.abf** | **PP.H4.abf** |
| --- | --- | --- | --- | --- | --- | --- | --- |
| ARGORNPROST.PWY..arginine..ornithine.and.proline.interconversion | CC | 7 | 0.14 | 6.83E-06 | 0.85 | 3.58E-05 | 0.01 |
| o__Bacteroidales | BC | 13 | 0.03 | 5.85E-06 | 0.95 | 1.55E-04 | 0.01 |
| o__Bacteroidales | BCR | 13 | 0.03 | 5.68E-06 | 0.95 | 1.46E-04 | 0.02 |
| o__Burkholderiales | BC | 14 | 0.09 | 1.15E-05 | 0.90 | 1.09E-04 | 0.01 |
| o__Erysipelotrichales | CR | 8 | 0.21 | 1.19E-05 | 0.78 | 3.68E-05 | 0.01 |
| o__Erysipelotrichales | CRC | 8 | 0.21 | 1.47E-05 | 0.78 | 4.48E-05 | 0.01 |
| o__Pasteurellales | CR | 3 | 0.46 | 8.49E-06 | 0.53 | 6.89E-06 | 0.00 |
| o__Selenomonadales | BR | 7 | 0.03 | 1.29E-06 | 0.97 | 3.99E-05 | 0.00 |
| COA.PWY..coenzyme.A.biosynthesis.I | CC | 9 | 0.19 | 9.27E-06 | 0.81 | 3.59E-05 | 0.00 |
| COLANSYN.PWY..colanic.acid.building.blocks.biosynthesis | BC | 2 | 0.08 | 1.65E-06 | 0.91 | 8.73E-06 | 0.01 |
| g__Alistipes | BCR | 10 | 0.01 | 7.52E-07 | 0.98 | 4.78E-05 | 0.01 |
| g__Barnesiella | BC | 14 | 0.06 | 6.24E-06 | 0.94 | 9.65E-05 | 0.01 |
| g__Barnesiella | CR | 14 | 0.06 | 1.37E-05 | 0.94 | 2.16E-04 | 0.01 |
| g__Bifidobacterium | BR | 10 | 0.00 | 6.88E-13 | 1.00 | 5.93E-05 | 0.00 |
| g__Butyrivibrio | BCR | 10 | 0.08 | 8.10E-06 | 0.91 | 8.24E-05 | 0.01 |
| g__Desulfovibrio | BCR | 2 | 0.38 | 4.67E-06 | 0.61 | 3.64E-06 | 0.00 |
| g__Desulfovibrio | CR | 2 | 0.38 | 1.01E-05 | 0.61 | 6.96E-06 | 0.01 |
| g__Dorea | CR | 5 | 0.06 | 1.99E-06 | 0.94 | 2.77E-05 | 0.00 |
| g__Flavonifractor | CC | 5 | 0.25 | 1.18E-05 | 0.74 | 2.88E-05 | 0.01 |
| g__Flavonifractor | CRC | 5 | 0.25 | 2.37E-05 | 0.74 | 5.97E-05 | 0.01 |
| g__Gordonibacter | BCR | 4 | 0.34 | 7.35E-06 | 0.66 | 1.07E-05 | 0.00 |
| g__Gordonibacter | BR | 4 | 0.34 | 2.19E-05 | 0.65 | 3.17E-05 | 0.01 |
| g__Lactobacillus | BR | 11 | 0.16 | 3.27E-05 | 0.82 | 1.52E-04 | 0.02 |
| g__Odoribacter | BR | 9 | 0.15 | 7.25E-06 | 0.85 | 3.85E-05 | 0.00 |
| g__Parabacteroides | CR | 13 | 0.17 | 2.91E-05 | 0.82 | 1.34E-04 | 0.01 |
| g__Parabacteroides | CRC | 13 | 0.17 | 1.95E-05 | 0.83 | 9.04E-05 | 0.01 |
| g__Rothia | CC | 6 | 0.16 | 5.64E-06 | 0.83 | 2.49E-05 | 0.00 |
| g__Subdoligranulum | BCR | 13 | 0.10 | 1.12E-05 | 0.90 | 9.63E-05 | 0.01 |
| GLUCARDEG.PWY..D.glucarate.degradation.I | BC | 3 | 0.40 | 8.75E-06 | 0.60 | 8.57E-06 | 0.00 |
| GLUCARDEG.PWY..D.glucarate.degradation.I | BCR | 3 | 0.40 | 7.38E-06 | 0.60 | 7.26E-06 | 0.00 |
| GLUCARDEG.PWY..D.glucarate.degradation.I | CC | 3 | 0.40 | 8.04E-06 | 0.60 | 7.68E-06 | 0.00 |
| GLYCOL.GLYOXDEG.PWY..superpathway.of.glycol.metabolism.and.degradation | CR | 11 | 0.13 | 1.74E-05 | 0.86 | 1.00E-04 | 0.01 |
| HSERMETANA.PWY..L.methionine.biosynthesis.III | BR | 16 | 0.05 | 9.37E-06 | 0.93 | 1.61E-04 | 0.02 |
| s__Bifidobacterium_catenulatum | BCR | 6 | 0.29 | 9.27E-06 | 0.71 | 1.93E-05 | 0.00 |
| p__Bacteroidetes | BC | 13 | 0.03 | 5.83E-06 | 0.95 | 1.55E-04 | 0.01 |
| p__Bacteroidetes | BCR | 13 | 0.03 | 5.67E-06 | 0.95 | 1.46E-04 | 0.02 |
| g__Bacteroides | BCR | 14 | 0.13 | 2.18E-05 | 0.85 | 1.25E-04 | 0.01 |
| s__Bacteroides_xylanisolvens | CR | 13 | 0.04 | 3.87E-06 | 0.94 | 7.30E-05 | 0.02 |
| g__Streptococcus | CC | 7 | 0.15 | 5.42E-06 | 0.85 | 2.60E-05 | 0.00 |
| g__Streptococcus | CRC | 7 | 0.15 | 1.24E-05 | 0.84 | 6.25E-05 | 0.01 |
| g__Clostridium | CC | 9 | 0.10 | 4.55E-06 | 0.90 | 3.79E-05 | 0.00 |
| g__Pseudoflavonifractor | BC | 9 | 0.19 | 1.32E-05 | 0.80 | 4.98E-05 | 0.01 |
| g__Pseudoflavonifractor | BCR | 9 | 0.19 | 2.05E-05 | 0.80 | 7.81E-05 | 0.01 |
| g__Pseudoflavonifractor | CRC | 9 | 0.19 | 4.73E-05 | 0.79 | 1.82E-04 | 0.02 |
| g__Coprococcus | BC | 10 | 0.15 | 1.94E-05 | 0.84 | 1.01E-04 | 0.01 |
| g__Coprococcus | BCR | 10 | 0.15 | 1.59E-05 | 0.84 | 8.16E-05 | 0.01 |
| g__Coprococcus | BR | 10 | 0.15 | 1.22E-05 | 0.84 | 6.17E-05 | 0.01 |
| s__Lachnospiraceae_bacterium_3_1_46FAA | BR | 4 | 0.39 | 9.17E-06 | 0.61 | 1.08E-05 | 0.00 |
| s__Roseburia_hominis | CR | 7 | 0.17 | 7.18E-06 | 0.82 | 2.94E-05 | 0.00 |
| g__Faecalibacterium | BC | 7 | 0.17 | 6.31E-06 | 0.82 | 2.53E-05 | 0.01 |
| s__Subdoligranulum_unclassified | BCR | 12 | 0.11 | 1.17E-05 | 0.89 | 8.95E-05 | 0.01 |
| g__Sutterella | CC | 4 | 0.38 | 3.39E-05 | 0.60 | 3.80E-05 | 0.02 |
| g__Sutterella | CRC | 4 | 0.39 | 1.46E-05 | 0.61 | 1.74E-05 | 0.01 |
| g__Sutterellaceae_unclassified | BR | 6 | 0.30 | 2.22E-05 | 0.70 | 4.41E-05 | 0.01 |
| f__Micrococcaceae | CC | 6 | 0.18 | 6.34E-06 | 0.82 | 2.43E-05 | 0.00 |
| f__Bacteroidaceae | BCR | 13 | 0.14 | 2.17E-05 | 0.85 | 1.20E-04 | 0.01 |
| f__Prevotellaceae | BR | 8 | 0.09 | 4.53E-06 | 0.90 | 4.03E-05 | 0.00 |
| f__Rikenellaceae | BCR | 10 | 0.01 | 7.41E-07 | 0.98 | 4.78E-05 | 0.01 |
| f__Sutterellaceae | BR | 8 | 0.10 | 5.95E-06 | 0.89 | 4.69E-05 | 0.01 |
| f__Sutterellaceae | CC | 8 | 0.10 | 9.58E-06 | 0.89 | 7.61E-05 | 0.01 |
| f__Sutterellaceae | CRC | 8 | 0.10 | 4.87E-05 | 0.87 | 3.95E-04 | 0.03 |
| f__Clostridiaceae | CC | 8 | 0.11 | 4.59E-06 | 0.89 | 3.33E-05 | 0.00 |
| f__Lachnospiraceae | CC | 11 | 0.10 | 1.64E-05 | 0.89 | 1.33E-04 | 0.01 |
| f__Oscillospiraceae | CR | 7 | 0.20 | 8.18E-06 | 0.79 | 2.74E-05 | 0.00 |
| f__Erysipelotrichaceae | CR | 8 | 0.21 | 1.19E-05 | 0.78 | 3.68E-05 | 0.01 |
| f__Erysipelotrichaceae | CRC | 8 | 0.21 | 1.47E-05 | 0.78 | 4.48E-05 | 0.01 |
| f__Lactobacillaceae | BR | 12 | 0.15 | 3.45E-05 | 0.83 | 1.66E-04 | 0.02 |
| f__Streptococcaceae | CC | 13 | 0.10 | 6.97E-06 | 0.90 | 6.05E-05 | 0.01 |
| f__Pasteurellaceae | CR | 3 | 0.46 | 8.50E-06 | 0.53 | 6.89E-06 | 0.00 |
| f__Acidaminococcaceae | BCR | 7 | 0.25 | 1.26E-05 | 0.74 | 3.09E-05 | 0.01 |
| f__Acidaminococcaceae | BR | 7 | 0.25 | 2.68E-05 | 0.74 | 6.92E-05 | 0.01 |
| f__Veillonellaceae | BR | 11 | 0.07 | 6.82E-06 | 0.93 | 8.61E-05 | 0.01 |
| c__Bacteroidia | BC | 13 | 0.03 | 5.84E-06 | 0.95 | 1.55E-04 | 0.01 |
| c__Bacteroidia | BCR | 13 | 0.03 | 5.68E-06 | 0.95 | 1.46E-04 | 0.02 |
| c__Bacilli | CC | 13 | 0.09 | 1.01E-05 | 0.90 | 9.60E-05 | 0.00 |
| c__Erysipelotrichia | CR | 8 | 0.21 | 1.19E-05 | 0.78 | 3.68E-05 | 0.01 |
| c__Erysipelotrichia | CRC | 8 | 0.21 | 1.47E-05 | 0.78 | 4.48E-05 | 0.01 |
| c__Negativicutes | BR | 7 | 0.03 | 1.29E-06 | 0.97 | 3.99E-05 | 0.00 |
| c__Betaproteobacteria | CC | 13 | 0.09 | 1.09E-05 | 0.90 | 1.03E-04 | 0.01 |
| c__Gammaproteobacteria | BC | 4 | 0.07 | 1.87E-06 | 0.92 | 1.74E-05 | 0.01 |
| c__Gammaproteobacteria | CC | 4 | 0.07 | 4.12E-06 | 0.91 | 3.32E-05 | 0.02 |
| c__Gammaproteobacteria | CRC | 4 | 0.07 | 3.86E-06 | 0.92 | 4.08E-05 | 0.01 |
| P122.PWY..heterolactic.fermentation | CRC | 5 | 0.07 | 2.01E-06 | 0.93 | 2.26E-05 | 0.01 |
| PANTOSYN.PWY..pantothenate.and.coenzyme.A.biosynthesis.I | BC | 8 | 0.26 | 4.18E-05 | 0.73 | 1.01E-04 | 0.02 |
| PANTOSYN.PWY..pantothenate.and.coenzyme.A.biosynthesis.I | BCR | 8 | 0.26 | 3.44E-05 | 0.73 | 8.48E-05 | 0.01 |
| PENTOSE.P.PWY..pentose.phosphate.pathway | CR | 12 | 0.10 | 6.85E-06 | 0.89 | 5.51E-05 | 0.01 |
| PWY.4984..urea.cycle | BC | 8 | 0.34 | 1.43E-05 | 0.66 | 2.42E-05 | 0.00 |
| PWY.4984..urea.cycle | BCR | 8 | 0.34 | 1.84E-05 | 0.66 | 3.15E-05 | 0.00 |
| PWY.5686..UMP.biosynthesis | BR | 6 | 0.29 | 1.70E-05 | 0.70 | 3.40E-05 | 0.01 |
| PWY.5971..palmitate.biosynthesis.II..bacteria.and.plants. | CC | 11 | 0.13 | 5.66E-05 | 0.83 | 3.16E-04 | 0.03 |
| PWY.6123..inosine.5..phosphate.biosynthesis.I | CRC | 6 | 0.30 | 1.27E-05 | 0.69 | 2.48E-05 | 0.00 |
| PWY.6284..superpathway.of.unsaturated.fatty.acids.biosynthesis..E..coli. | BCR | 9 | 0.20 | 8.60E-06 | 0.80 | 3.08E-05 | 0.00 |
| PWY.6471..peptidoglycan.biosynthesis.IV..Enterococcus.faecium | BR | 3 | 0.23 | 8.43E-06 | 0.76 | 1.55E-05 | 0.01 |
| PWY.6608..guanosine.nucleotides.degradation.III | CR | 10 | 0.05 | 6.62E-06 | 0.94 | 1.12E-04 | 0.01 |
| PWY.6608..guanosine.nucleotides.degradation.III | CRC | 10 | 0.05 | 5.59E-06 | 0.94 | 9.12E-05 | 0.01 |
| PWY.6628..superpathway.of.L.phenylalanine.biosynthesis | BR | 12 | 0.04 | 5.21E-06 | 0.94 | 9.92E-05 | 0.01 |
| PWY.6629..superpathway.of.L.tryptophan.biosynthesis | CRC | 7 | 0.09 | 4.83E-06 | 0.90 | 3.57E-05 | 0.01 |
| PWY.6690..cinnamate.and.3.hydroxycinnamate.degradation.to.2.oxopent.4.enoate | CR | 8 | 0.26 | 2.29E-05 | 0.74 | 5.80E-05 | 0.01 |
| PWY.6731..starch.degradation.III | BC | 13 | 0.09 | 2.04E-05 | 0.87 | 1.51E-04 | 0.04 |
| PWY.6823..molybdenum.cofactor.biosynthesis | BCR | 11 | 0.08 | 7.47E-06 | 0.91 | 7.46E-05 | 0.01 |
| PWY.7219..adenosine.ribonucleotides.de.novo.biosynthesis | BC | 12 | 0.08 | 8.40E-06 | 0.91 | 9.07E-05 | 0.01 |
| PWY.7371..1.4.dihydroxy.6.naphthoate.biosynthesis.II | BC | 8 | 0.04 | 1.88E-05 | 0.72 | 9.21E-05 | 0.24 |
| PWY_GLYOXYLATE.BYPASS..glyoxylate.cycle | BCR | 11 | 0.16 | 1.54E-05 | 0.83 | 6.81E-05 | 0.01 |
| PWY0.1297..superpathway.of.purine.deoxyribonucleosides.degradation | CC | 10 | 0.08 | 5.50E-06 | 0.91 | 5.64E-05 | 0.01 |
| PWY0.1298..superpathway.of.pyrimidine.deoxyribonucleosides.degradation | CC | 14 | 0.16 | 3.48E-05 | 0.83 | 1.73E-04 | 0.01 |
| PWY0.1479..tRNA.processing | CR | 9 | 0.18 | 1.43E-05 | 0.81 | 5.68E-05 | 0.01 |
| PWY0.1479..tRNA.processing | CRC | 9 | 0.18 | 1.25E-05 | 0.81 | 5.05E-05 | 0.01 |
| PWY4FS.7..phosphatidylglycerol.biosynthesis.I..plastidic. | BC | 14 | 0.13 | 1.52E-05 | 0.87 | 9.65E-05 | 0.01 |
| s__Bacteroides_clarus | BR | 9 | 0.14 | 1.33E-05 | 0.85 | 7.39E-05 | 0.01 |
| s__Bacteroides_eggerthii | CR | 7 | 0.21 | 1.18E-05 | 0.78 | 3.45E-05 | 0.01 |
| s__Bacteroides_faecis | CC | 4 | 0.34 | 7.24E-06 | 0.65 | 1.02E-05 | 0.00 |
| s__Bacteroides_finegoldii | BC | 15 | 0.06 | 8.37E-06 | 0.94 | 1.27E-04 | 0.01 |
| s__Bacteroides_ovatus | BC | 14 | 0.04 | 9.75E-06 | 0.94 | 1.89E-04 | 0.01 |
| s__Bacteroides_ovatus | BCR | 14 | 0.05 | 7.14E-06 | 0.95 | 1.41E-04 | 0.01 |
| s__Bacteroides_ovatus | BR | 14 | 0.05 | 3.97E-06 | 0.95 | 7.66E-05 | 0.01 |
| s__Bacteroides_ovatus | CC | 14 | 0.04 | 1.53E-05 | 0.91 | 2.79E-04 | 0.04 |
| s__Bifidobacterium_adolescentis | BC | 10 | 0.00 | 8.41E-12 | 0.99 | 9.27E-05 | 0.01 |
| s__Bifidobacterium_adolescentis | BCR | 10 | 0.00 | 7.75E-12 | 1.00 | 8.78E-05 | 0.00 |
| s__Bifidobacterium_adolescentis | CR | 10 | 0.00 | 1.37E-11 | 0.99 | 1.57E-04 | 0.01 |
| s__Bifidobacterium_longum | BC | 11 | 0.02 | 1.71E-06 | 0.97 | 7.76E-05 | 0.01 |
| s__Bilophila_wadsworthia | BCR | 7 | 0.13 | 6.38E-05 | 0.68 | 1.41E-04 | 0.19 |
| s__Bilophila_wadsworthia | BR | 7 | 0.14 | 5.45E-05 | 0.71 | 1.33E-04 | 0.15 |
| s__Coprococcus_catus | BR | 4 | 0.41 | 1.91E-05 | 0.58 | 1.93E-05 | 0.01 |
| s__Coprococcus_catus | CR | 4 | 0.41 | 1.61E-05 | 0.58 | 1.66E-05 | 0.01 |
| s__Coprococcus_catus | CRC | 4 | 0.41 | 2.45E-05 | 0.58 | 2.45E-05 | 0.01 |
| s__Coprococcus_sp_ART55_1 | CR | 4 | 0.27 | 7.32E-05 | 0.67 | 1.26E-04 | 0.05 |
| s__Desulfovibrio_piger | CC | 6 | 0.27 | 9.01E-06 | 0.73 | 2.00E-05 | 0.00 |
| s__Dorea_longicatena | CR | 9 | 0.14 | 7.31E-06 | 0.86 | 4.07E-05 | 0.00 |
| s__Eubacterium_hallii | BR | 12 | 0.08 | 2.11E-05 | 0.91 | 2.27E-04 | 0.01 |
| s__Eubacterium_rectale | BR | 8 | 0.21 | 7.49E-06 | 0.79 | 2.45E-05 | 0.00 |
| s__Eubacterium_siraeum | CR | 14 | 0.15 | 9.17E-05 | 0.82 | 4.79E-04 | 0.03 |
| s__Eubacterium_siraeum | CRC | 14 | 0.15 | 6.47E-05 | 0.83 | 3.37E-04 | 0.02 |
| s__Eubacterium_ventriosum | CC | 6 | 0.07 | 5.24E-06 | 0.92 | 5.75E-05 | 0.01 |
| s__Faecalibacterium_prausnitzii | BC | 8 | 0.14 | 5.75E-06 | 0.85 | 2.91E-05 | 0.01 |
| s__Flavonifractor_plautii | CC | 6 | 0.23 | 1.25E-05 | 0.76 | 3.51E-05 | 0.01 |
| s__Gordonibacter_pamelaeae | BCR | 4 | 0.34 | 7.43E-06 | 0.65 | 1.06E-05 | 0.00 |
| s__Gordonibacter_pamelaeae | BR | 4 | 0.34 | 2.21E-05 | 0.65 | 3.06E-05 | 0.01 |
| s__Holdemania_filiformis | BCR | 3 | 0.27 | 1.03E-05 | 0.72 | 1.44E-05 | 0.01 |
| s__Holdemania_filiformis | BR | 3 | 0.27 | 7.29E-06 | 0.72 | 1.33E-05 | 0.01 |
| s__Holdemania_unclassified | CC | 10 | 0.19 | 2.10E-05 | 0.80 | 7.95E-05 | 0.01 |
| s__Lachnospiraceae_bacterium_1_1_57FAA | CR | 1 | 0.79 | 5.92E-05 | 0.20 | 0.00E+00 | 0.01 |
| s__Lachnospiraceae_bacterium_1_1_57FAA | CRC | 1 | 0.79 | 4.01E-05 | 0.20 | 0.00E+00 | 0.01 |
| s__Lachnospiraceae_bacterium_5_1_63FAA | BC | 5 | 0.39 | 7.55E-05 | 0.59 | 9.68E-05 | 0.02 |
| s__Lactobacillus_delbrueckii | CC | 14 | 0.14 | 4.46E-05 | 0.84 | 2.41E-04 | 0.02 |
| s__Lactobacillus_delbrueckii | CRC | 14 | 0.15 | 3.47E-05 | 0.84 | 1.89E-04 | 0.01 |
| s__Oscillibacter_unclassified | CR | 7 | 0.19 | 7.80E-06 | 0.80 | 2.79E-05 | 0.00 |
| s__Parabacteroides_distasonis | CR | 8 | 0.04 | 4.54E-06 | 0.95 | 1.00E-04 | 0.01 |
| s__Parabacteroides_distasonis | CRC | 8 | 0.04 | 2.47E-06 | 0.95 | 5.06E-05 | 0.01 |
| s__Parabacteroides_goldsteinii | CC | 7 | 0.13 | 1.09E-05 | 0.86 | 5.92E-05 | 0.01 |
| s__Parabacteroides_goldsteinii | CRC | 7 | 0.13 | 1.06E-05 | 0.84 | 4.43E-05 | 0.02 |
| s__Paraprevotella_xylaniphila | CC | 11 | 0.15 | 9.34E-06 | 0.85 | 4.82E-05 | 0.00 |
| s__Prevotella_copri | BR | 12 | 0.17 | 9.78E-05 | 0.76 | 3.78E-04 | 0.07 |
| s__Prevotella_copri | CC | 12 | 0.18 | 2.04E-05 | 0.81 | 8.37E-05 | 0.01 |
| s__Pseudoflavonifractor_capillosus | BC | 11 | 0.14 | 1.26E-05 | 0.85 | 7.05E-05 | 0.01 |
| s__Pseudoflavonifractor_capillosus | BCR | 11 | 0.14 | 1.70E-05 | 0.85 | 9.64E-05 | 0.01 |
| s__Pseudoflavonifractor_capillosus | CRC | 11 | 0.14 | 3.65E-05 | 0.85 | 2.09E-04 | 0.01 |
| s__Roseburia_intestinalis | CC | 9 | 0.16 | 5.88E-05 | 0.81 | 2.71E-04 | 0.03 |
| s__Roseburia_inulinivorans | CC | 4 | 0.14 | 3.14E-06 | 0.86 | 1.43E-05 | 0.00 |
| s__Roseburia_inulinivorans | CRC | 4 | 0.14 | 2.87E-06 | 0.85 | 1.26E-05 | 0.00 |
| s__Rothia_mucilaginosa | CC | 4 | 0.23 | 4.58E-06 | 0.77 | 1.14E-05 | 0.00 |
| s__Streptococcus_parasanguinis | BC | 3 | 0.34 | 6.07E-06 | 0.65 | 7.73E-06 | 0.00 |
| s__Streptococcus_parasanguinis | BCR | 3 | 0.34 | 5.78E-06 | 0.65 | 7.19E-06 | 0.00 |
| s__Streptococcus_parasanguinis | CC | 3 | 0.34 | 6.53E-06 | 0.65 | 8.39E-06 | 0.00 |
| s__Streptococcus_thermophilus | CC | 4 | 0.22 | 5.44E-06 | 0.77 | 1.50E-05 | 0.00 |
| s__Streptococcus_thermophilus | CR | 4 | 0.22 | 1.85E-05 | 0.77 | 5.14E-05 | 0.01 |
| s__Streptococcus_thermophilus | CRC | 4 | 0.22 | 1.25E-05 | 0.77 | 3.63E-05 | 0.01 |
| s__Sutterella_wadsworthensis | CC | 4 | 0.36 | 3.15E-05 | 0.63 | 4.11E-05 | 0.01 |
| s__Sutterella_wadsworthensis | CRC | 4 | 0.36 | 1.36E-05 | 0.63 | 1.82E-05 | 0.01 |
| s__Veillonella_unclassified | CR | 10 | 0.19 | 2.21E-05 | 0.80 | 8.25E-05 | 0.01 |
| SO4ASSIM.PWY..sulfate.reduction.I..assimilatory. | CC | 4 | 0.17 | 2.46E-06 | 0.83 | 9.07E-06 | 0.00 |
| SO4ASSIM.PWY..sulfate.reduction.I..assimilatory. | CRC | 4 | 0.17 | 2.72E-06 | 0.83 | 9.99E-06 | 0.00 |
| X1CMET2.PWY..N10.formyl.tetrahydrofolate.biosynthesis | CC | 9 | 0.07 | 3.55E-06 | 0.92 | 3.91E-05 | 0.01 |

*Note:* CRC: colorectal cancer; CC: malignant tumors of colon; CR: malignant tumors of rectum; BCR: benign tumors of colorectum; BC: benign tumors of colon; BR: benign tumors of rectum;nsnp is the number of SNPs being used as IVs; PP.H4.abf > 0·8 were marked in bold.

**Table S8. MR-BMA ranking of microbial biomarkers for malignant tumors based on MIP and MACE in potential biomarkers ranking analysis.**

| **Colorectal cancer** | | | | **Malignant tumors of the colon** | | | | **Malignant tumors of the rectal** | | | |
| --- | --- | --- | --- | --- | --- | --- | --- | --- | --- | --- | --- |
| **Exposure** | **MIP** | **MACE** | **Pval** | **Exposure** | **MIP** | **MACE** | **Pval** | **Exposure** | **MIP** | **MACE** | **Pval** |
| g__Pseudoflavonifractor | 0.906 | 0.257 | 0.020 | c__Gammaproteobacteria | 0.204 | 0.049 | 0.089 | s__Coprococcus_sp_ART55_1 | 0.454 | -0.094 | 0.010 |
| s__Streptococcus_thermophilus | 0.562 | -0.106 | 0.020 | s__Eubacterium_ventriosum | 0.169 | -0.025 | 0.030 | g__Desulfovibrio | 0.36 | -0.112 | 0.020 |
| s__Coprococcus_catus | 0.433 | -0.114 | 0.010 | s__Holdemania_unclassified | 0.148 | -0.02 | 0.059 | f__Erysipelotrichaceae | 0.165 | -0.04 | 0.109 |
| s__Parabacteroides_distasonis | 0.076 | 0.011 | 0.347 | s__Sutterella_wadsworthensis | 0.136 | -0.024 | 0.158 | s__Coprococcus_catus | 0.072 | -0.013 | 0.347 |
| f__Erysipelotrichaceae | 0.065 | -0.01 | 0.535 | s__Prevotella_copri | 0.088 | 0.012 | 0.188 | g__Barnesiella | 0.06 | 0.009 | 0.446 |
| s__Sutterella_wadsworthensis | 0.056 | -0.009 | 0.802 | s__Roseburia_inulinivorans | 0.082 | 0.012 | 0.198 | g__Dorea | 0.058 | -0.009 | 0.564 |
| g__Streptococcus | 0.055 | -0.003 | 0.455 | c__Betaproteobacteria | 0.078 | -0.01 | 0.366 | s__Streptococcus_thermophilus | 0.054 | -0.007 | 0.396 |
| s__Roseburia_inulinivorans | 0.029 | 0.002 | 1.000 | s__Paraprevotella_xylaniphila | 0.071 | -0.007 | 0.188 | s__Lachnospiraceae_bacterium_1_1_57FAA | 0.053 | -0.007 | 0.416 |
| g__Flavonifractor | 0.028 | 0.002 | 0.960 | f__Sutterellaceae | 0.071 | -0.008 | 0.495 | f__Pasteurellaceae | 0.051 | 0.006 | 0.465 |
| c__Gammaproteobacteria | 0.026 | -0.001 | 1.000 | s_Parabacteroides_goldsteinii | 0.064 | 0.005 | 0.079 | s__Roseburia_hominis | 0.048 | 0.005 | 0.871 |

**Table S9. MR-BMA ranking of microbial biomarkers for benign tumors based on MIP and MACE in potential biomarkers ranking analysis.**

| **benign tumors of the colorectum** | | | | **benign tumors of the colon** | | | | **benign tumors of the rectal** | | | |
| --- | --- | --- | --- | --- | --- | --- | --- | --- | --- | --- | --- |
| **Exposure** | **MIP** | **MACE** | **Pval** | **Exposure** | **MIP** | **MACE** | **Pval** | **Exposure** | **MIP** | **MACE** | **Pval** |
| o__Bacteroidales | 0.433 | 0.063 | 0.020 | s__Bacteroides_ovatus | 0.809 | 0.122 | 0.010 | f__Acidaminococcaceae | 0.818 | -0.169 | 0.010 |
| s__Bifidobacterium_adolescentis | 0.342 | -0.046 | 0.040 | s__Bifidobacterium_adolescentis | 0.081 | -0.008 | 0.347 | s__Eubacterium_rectale | 0.348 | -0.086 | 0.030 |
| s__Bacteroides_ovatus | 0.255 | 0.031 | 0.030 | s__Lachnospiraceae_bacterium_5_1_63FAA | 0.078 | 0.005 | 0.188 | g__Sutterellaceae_unclassified | 0.147 | -0.023 | 0.089 |
| s__Subdoligranulum_unclassified | 0.138 | -0.016 | 0.109 | g__Barnesiella | 0.074 | 0.007 | 0.337 | f__Sutterellaceae | 0.098 | -0.017 | 0.218 |
| g__Butyrivibrio | 0.046 | 0.002 | 0.307 | o__Burkholderiales | 0.037 | -0.003 | 0.950 | s__Lachnospiraceae_bacterium_3_1_46FAA | 0.068 | -0.008 | 0.198 |
| g__Bacteroides | 0.029 | -0.002 | 0.960 | s__Bifidobacterium_longum | 0.026 | 0.001 | 0.990 | g__Odoribacter | 0.059 | -0.008 | 0.436 |
| s__Holdemania_filiformis | 0.024 | 0.001 | 0.941 | o__Bacteroidales | 0.026 | 0.001 | 0.970 | g__Bifidobacterium | 0.045 | -0.005 | 0.663 |
| g__Coprococcus | 0.019 | 0 | 0.990 | c__Gammaproteobacteria | 0.024 | 0 | 1.000 | o__Selenomonadales | 0.038 | -0.002 | 0.812 |
| s__Pseudoflavonifractor_capillosus | 0.019 | 0.001 | 0.990 | s__Faecalibacterium_prausnitzii | 0.023 | -0.001 | 0.980 | s__Bacteroides_ovatus | 0.036 | 0.003 | 0.782 |
| g__Alistipes | 0.017 | 0 | 0.990 | g__Coprococcus | 0.023 | -0.001 | 0.980 | s__Holdemania_filiformis | 0.031 | 0.003 | 0.842 |

**Table S10. MR-BMA ranking of microbial biomarkers for malignant tumors based on MIP and MACE in microbial risk factors identifying analysis.**

| **Colorectal cancer** | | | | **Malignant tumors of the colon** | | | | **Malignant tumors of the rectal** | | | |
| --- | --- | --- | --- | --- | --- | --- | --- | --- | --- | --- | --- |
| **Exposure** | **MIP** | **MACE** | **Pval** | **Exposure** | **MIP** | **MACE** | **Pval** | **Exposure** | **MIP** | **MACE** | **Pval** |
| s__Streptococcus_thermophilus | 0.36 | -0.037 | 0.010 | s__Eubacterium_ramulus | 0.719 | 0.137 | 0.010 | s__Ruminococcus_bromii | 0.514 | 0.137 | 0.010 |
| s__Pseudoflavonifractor_capillosus | 0.257 | 0.031 | 0.030 | s__Streptococcus_thermophilus | 0.528 | -0.074 | 0.010 | s__Lachnospiraceae_bacterium_8_1_57FAA | 0.417 | 0.074 | 0.010 |
| s__Lachnospiraceae_bacterium_8_1_57FAA | 0.247 | 0.025 | 0.010 | s__Pseudoflavonifractor_capillosus | 0.269 | 0.04 | 0.020 | f__Erysipelotrichaceae | 0.284 | -0.064 | 0.010 |
| f__Erysipelotrichaceae | 0.138 | -0.018 | 0.040 | s__Eubacterium_ventriosum | 0.219 | -0.029 | 0.010 | o__Selenomonadales | 0.197 | -0.047 | 0.030 |
| s__Paraprevotella_xylaniphila | 0.135 | -0.013 | 0.020 | s__Bilophila_unclassified | 0.129 | 0.024 | 0.030 | s__Bacteroides_vulgatus | 0.125 | 0.028 | 0.020 |
| s__Eubacterium_ramulus | 0.089 | 0.01 | 0.030 | g__Streptococcus | 0.118 | -0.017 | 0.030 | s__Eubacterium_siraeum | 0.106 | -0.021 | 0.059 |
| f__Coriobacteriaceae | 0.043 | 0.005 | 0.069 | f__Streptococcaceae | 0.095 | -0.013 | 0.040 | s__Coprococcus_sp_ART55_1 | 0.103 | -0.011 | 0.020 |
| g__Alistipes | 0.038 | 0.004 | 0.069 | g__Ruminococcus | 0.093 | -0.015 | 0.030 | s__Bacteroides_massiliensis | 0.044 | 0.006 | 0.079 |
| s__Paraprevotella_unclassified | 0.037 | -0.003 | 0.099 | s__Paraprevotella_clara | 0.085 | -0.011 | 0.020 | g__Eubacterium | 0.038 | -0.006 | 0.079 |
| s__Alistipes_senegalensis | 0.033 | 0.004 | 0.109 | s__Alistipes_finegoldii | 0.083 | 0.013 | 0.059 | s__Adlercreutzia_equolifaciens | 0.037 | -0.005 | 0.069 |

**Table S11. MR-BMA ranking of microbial biomarkers for benign tumors based on MIP and MACE in microbial risk factors identifying analysis.**

| **benign tumors of the colorectum** | | | | **benign tumors of the colon** | | | | **benign tumors of the rectal** | | | |
| --- | --- | --- | --- | --- | --- | --- | --- | --- | --- | --- | --- |
| **Exposure** | **MIP** | **MACE** | **Pval** | **Exposure** | **MIP** | **MACE** | **Pval** | **Exposure** | **MIP** | **MACE** | **Pval** |
| s__Holdemania_unclassified | 0.857 | -0.072 | 0.010 | s__Ruminococcus_bromii | 0.337 | -0.036 | 0.010 | s__Parabacteroides_goldsteinii | 0.449 | 0.052 | 0.010 |
| s__Ruminococcus_bromii | 0.274 | -0.026 | 0.020 | s__Holdemania_unclassified | 0.151 | -0.01 | 0.020 | g__Ruminococcus | 0.2 | -0.036 | 0.020 |
| s__Bacteroides_ovatus | 0.178 | 0.017 | 0.020 | s__Escherichia_unclassified | 0.068 | 0.005 | 0.020 | s__Bacteroides_ovatus | 0.136 | 0.022 | 0.040 |
| s__Eubacterium_ventriosum | 0.13 | 0.008 | 0.010 | o__Lactobacillales | 0.052 | 0.003 | 0.050 | s_Bacteroidales_bacterium_ph8 | 0.087 | 0.014 | 0.059 |
| g__Ruminococcus | 0.069 | -0.006 | 0.020 | s_Bacteroides_xylanisolvens | 0.052 | -0.004 | 0.050 | s__Parasutterella_excrementihominis | 0.077 | 0.009 | 0.079 |
| o__Lactobacillales | 0.043 | 0.003 | 0.050 | g__Ruminococcus | 0.051 | -0.004 | 0.030 | s__Holdemania_unclassified | 0.077 | -0.008 | 0.020 |
| s_Bifidobacterium_adolescentis | 0.043 | -0.003 | 0.059 | f__Ruminococcaceae | 0.041 | -0.003 | 0.069 | s__Akkermansia_muciniphila | 0.076 | 0.011 | 0.059 |
| g__Bifidobacterium | 0.035 | -0.002 | 0.040 | s__Subdoligranulum_unclassified | 0.039 | -0.003 | 0.050 | g__Collinsella | 0.072 | 0.011 | 0.020 |
| s__Escherichia_coli | 0.032 | 0.002 | 0.069 | s__Eubacterium_ventriosum | 0.036 | 0.002 | 0.079 | s__Escherichia_coli | 0.061 | 0.007 | 0.089 |
| s_Streptococcus_parasanguinis | 0.022 | 0.001 | 0.119 | s__Bacteroides_ovatus | 0.032 | 0.002 | 0.079 | s_Bacteroides_xylanisolvens | 0.061 | 0.008 | 0.099 |

**Table S12. A detailed list of 194 microorganisms with strongly correlated independent genetic variation in microbial risk factors identifying analysis.**

| s__Bifidobacterium_adolescentis | s__Dorea_formicigenerans | g__Bacteroidales_noname | c__Betaproteobacteria |
| --- | --- | --- | --- |
| s__Bifidobacterium_bifidum | s__Dorea_longicatena | g__Barnesiella | c__Deltaproteobacteria |
| s__Bifidobacterium_longum | s__Lachnospiraceae_bacterium_5_1_63FAA | g__Odoribacter | c__Gammaproteobacteria |
| s__Adlercreutzia_equolifaciens | s__Lachnospiraceae_bacterium_8_1_57FAA | g__Parabacteroides | c__Verrucomicrobiae |
| s__Gordonibacter_pamelaeae | s__Roseburia_intestinalis | g__Paraprevotella | f__Micrococcaceae |
| s__Bacteroides_caccae | s__Roseburia_unclassified | g__Alistipes | f__Bifidobacteriaceae |
| s__Bacteroides_cellulosilyticus | s__Oscillibacter_unclassified | g__Lactobacillus | f__Coriobacteriaceae |
| s__Bacteroides_clarus | s__Faecalibacterium_prausnitzii | g__Flavonifractor | f__Bacteroidaceae |
| s__Bacteroides_dorei | s__Ruminococcaceae_bacterium_D16 | g__Eubacterium | f__Bacteroidales_noname |
| s__Bacteroides_eggerthii | s__Ruminococcus_bromii | g__Blautia | f__Porphyromonadaceae |
| s__Bacteroides_finegoldii | s__Ruminococcus_callidus | g__Butyrivibrio | f__Prevotellaceae |
| s__Bacteroides_fragilis | s__Eubacterium_biforme | g__Dorea | f__Rikenellaceae |
| s__Bacteroides_intestinalis | s__Holdemania_unclassified | g__Lachnospiraceae_noname | f__Lactobacillaceae |
| s__Bacteroides_massiliensis | s__Phascolarctobacterium_succinatutens | g__Roseburia | f__Streptococcaceae |
| s__Bacteroides_ovatus | s__Dialister_invisus | g__Oscillibacter | f__Clostridiaceae |
| s__Bacteroides_salyersiae | s__Veillonella_unclassified | g__Ruminococcus | f__Clostridiales_noname |
| s__Bacteroides_vulgatus | s__Burkholderiales_bacterium_1_1_47 | g__Subdoligranulum | f__Eubacteriaceae |
| s__Bacteroidales_bacterium_ph8 | s__Oxalobacter_formigenes | g__Phascolarctobacterium | f__Lachnospiraceae |
| s__Parabacteroides_distasonis | s__Parasutterella_excrementihominis | g__Dialister | f__Oscillospiraceae |
| s__Parabacteroides_goldsteinii | s__Sutterella_wadsworthensis | g__Veillonella | f__Ruminococcaceae |
| s__Parabacteroides_johnsonii | s__Bilophila_unclassified | g__Burkholderiales_noname | f__Erysipelotrichaceae |
| s__Parabacteroides_merdae | s__Bilophila_wadsworthia | g__Oxalobacter | f__Acidaminococcaceae |
| s__Parabacteroides_unclassified | s__Desulfovibrio_piger | g__Bilophila | f__Veillonellaceae |
| s__Paraprevotella_unclassified | s__Escherichia_coli | g__Desulfovibrio | f__Burkholderiales_noname |
| s__Paraprevotella_xylaniphila | s__Haemophilus_parainfluenzae | g__Escherichia | f__Oxalobacteraceae |
| s__Prevotella_copri | s__Akkermansia_muciniphila | g__Haemophilus | f__Sutterellaceae |
| s__Alistipes_indistinctus | s__Coprobacter_fastidiosus | g__Akkermansia | f__Desulfovibrionaceae |
| s__Alistipes_onderdonkii | s__Odoribacter_splanchnicus | g__Adlercreutzia | f__Enterobacteriaceae |
| s__Alistipes_senegalensis | s__Coprococcus_comes | g__Eggerthella | f__Pasteurellaceae |
| s__Alistipes_shahii | s__Ruminococcus_lactaris | g__Bacteroides | f__Verrucomicrobiaceae |
| s__Alistipes_sp_AP11 | s__Bifidobacterium_catenulatum | g__Coprobacter | o__Actinomycetales |
| s__Lactobacillus_delbrueckii | s__Bacteroides_coprocola | g__Prevotella | o__Bifidobacteriales |
| s__Streptococcus_parasanguinis | s__Bacteroides_plebeius | g__Streptococcus | o__Coriobacteriales |
| s__Streptococcus_salivarius | s__Bacteroides_thetaiotaomicron | g__Clostridium | o__Bacteroidales |
| s__Streptococcus_thermophilus | s__Barnesiella_intestinihominis | g__Pseudoflavonifractor | o__Lactobacillales |
| s__Clostridium_leptum | s__Paraprevotella_clara | g__Coprococcus | o__Clostridiales |
| s__Flavonifractor_plautii | s__Alistipes_finegoldii | g__Faecalibacterium | o__Erysipelotrichales |
| s__Pseudoflavonifractor_capillosus | s__Lachnospiraceae_bacterium_7_1_58FAA | g__Ruminococcaceae_noname | o__Selenomonadales |
| s__Eubacterium_eligens | s__Roseburia_hominis | g__Erysipelotrichaceae_noname | o__Burkholderiales |
| s__Eubacterium_hallii | s__Subdoligranulum_unclassified | g__Holdemania | o__Desulfovibrionales |
| s__Eubacterium_ramulus | s_Bacteroides_stercoris | g__Parasutterella | o__Enterobacteriales |
| s__Eubacterium_rectale | s_Bacteroides_xylanisolvens | g__Sutterella | o__Pasteurellales |
| s__Eubacterium_siraeum | s__Bacteroides_faecis | g__Sutterellaceae_unclassified | o__Verrucomicrobiales |
| s__Eubacterium_ventriosum | s__Clostridium_asparagiforme | c__Actinobacteria | p__Actinobacteria |
| s__Ruminococcus_obeum | s__Escherichia_unclassified | c__Bacteroidia | p__Bacteroidetes |
| s__Ruminococcus_torques | g__Rothia | c__Bacilli | p__Proteobacteria |
| s__Butyrivibrio_crossotus | g__Bifidobacterium | c__Clostridia | p__Verrucomicrobia |
| s__Coprococcus_catus | g__Collinsella | c__Erysipelotrichia |  |
| s__Coprococcus_sp_ART55_1 | g__Gordonibacter | c__Negativicutes |  |

**Table S13. In the analysis of potential biomarkers ranking, after excluding outliers and influential variants, the combined model (risk factor set) of colorectal cancer and BCR is ranked according to its posterior probability (*pp*).**

| **CRC** | | | **BRC** | | |
| --- | --- | --- | --- | --- | --- |
| **Exposure** | **pp** | **Model-specific causal estimates** | **Exposure** | **pp** | **Model-specific causal estimates** |
| g__Pseudoflavonifractor, s__Streptococcus_thermophilus | 0.223 | 0.291, -0.192 | o__Bacteroidales | 0.296 | 0.148 |
| g__Pseudoflavonifractor, s__Coprococcus_catus | 0.137 | 0.271, -0.279 | s__Bifidobacterium_adolescentis | 0.183 | -0.142 |
| g__Pseudoflavonifractor | 0.121 | 0.278 | s__Bacteroides_ovatus | 0.098 | 0.132 |
| g__Pseudoflavonifractor, s__Coprococcus_catus, s__Streptococcus_thermophilus | 0.114 | 0.283, -0.239, -0.17 | s__Subdoligranulum_unclassified | 0.083 | -0.124 |
| s__Coprococcus_catus | 0.029 | -0.29 | s__Bacteroides_ovatus, s__Bifidobacterium_adolescentis | 0.074 | 0.119, -0.129 |
| g__Pseudoflavonifractor,f __Erysipelotrichaceae,s__Streptococcus_thermophilus | 0.025 | 0.309, -0.176, -0.197 | g__Butyrivibrio, o__Bacteroidales | 0.023 | 0.058, 0.177 |
| g__Pseudoflavonifractor, s__Parabacteroides_distasonis, s__Streptococcus_thermophilus | 0.019 | 0.299, 0.148, -0.203 | o__Bacteroidales, s__Bacteroides_ovatus | 0.023 | 0.116, 0.092 |
| s__Streptococcus_thermophilus | 0.019 | -0.18 | s__Subdoligranulum_unclassified, s__Bifidobacterium_adolescentis | 0.016 | -0.091, -0.112 |
| g__Pseudoflavonifractor, s__Coprococcus_catus, s__Parabacteroides_distasonis, s__Streptococcus_thermophilus | 0.017 | 0.292, -0.254, 0.166, -0.182 | o__Bacteroidales, s__Bifidobacterium_adolescentis | 0.015 | 0.102, -0.089 |
| g__Streptococcus, g__Pseudoflavonifractor | 0.014 | -0.145,0.277 | s__Subdoligranulum_unclassified, s__Bacteroides_ovatus | 0.009 | -0.094, 0.101 |

**Table S14. In the analysis of potential risk factor ranking, after excluding outliers and influential variants, the combined model (risk factor set) of CC and BC is ranked according to its posterior probability (*pp*).**

| **CC** | | | | **BC** | | |
| --- | --- | --- | --- | --- | --- | --- |
| **Exposure** | **pp** | **Model-specific causal estimates** | **Exposure** | | **pp** | **Model-specific causal estimates** |
| c__Gammaproteobacteria | 0.075 | 0.237 | s__Bacteroides_ovatus | | 0.623 | 0.152 |
| s__Eubacterium_ventriosum | 0.06 | -0.147 | g__Barnesiella | | 0.039 | 0.107 |
| s__Sutterella_wadsworthensis | 0.053 | -0.176 | s__Bacteroides_ovatus, s__Bifidobacterium_adolescentis | | 0.035 | 0.151, -0.092 |
| s__Holdemania_unclassified | 0.045 | -0.127 | s__Bacteroides_ovatus, s__Lachnospiraceae_bacterium_5_1_63FAA | | 0.031 | 0.146, 0.056 |
| s__Prevotella_copri | 0.03 | 0.129 | s__Lachnospiraceae_bacterium_5_1_63FAA | | 0.027 | 0.061 |
| c__Betaproteobacteria | 0.029 | -0.139 | s__Bifidobacterium_adolescentis | | 0.023 | -0.094 |
| s__Paraprevotella_xylaniphila | 0.027 | -0.107 | g__Barnesiella, s__Bacteroides_ovatus | | 0.02 | 0.075, 0.137 |
| s__Roseburia_inulinivorans | 0.027 | 0.136 | o__Burkholderiales | | 0.017 | -0.087 |
| f__Sutterellaceae | 0.025 | -0.128 | o__Burkholderiales, s__Bacteroides_ovatus | | 0.013 | -0.054, 0.144 |
| s__Parabacteroides_goldsteinii | 0.023 | 0.086 | o__Bacteroidales | | 0.011 | 0.065 |

**Table S15. In the analysis of potential risk factor ranking, after excluding outliers and influential variants, the combined model (risk factor set) of CR and BR is ranked according to its posterior probability (*pp*).**

| **CR** | | | **BR** | | |
| --- | --- | --- | --- | --- | --- |
| **Exposure** | **pp** | **Model-specific causal estimates** | **Exposure** | **pp** | **Model-specific causal estimates** |
| s__Coprococcus_sp_ART55_1 | 0.179 | -0.213 | f__Acidaminococcaceae | 0.289 | -0.203 |
| g__Desulfovibrio | 0.138 | -0.327 | f__Acidaminococcaceae, s__Eubacterium_rectale | 0.132 | -0.213, -0.243 |
| f__Erysipelotrichaceae | 0.04 | -0.257 | f__Acidaminococcaceae, g__Sutterellaceae_unclassified | 0.041 | -0.203, -0.15 |
| g__Desulfovibrio, s__Coprococcus_sp_ART55_1 | 0.038 | -0.261, -0.181 | s__Eubacterium_rectale | 0.032 | -0.222 |
| f__Erysipelotrichaceae, s__Coprococcus_sp_ART55_1 | 0.028 | -0.238, -0.206 | f__Acidaminococcaceae, s__Eubacterium_rectale, g__Sutterellaceae_unclassified | 0.029 | -0.214, -0.257, -0.161 |
| f__Erysipelotrichaceae, g__Desulfovibrio | 0.021 | -0.237, -0.312 | f__Sutterellaceae, f__Acidaminococcaceae | 0.023 | -0.159, -0.202 |
| s__Coprococcus_catus | 0.016 | -0.191 | f__Sutterellaceae, f__Acidaminococcaceae, s__Eubacterium_rectale | 0.023 | -0.198, -0.213, -0.271 |
| g__Dorea | 0.014 | -0.17 | f__Acidaminococcaceae, s__Lachnospiraceae_bacterium_3_1_46FAA | 0.021 | -0.201, -0.129 |
| g__Barnesiella | 0.012 | 0.154 | g__Sutterellaceae_unclassified | 0.017 | -0.149 |
| s__Streptococcus_thermophilus | 0.012 | -0.136 | f__Acidaminococcaceae, g__Odoribacter | 0.016 | -0.205, -0.138 |

**Table S16. In the analysis of microbial risk factors identifying, after excluding outliers and influential variants, the combined model (risk factor set) of CRC and BCR is ranked according to its posterior probability (pp).**

| **CRC** | | | **BRC** | | |
| --- | --- | --- | --- | --- | --- |
| **Exposure** | **pp** | **Model-specific causal estimates** | **Exposure** | **pp** | **Model-specific causal estimates** |
| g__Pseudoflavonifractor, s__Streptococcus_thermophilus | 0.223 | 0.291, -0.192 | o__Bacteroidales | 0.296 | 0.148 |
| g__Pseudoflavonifractor, s__Coprococcus_catus | 0.137 | 0.271, -0.279 | s__Bifidobacterium_adolescentis | 0.183 | -0.142 |
| g__Pseudoflavonifractor | 0.121 | 0.278 | s__Bacteroides_ovatus | 0.098 | 0.132 |
| g__Pseudoflavonifractor, s__Coprococcus_catus, s__Streptococcus_thermophilus | 0.114 | 0.283, -0.239, -0.17 | s__Subdoligranulum_unclassified | 0.083 | -0.124 |
| s__Coprococcus_catus | 0.029 | -0.29 | s__Bacteroides_ovatus, s__Bifidobacterium_adolescentis | 0.074 | 0.119, -0.129 |
| g__Pseudoflavonifractor,f __Erysipelotrichaceae,s__Streptococcus_thermophilus | 0.025 | 0.309, -0.176, -0.197 | g__Butyrivibrio, o__Bacteroidales | 0.023 | 0.058, 0.177 |
| g__Pseudoflavonifractor, s__Parabacteroides_distasonis, s__Streptococcus_thermophilus | 0.019 | 0.299, 0.148, -0.203 | o__Bacteroidales, s__Bacteroides_ovatus | 0.023 | 0.116, 0.092 |
| s__Streptococcus_thermophilus | 0.019 | -0.18 | s__Subdoligranulum_unclassified, s__Bifidobacterium_adolescentis | 0.016 | -0.091, -0.112 |
| g__Pseudoflavonifractor, s__Coprococcus_catus, s__Parabacteroides_distasonis, s__Streptococcus_thermophilus | 0.017 | 0.292, -0.254, 0.166, -0.182 | o__Bacteroidales, s__Bifidobacterium_adolescentis | 0.015 | 0.102, -0.089 |
| g__Streptococcus, g__Pseudoflavonifractor | 0.014 | -0.145,0.277 | s__Subdoligranulum_unclassified, s__Bacteroides_ovatus | 0.009 | -0.094, 0.101 |

**Table S17. In the analysis of microbial risk factors identifying, after excluding outliers and influential variants, the combined model (risk factor set) of CC and BC is ranked according to its posterior probability (pp).**

| **CRC** | | | **BRC** | | |
| --- | --- | --- | --- | --- | --- |
| **Exposure** | **pp** | **Model-specific causal estimates** | **Exposure** | **pp** | **Model-specific causal estimates** |
| s__Streptococcus_thermophilus, s__Eubacterium_ramulus | 0.01 | -0.147,0.181 | s__Ruminococcus_bromii | 0.057 | -0.102 |
| s__Streptococcus_thermophilus, s__Pseudoflavonifractor_capillosus, s__Eubacterium_ramulus | 0.007 | -0.151,0.15,0.182 | s__Holdemania_unclassified | 0.02 | -0.063 |
| s__Streptococcus_thermophilus | 0.005 | -0.128 | s__Ruminococcus_bromii, s__Holdemania_unclassified | 0.011 | -0.104, -0.064 |
| s__Streptococcus_thermophilus, s__Eubacterium_ramulus, s__Eubacterium_ventriosum | 0.004 | -0.144, 0.199, -0.132 | s__Escherichia_unclassified | 0.01 | 0.075 |
| s__Eubacterium_ramulus, g__Streptococcus | 0.004 | 0.187, -0.155 | g__Ruminococcus | 0.01 | -0.078 |
| s__Streptococcus_thermophilus, s__Pseudoflavonifractor_capillosus, s__Eubacterium_ramulus, s__Eubacterium_ventriosum | 0.003 | -0.148, 0.154, 0.2, -0.137 | s_Bacteroides_xylanisolvens | 0.009 | -0.08 |
| s__Eubacterium_ramulus, f__Streptococcaceae | 0.003 | 0.187, -0.153 | s__Subdoligranulum_unclassified | 0.008 | -0.074 |
| s__Eubacterium_ramulus | 0.003 | 0.154 | f__Ruminococcaceae | 0.007 | -0.072 |
| ,s__Streptococcus_thermophilus, s__Pseudoflavonifractor_capillosus | 0.003 | -0.132,0.149 | s__Ruminococcus_bromii, s__Escherichia_unclassified | 0.006 | -0.106, 0.079 |
| s__Streptococcus_thermophilus, s__Eubacterium_ramulus, s__Bilophila_unclassified | 0.002 | -0.137,0.187,0.169 | o__Lactobacillales | 0.005 | 0.058 |

**Table S18. In the analysis of microbial risk factors identifying, after excluding outliers and influential variants, the combined model (risk factor set) of CR and BR is ranked according to its posterior probability (pp).**

| **CRC** | | | **BRC** | | |
| --- | --- | --- | --- | --- | --- |
| **Exposure** | **pp** | **Model-specific causal estimates** | **Exposure** | **pp** | **Model-specific causal estimates** |
| s__Ruminococcus_bromii | 0.007 | 0.243 | s__Parabacteroides_goldsteinii | 0.026 | 0.114 |
| s__Lachnospiraceae_bacterium_8_1_57FAA | 0.006 | 0.166 | s__Bacteroides_ovatus | 0.006 | 0.155 |
| s__Lachnospiraceae_bacterium_8_1_57FAA,s__Ruminococcus_bromii,f__Erysipelotrichaceae | 0.004 | 0.182,0.254,-0.257 | s__Parabacteroides_goldsteinii,g__Ruminococcus | 0.006 | 0.122,-0.152 |
| s__Lachnospiraceae_bacterium_8_1_57FAA,f__Erysipelotrichaceae | 0.004 | 0.196,-0.225 | s__Parasutterella_excrementihominis | 0.005 | 0.126 |
| s__Ruminococcus_bromii,f__Erysipelotrichaceae | 0.003 | 0.278,-0.212 | s__Akkermansia_muciniphila | 0.004 | 0.14 |
| s__Ruminococcus_bromii,o__Selenomonadales | 0.003 | 0.26,-0.248 | s__Bacteroidales_bacterium_ph8 | 0.004 | 0.156 |
| o__Selenomonadales | 0.003 | -0.225 | s__Parabacteroides_goldsteinii,s__Holdemania_unclassified | 0.004 | 0.123,-0.1 |
| s__Bacteroides_vulgatus | 0.002 | 0.225 | g__Ruminococcus | 0.004 | -0.131 |
| s__Eubacterium_siraeum,s__Ruminococcus_bromii | 0.002 | -0.221,0.268 | s_Bacteroides_xylanisolvens | 0.003 | 0.135 |
| s__Lachnospiraceae_bacterium_8_1_57FAA,o__Selenomonadales | 0.002 | 0.171,-0.237 | s__Escherichia_coli | 0.003 | 0.11 |

**Table S19. Results of Mendelian randomization analysis of dietary habits with different niches and different periods of colorectal cancer.**

| Outcome | Exposure | Method | nsnp | b | se | pval | p.adjust |
| --- | --- | --- | --- | --- | --- | --- | --- |
| CC | Alcohol drinker status: Current | IVW(M) | 3 | 11.03 | 5.44 | 0.04 | 0.18 |
| CR | Alcohol drinker status: Current | IVW(M) | 3 | -14.16 | 7.82 | 0.07 | 0.25 |
| BCR | Alcohol drinker status: Current | IVW(M) | 3 | 4.74 | 4.45 | 0.29 | 0.60 |
| BC | Alcohol drinker status: Current | IVW(M) | 3 | 5.41 | 6.48 | 0.40 | 0.65 |
| BR | Alcohol drinker status: Current | IVW(M) | 3 | -1.90 | 4.58 | 0.68 | 0.91 |
| CRC | Alcohol drinker status: Current | IVW(M) | 3 | 1.67 | 4.78 | 0.73 | 0.92 |
| BCR | Bread intake | IVW(M) | 25 | 0.54 | 0.23 | 0.02 | 0.10 |
| BC | Bread intake | IVW(M) | 25 | 0.49 | 0.27 | 0.07 | 0.24 |
| BR | Bread intake | IVW(M) | 25 | 0.52 | 0.42 | 0.21 | 0.48 |
| CRC | Bread intake | IVW(M) | 25 | -0.06 | 0.58 | 0.92 | 0.97 |
| CC | Bread intake | IVW(M) | 25 | 0.02 | 0.71 | 0.98 | 0.99 |
| CR | Bread intake | IVW(M) | 25 | 0.02 | 0.76 | 0.98 | 0.99 |
| CRC | Cereal type: Biscuit cereal (e.g. Weetabix) | IVW(M) | 2 | -2.99 | 0.07 | 0 | 0 |
| CR | Cereal type: Biscuit cereal (e.g. Weetabix) | IVW(M) | 2 | -7.21 | 5.92 | 0.22 | 0.49 |
| BCR | Cereal type: Biscuit cereal (e.g. Weetabix) | IVW(M) | 2 | -1.68 | 1.75 | 0.34 | 0.64 |
| BC | Cereal type: Biscuit cereal (e.g. Weetabix) | IVW(M) | 2 | -0.82 | 0.94 | 0.38 | 0.65 |
| BR | Cereal type: Biscuit cereal (e.g. Weetabix) | IVW(M) | 2 | -1.33 | 3.97 | 0.74 | 0.92 |
| CC | Cereal type: Biscuit cereal (e.g. Weetabix) | IVW(M) | 2 | 0.97 | 3.57 | 0.79 | 0.92 |
| CRC | Ferritin | IVW(M) | 3 | 0.27 | 0.01 | 2.30E-168 | 1.17E-166 |
| BR | Ferritin | IVW(M) | 3 | 0.35 | 0.06 | 8.42E-09 | 1.23E-07 |
| CR | Ferritin | IVW(M) | 3 | 0.35 | 0.37 | 0.34 | 0.64 |
| CC | Ferritin | IVW(M) | 3 | 0.12 | 0.13 | 0.35 | 0.64 |
| BC | Ferritin | IVW(M) | 3 | -0.03 | 0.20 | 0.89 | 0.97 |
| BCR | Ferritin | IVW(M) | 3 | 0.02 | 0.15 | 0.91 | 0.97 |
| CR | Lamb/mutton intake | IVW(M) | 30 | -2.19 | 0.83 | 0.01 | 0.05 |
| CRC | Lamb/mutton intake | IVW(M) | 30 | -0.42 | 0.48 | 0.38 | 0.65 |
| CC | Lamb/mutton intake | IVW(M) | 30 | 0.48 | 0.67 | 0.47 | 0.74 |
| BCR | Lamb/mutton intake | IVW(M) | 30 | -0.13 | 0.31 | 0.67 | 0.91 |
| BC | Lamb/mutton intake | IVW(M) | 30 | -0.15 | 0.39 | 0.71 | 0.92 |
| BR | Lamb/mutton intake | IVW(M) | 30 | 0.20 | 0.65 | 0.75 | 0.92 |
| CC | Liver intake | IVW(M) | 2 | 0.84 | 0.05 | 5.38E-58 | 1.83E-56 |
| BR | Liver intake | IVW(M) | 2 | 1.68 | 0.33 | 2.23E-07 | 2.28E-06 |
| CRC | Liver intake | IVW(M) | 2 | 0.55 | 1.57 | 0.73 | 0.92 |
| BCR | Liver intake | IVW(M) | 2 | -0.24 | 0.79 | 0.76 | 0.92 |
| CR | Liver intake | IVW(M) | 2 | -0.53 | 3.38 | 0.88 | 0.97 |
| BC | Liver intake | IVW(M) | 2 | 0.07 | 0.53 | 0.90 | 0.97 |
| BC | Milk type used: Never/rarely have milk | IVW(M) | 2 | 8.28 | 1.43 | 6.71E-09 | 1.14E-07 |
| BCR | Milk type used: Never/rarely have milk | IVW(M) | 2 | 5.39 | 1.81 | 2.97E-03 | 0.02 |
| CC | Milk type used: Never/rarely have milk | IVW(M) | 2 | 9.33 | 3.98 | 0.02 | 0.10 |
| BR | Milk type used: Never/rarely have milk | IVW(M) | 2 | -7.25 | 9.58 | 0.45 | 0.72 |
| CRC | Milk type used: Never/rarely have milk | IVW(M) | 2 | 6.42 | 9.20 | 0.49 | 0.75 |
| CR | Milk type used: Never/rarely have milk | IVW(M) | 2 | 1.85 | 17.37 | 0.92 | 0.97 |
| BCR | Mineral and other dietary supplements: Calcium | IVW(M) | 2 | 2.86 | 0.18 | 2.83E-55 | 7.21E-54 |
| BR | Mineral and other dietary supplements: Calcium | IVW(M) | 2 | 4.05 | 1.02 | 7.55E-05 | 6.42E-04 |
| BC | Mineral and other dietary supplements: Calcium | IVW(M) | 2 | 4.69 | 1.60 | 3.36E-03 | 0.02 |
| CRC | Mineral and other dietary supplements: Calcium | IVW(M) | 2 | -1.06 | 0.61 | 0.08 | 0.27 |
| CC | Mineral and other dietary supplements: Calcium | IVW(M) | 2 | -4.59 | 4.15 | 0.27 | 0.57 |
| CR | Mineral and other dietary supplements: Calcium | IVW(M) | 2 | 3.72 | 6.44 | 0.56 | 0.84 |
| BR | Mineral and other dietary supplements: Glucosamine | IVW(M) | 2 | 2.66 | 1.29 | 0.04 | 0.17 |
| BC | Mineral and other dietary supplements: Glucosamine | IVW(M) | 2 | -4.31 | 2.15 | 0.05 | 0.18 |
| CRC | Mineral and other dietary supplements: Glucosamine | IVW(M) | 2 | 1.39 | 0.92 | 0.13 | 0.38 |
| CR | Mineral and other dietary supplements: Glucosamine | IVW(M) | 2 | 3.80 | 2.84 | 0.18 | 0.46 |
| BCR | Mineral and other dietary supplements: Glucosamine | IVW(M) | 2 | -3.05 | 2.36 | 0.20 | 0.48 |
| CC | Mineral and other dietary supplements: Glucosamine | IVW(M) | 2 | 1.01 | 3.50 | 0.77 | 0.92 |
| BCR | Mineral and other dietary supplements: Zinc | IVW(M) | 3 | 3.86 | 0.35 | 6.83E-28 | 1.39E-26 |
| CC | Mineral and other dietary supplements: Zinc | IVW(M) | 3 | -6.91 | 4.02 | 0.09 | 0.28 |
| BC | Mineral and other dietary supplements: Zinc | IVW(M) | 3 | 1.68 | 1.02 | 0.10 | 0.30 |
| CRC | Mineral and other dietary supplements: Zinc | IVW(M) | 3 | -1.82 | 1.78 | 0.31 | 0.63 |
| BR | Mineral and other dietary supplements: Zinc | IVW(M) | 3 | 5.63 | 5.86 | 0.34 | 0.64 |
| CR | Mineral and other dietary supplements: Zinc | IVW(M) | 3 | -0.15 | 11.91 | 0.99 | 0.99 |
| CR | Single crust pastry intake | IVW(M) | 2 | 5.57 | 2.77 | 0.04 | 0.18 |
| CC | Single crust pastry intake | IVW(M) | 2 | -2.97 | 2.12 | 0.16 | 0.42 |
| BC | Single crust pastry intake | IVW(M) | 2 | 0.58 | 0.91 | 0.52 | 0.80 |
| CRC | Single crust pastry intake | IVW(M) | 2 | -0.80 | 2.21 | 0.72 | 0.92 |
| BCR | Single crust pastry intake | IVW(M) | 2 | -0.36 | 0.90 | 0.69 | 0.92 |
| BR | Single crust pastry intake | IVW(M) | 2 | -0.30 | 3.73 | 0.94 | 0.98 |
| CR | Type of special diet followed: Gluten-free | IVW(M) | 2 | -6.55 | 1.15 | 1.41E-08 | 1.80E-07 |
| CC | Type of special diet followed: Gluten-free | IVW(M) | 2 | 5.39 | 0.96 | 1.79E-08 | 2.02E-07 |
| BCR | Type of special diet followed: Gluten-free | IVW(M) | 2 | 2.54 | 1.69 | 0.13 | 0.38 |
| BR | Type of special diet followed: Gluten-free | IVW(M) | 2 | 9.20 | 6.99 | 0.19 | 0.47 |
| BC | Type of special diet followed: Gluten-free | IVW(M) | 2 | 2.80 | 2.85 | 0.33 | 0.64 |
| CRC | Type of special diet followed: Gluten-free | IVW(M) | 2 | -0.01 | 0.19 | 0.96 | 0.99 |
| BC | Vitamin and mineral supplements: Multivitamins +/- minerals | IVW(M) | 2 | 2.62 | 0.67 | 8.79E-05 | 6.90E-04 |
| BCR | Vitamin and mineral supplements: Multivitamins +/- minerals | IVW(M) | 2 | 3.16 | 1.20 | 0.01 | 0.05 |
| BR | Vitamin and mineral supplements: Multivitamins +/- minerals | IVW(M) | 2 | 4.26 | 3.51 | 0.23 | 0.49 |
| CC | Vitamin and mineral supplements: Multivitamins +/- minerals | IVW(M) | 2 | -2.65 | 2.75 | 0.34 | 0.64 |
| CRC | Vitamin and mineral supplements: Multivitamins +/- minerals | IVW(M) | 2 | -2.42 | 5.22 | 0.64 | 0.90 |
| CR | Vitamin and mineral supplements: Multivitamins +/- minerals | IVW(M) | 2 | -1.93 | 6.62 | 0.77 | 0.92 |
| CC | Vitamin and mineral supplements: Vitamin C | Wald ratio | 1 | 5.61 | 10.59 | 0.60 | 0.87 |
| CRC | Vitamin and mineral supplements: Vitamin C | Wald ratio | 1 | 13.58 | 8.29 | 0.10 | 0.30 |
| CR | Vitamin and mineral supplements: Vitamin C | Wald ratio | 1 | 31.68 | 13.64 | 0.02 | 0.10 |
| BC | Vitamin and mineral supplements: Vitamin C | Wald ratio | 1 | 7.94 | 5.42 | 0.14 | 0.38 |
| BCR | Vitamin and mineral supplements: Vitamin C | Wald ratio | 1 | 4.08 | 4.89 | 0.40 | 0.65 |
| BR | Vitamin and mineral supplements: Vitamin C | Wald ratio | 1 | 12.30 | 9.78 | 0.21 | 0.48 |

*Note:* CRC, colorectal cancer; CC, malignant tumors of colon; CR, malignant tumors of rectal; BCR, benign tumors of colorectum; BC, benign tumors of colon; BR, benign tumors of rectal; IVW(M), Inverse variance weighted (multiplicative random effects); nsnp, the number of IVs; 95%CI or X95.CI, OR (95% confidence interval); p.adjust, the *P*-value corrected for the false discovery rate (*FDR*) was derived using the Benjamini-Hochberg (*BH*) method.

**Table S20. Results of a Mendelian randomization analysis of dietary habits and 8 gut bacteria.**

| **Outcome** | **Exposure** | **Method** | **nsnp** | **b** | **se** | **pval** | **p.adjust** |
| --- | --- | --- | --- | --- | --- | --- | --- |
| s__Eubacterium_siraeum | Mineral and other dietary supplements: Calcium | IVW(M) | 2 | -4.01 | 0.09 | 0 | 0 |
| s__Eubacterium_ventriosum | Vitamin and mineral supplements: Multivitamins +/- minerals | IVW(M) | 2 | 2.83 | 0.01 | 0 | 0 |
| s__Eubacterium_siraeum | Mineral and other dietary supplements: Glucosamine | IVW(M) | 2 | -2.18 | 0.08 | 8.35E-154 | 1.14E-152 |
| s__Streptococcus_thermophilus | Mineral and other dietary supplements: Zinc | IVW(M) | 2 | 14.51 | 0.88 | 4.25E-61 | 4.35E-60 |
| g__Sutterellaceae_unclassified | Alcohol drinker status: Current | IVW(M) | 2 | 6.29 | 0.41 | 2.45E-54 | 2.01E-53 |
| s__Bifidobacterium_adolescentis | Mineral and other dietary supplements: Zinc | IVW(M) | 2 | -8.69 | 0.64 | 3.71E-42 | 2.53E-41 |
| g__Streptococcus | Mineral and other dietary supplements: Zinc | IVW(M) | 2 | -4.18 | 0.42 | 7.87E-24 | 4.61E-23 |
| g__Sutterellaceae_unclassified | Mineral and other dietary supplements: Glucosamine | IVW(M) | 2 | 3.42 | 0.40 | 2.48E-17 | 1.27E-16 |
| s__Bifidobacterium_adolescentis | Milk type used: Never/rarely have milk | Wald ratio | 1 | 51.02 | 6.82 | 7.06E-14 | 3.22E-13 |
| f__Erysipelotrichaceae | Vitamin and mineral supplements: Multivitamins +/- minerals | IVW(M) | 2 | 1.43 | 0.20 | 3.46E-13 | 1.42E-12 |
| s__Eubacterium_siraeum | Alcohol drinker status: Current | IVW(M) | 2 | 9.68 | 1.63 | 2.85E-09 | 1.06E-08 |
| s__Eubacterium_ventriosum | Alcohol drinker status: Current | IVW(M) | 2 | -19.24 | 3.87 | 6.51E-07 | 2.22E-06 |
| s__Coprococcus_sp_ART55_1 | Ferritin | IVW(M) | 2 | 0.48 | 0.13 | 1.69E-04 | 4.95E-04 |
| s__Streptococcus_thermophilus | Lamb/mutton intake | IVW(M) | 27 | 1.94 | 0.53 | 2.74E-04 | 7.03E-04 |
| g__Sutterellaceae_unclassified | Ferritin | IVW(M) | 2 | 0.72 | 0.21 | 4.50E-04 | 1.09E-03 |
| s__Bacteroides_ovatus | Vitamin and mineral supplements: Multivitamins +/- minerals | IVW(M) | 2 | 2.03 | 0.61 | 8.39E-04 | 1.91E-03 |
| s__Eubacterium_siraeum | Vitamin and mineral supplements: Multivitamins +/- minerals | IVW(M) | 2 | 4.40 | 1.36 | 1.23E-03 | 2.66E-03 |
| s__Coprococcus_sp_ART55_1 | Cereal type: Biscuit cereal (e.g. Weetabix) | IVW(M) | 2 | 2.45 | 0.81 | 2.42E-03 | 4.96E-03 |
| s__Streptococcus_thermophilus | Ferritin | IVW(M) | 3 | -0.42 | 0.15 | 4.21E-03 | 0.01 |
| s__Bacteroides_ovatus | Bread intake | IVW(M) | 27 | 0.63 | 0.22 | 0.01 | 0.01 |
| g__Streptococcus | Milk type used: Never/rarely have milk | Wald ratio | 1 | 26.29 | 9.68 | 0.01 | 0.01 |
| s__Coprococcus_sp_ART55_1 | Milk type used: Never/rarely have milk | Wald ratio | 1 | -44.31 | 16.54 | 0.01 | 0.01 |
| s__Bifidobacterium_adolescentis | Alcohol drinker status: Current | IVW(M) | 2 | 6.10 | 2.48 | 0.01 | 0.02 |
| s__Coprococcus_sp_ART55_1 | Vitamin and mineral supplements: Vitamin C | Wald ratio | 1 | 32.35 | 13.37 | 0.02 | 0.02 |
| s__Bacteroides_ovatus | Mineral and other dietary supplements: Calcium | IVW(M) | 2 | 2.99 | 1.26 | 0.02 | 0.03 |
| s__Eubacterium_ventriosum | Liver intake | Wald ratio | 1 | 8.90 | 3.89 | 0.02 | 0.03 |
| g__Sutterellaceae_unclassified | Mineral and other dietary supplements: Zinc | IVW(M) | 2 | 3.95 | 1.75 | 0.02 | 0.03 |
| s__Eubacterium_ventriosum | Mineral and other dietary supplements: Calcium | IVW(M) | 2 | -2.91 | 1.35 | 0.03 | 0.04 |
| s__Eubacterium_siraeum | Lamb/mutton intake | IVW(M) | 28 | -0.62 | 0.29 | 0.03 | 0.04 |
| g__Streptococcus | Lamb/mutton intake | IVW(M) | 28 | 0.95 | 0.45 | 0.03 | 0.04 |
| s__Coprococcus_sp_ART55_1 | Single crust pastry intake | Wald ratio | 1 | 6.66 | 3.17 | 0.04 | 0.04 |
| s__Bifidobacterium_adolescentis | Single crust pastry intake | Wald ratio | 1 | -2.72 | 1.30 | 0.04 | 0.04 |
| f__Erysipelotrichaceae | Cereal type: Biscuit cereal (e.g. Weetabix) | IVW(M) | 2 | 2.44 | 1.19 | 0.04 | 0.04 |
| g__Streptococcus | Type of special diet followed: Gluten-free | Wald ratio | 1 | 6.27 | 3.05 | 0.04 | 0.04 |
| f__Erysipelotrichaceae | Type of special diet followed: Gluten-free | Wald ratio | 1 | -4.71 | 2.33 | 0.04 | 0.05 |
| s__Coprococcus_sp_ART55_1 | Mineral and other dietary supplements: Calcium | IVW(M) | 2 | -8.87 | 4.44 | 0.05 | 0.05 |
| s__Eubacterium_siraeum | Milk type used: Never/rarely have milk | Wald ratio | 1 | -14.18 | 7.18 | 0.05 |  |

*Note:* CRC, colorectal cancer; CC, malignant tumors of colon; CR, malignant tumors of rectal; BCR, benign tumors of colorectum; BC, benign tumors of colon; BR, benign tumors of rectal; IVW(M), Inverse variance weighted (multiplicative random effects); nsnp, the number of IVs; 95%CI or X95.CI, OR (95% confidence interval); p.adjust, the *P*-value corrected for the false discovery rate (*FDR*) was derived using the Benjamini-Hochberg (*BH*) method.
